# Absence of Tangentially Migrating Glutamatergic Neurons in the Developing Avian Brain

**DOI:** 10.1016/j.celrep.2017.12.032

**Published:** 2018-01-02

**Authors:** Fernando García-Moreno, Edward Anderton, Marta Jankowska, Jo Begbie, Juan Manuel Encinas, Manuel Irimia, Zoltán Molnár

**Affiliations:** 1 Department of Physiology, Anatomy and Genetics, University of Oxford, Oxford OX1 3QX, UK; 2 Achucarro Basque Center for Neuroscience, Parque Científico UPV/EHU Edif. Sede, 48940 Leioa, Spain; 3 IKERBASQUE Foundation, María Díaz de Haro 3, 6th Floor, 48013 Bilbao, Spain; 4 College of Inter-Faculty Individual Studies in Mathematics and Natural Sciences, University of Warsaw, Banacha 2C, 02-097 Warsaw, Poland; 5 Faculty of Biology, University of Warsaw, Ilji Miecznikowa 1, 02-096 Warsaw, Poland; 6 EMBL/CRG Systems Biology Research Unit, Centre for Genomic Regulation (CRG), Barcelona Institute for Science and Technology, 08003 Barcelona, Spain; 7 Universitat Pompeu Fabra (UPF), 08003 Barcelona, Spain

**Keywords:** neocortex, chick, pallium, ventral pallium, evo-devo, evolution, Dbx1, telencephalon

## Abstract

Several neuronal populations orchestrate neocortical development during mammalian embryogenesis. These include the glutamatergic subplate-, Cajal-Retzius-, and ventral pallium-derived populations, which coordinate cortical wiring, migration, and proliferation, respectively. These transient populations are primarily derived from other non-cortical pallial sources that migrate to the dorsal pallium. Are these migrations to the dorsal pallium conserved in amniotes or are they specific to mammals? Using *in ovo* electroporation, we traced the entire lineage of defined chick telencephalic progenitors. We found that several pallial sources that produce tangential migratory neurons in mammals only produced radially migrating neurons in the avian brain. Moreover, ectopic expression of VP-specific mammalian Dbx1 in avian brains altered neurogenesis but did not convert the migration into a mammal-like tangential movement. Together, these data indicate that tangential cellular contributions of glutamatergic neurons originate from outside the dorsal pallium and that pallial Dbx1 expression may underlie the generation of the mammalian neocortex during evolution.

## Highlights

• Lack of tangential migration of Cajal-Retzius and subplate cells in chick
• No intra-pallial tangential migrations contribute to the dorsal pallium in chick
• Mammalian Dbx1 promotes differentiative divisions in chick ventral pallium
• Unique tangential migrations could have played a major role in cortical evolution

## Graphical Abstract

**Figure:**
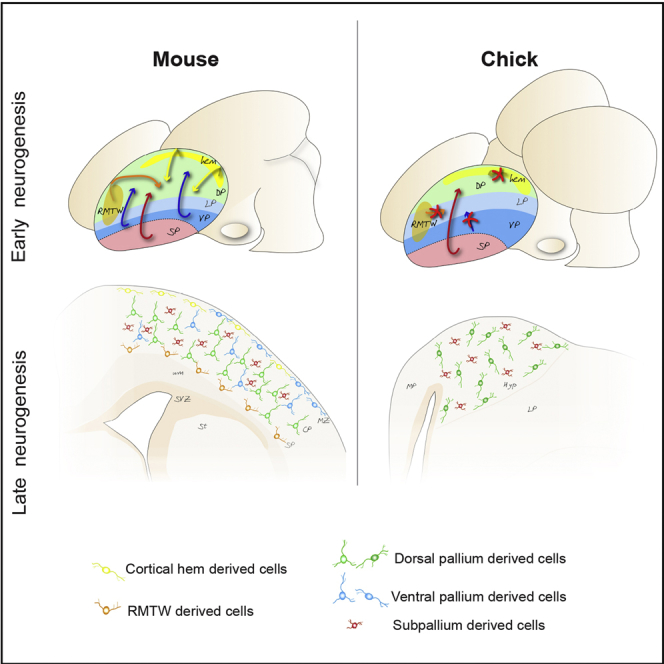

## Teaser

Neocortical formation crucially depends on the early tangential arrival of several transient glutamatergic neuronal populations. García-Moreno et al. find that these neuronal migrations are absent in the developing brain of chicks. The mammalian uniqueness of these developing migrations suggests a crucial role of these cells in the evolutionary origin of the neocortex.

## Introduction

Understanding the functional and structural complexity of the neocortex requires knowledge of its evolutionary origin and embryonic development (Geschwind and Rakic, 2013). Because morphological evolution is underlain by changes in embryo development (Gould, 1977), modifications in the neurogenic programs of neocortical progenitors and alteration of the migratory patterns of derived neurons must have been key in shaping the mammalian brain. Comparative developmental analysis of homologous regions to the neocortex in non-mammalian brains provides insight into the origin of the cerebral cortex.

The mammalian neocortex is derived from the most dorsal region of the embryonic telencephalon, the dorsal pallium (DP) (Puelles, 2011). Most cortical excitatory glutamatergic neurons are born in the germinal zones of the DP and migrate radially toward the pial surface (Rakic, 1971). The vast majority of the inhibitory GABAergic interneurons are generated in the subpallium and migrate tangentially to the cortex (Anderson et al., 1997, de Carlos et al., 1996, Tremblay et al., 2016). In addition, several populations of glutamatergic neurons are generated outside of the neocortical neuroepithelium and travel to the DP through tangential migration (Barber and Pierani, 2016, Marín, 2013). These neurons are largely transient but play essential roles in orchestrating the telencephalic developmental program. Cajal-Retzius (C-R) cells settle early in the most superficial of the cortical layers, the marginal zone (MZ), from where they exert important roles in cortical formation, such as leading the inside-out neurogenic pattern (Beffert et al., 2004, Ogawa et al., 1995) and maintaining radial glial cell function (Supèr et al., 2000). Cajal-Retzius cells reach the neocortex by tangential migration from a variety of telencephalic origins, such as the cortical hem (García-Moreno et al., 2007), the rostral septum, and the ventral pallium (VP) (Bielle et al., 2005). Subplate neurons populate the deepest region of the cortical plate, from where they control the entry of early thalamic fibers into the cortex (Hoerder-Suabedissen and Molnár, 2015). Although their precise origin remains unclear, a subpopulation of subplate neurons characterized by the expression of the *Lpar1* gene is generated outside of the cortical boundaries (García-Moreno et al., 2008, Pedraza et al., 2014), in the rostral and medial region of the telencephalic wall. A population of transient glutamatergic pyramidal neurons also reaches the neocortex by tangential migration derived from the *Dbx1*-expressing region of the VP (Teissier et al., 2010). Ablation of Dbx1-expressing populations reduces cortical neuronal numbers by 20%, and, thus, it is believed that Dbx1-derived neurons with VP origin promote local cortical neurogenesis as they migrate to the cortex (Barber and Pierani, 2016).

Lineage tracing techniques have shown direct evidence for the extra-neocortical origins of the glutamatergic tangential migrations for both Cajal-Retzius cells (García-Moreno et al., 2007, Imayoshi et al., 2008) and subplate neurons (García-Moreno et al., 2008, Pedraza et al., 2014) in the mouse cortex. However, these techniques have not been directly applied to Dbx1-derived transient pyramidal neurons *in vivo*. In addition, despite their importance in mammalian cortical development, very little is known about these transient populations in non-mammalian species (Nomura et al., 2008, Puelles, 2011). Are such tangential contributions to the DP conserved among amniote brains or are they a mammalian novelty? Did their appearance coincide with or lead to the origin of the current six-layered neocortex? What developmentally divergent mechanisms regulate neurogenesis in the DP and VP in amniote brains?

Here we compared the generation and migration of various neuronal populations in mouse and chick. We performed *in ovo* electroporation to trace the complete lineages of chick neural progenitors from defined sectors of the telencephalon, aided by transposase-mediated permanent labeling of the progenitors. We describe that none of these tangentially migrating transient neuronal populations were present during avian dorsal pallial development. In addition, ectopic expression of the mammalian homeobox gene *Dbx1*, a transcription factor expressed in the mammalian but not avian VP, did not promote tangential migration in the chick brain, although it triggered more differentiative and less self-renewing neurogenesis. We propose that these two novelties in mammals, tangential arrival of the transient glutamatergic populations and the decrease of VP neurogenesis caused by *de novo* Dbx1 expression, could have been a major developmental divergence to drive mammalian cortical evolution.

## Results

### Identification of Internal Boundaries of the Developing Chick Telencephalon

**Figure 1:**
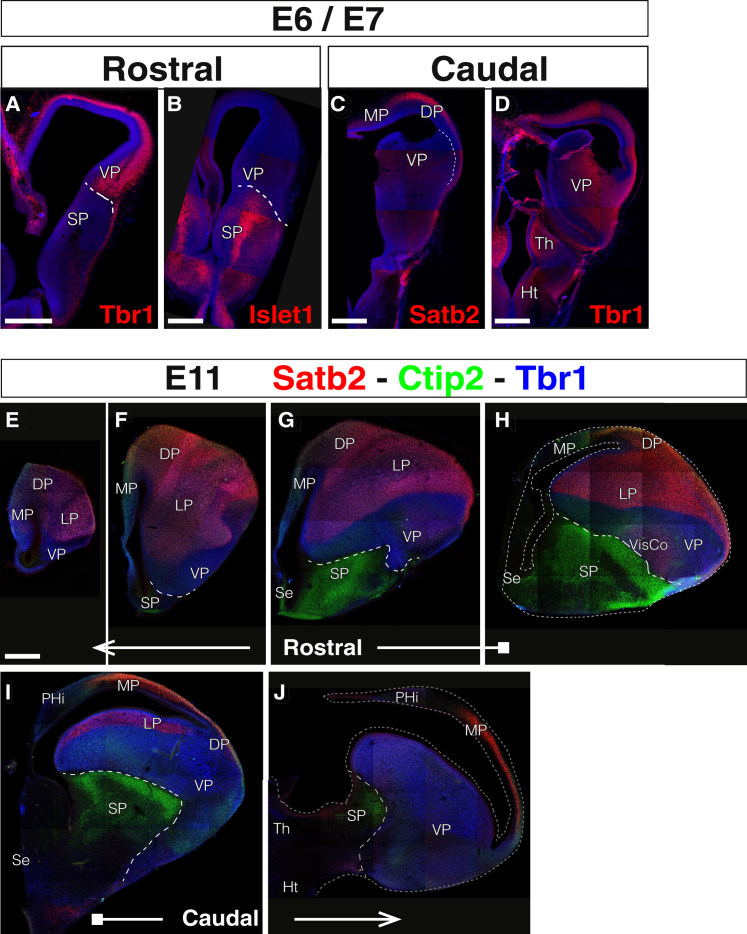
Pallial Subdivisions in the Chick Telencephalon at Early and Late Neurogenesis Shown are coronal sections, medial at the left. (A–D) Immunostainings for pallial (Tbr1, A and D; Satb2, C) and subpallial (Islet1, B) markers on early embryos (E6–E7). DAPI counterstain is shown in in blue. (E–J) Immunostainings of pallial (Tbr1, blue; Satb2, red) and subpallial (Ctip2, green) markers at the end of neurogenesis (E11). The images show different anterior (E and F), middle (G and H), and posterior (I and J) levels of the telencephalon. Dashed lines mark the PSB in (A), (B), and (E)–(J) and the boundary between the LP and VP in (C). Scale bars represent 500 μm; the scale in E applies to (E)–(J).

The avian telencephalon is comprised of a series of nuclear regions that are strikingly different from the predominantly laminated mammalian telencephalon (Jarvis et al., 2013, Reiner et al., 2005). To compare the different origins and fates of neuronal populations in the mature brain, we first established the positions and boundaries of the different pallial sectors in the chick brain after the completion of neurogenesis and most neuronal migrations. We used well-known markers of these sectors to reveal their boundaries, focusing on markers whose expression is constant during the neurogenic period. Tbr1 is a commonly used marker of both mouse and chick pallium (Puelles et al., 2000). In both early (embryonic day [E]6/E7) and late (E11) stages of chick neurogenesis, Tbr1 expression was detected in all pallial areas, defining a clear pallial-subpallial boundary (PSB; dashed lines in Figures 1A and 1D–1J). The brightest Tbr1 expression was found in the deep mesopallium (MsP) and in the nidopallium (NP), which derive from the lateral pallium (LP) and VP, respectively. We used Islet1 (Abellán and Medina, 2009) and Ctip2 (Suzuki et al., 2012) as markers to define the PSB because they are specifically expressed in the subpallial mantle zone (Figures 1B and 1E–1J). In addition, Ctip2 exhibited low expression in other telencephalic areas because it was detected in the parahippocampal area derived from the medial pallium (MP) (Figures 1E–1H). Satb2 (Suzuki and Hirata, 2014, Suzuki et al., 2012) delineated the boundary between the LP and VP (Figure 1C and 1E-J). Satb2 was highly expressed in the LP derivatives and showed nearly no expression in the NP, apart from some faintly bright cells in the visual NP region.

We identified the location of the DP by differential expression of selected markers. Ctip2 was expressed more in the MP than in the DP; Satb2 defined the MP-to-DP limit because it is nearly absent in the rostral MP (Figures 1C and 1E–1J). The ventral border of the DP was identified using Satb2 and Tbr1; the former is expressed in the hyperpallial columns to a variable degree, whereas DP has a low level of Tbr1 expression.

The establishment of these pallial areas was used to define the origin and cellular destination of the different telencephalic populations studied.

### Pallial GABAergic Interneurons Originate in a Restricted and Conserved Subpallial Region

The first populations we investigated were GABAergic interneurons. In mammals, interneurons have subpallial (SP) origins and migrate to the neocortex tangentially (Anderson et al., 1997, de Carlos et al., 1996). Although homologous tangential migration has been described in several vertebrate species (Carrera et al., 2008, Cobos et al., 2001a, Métin et al., 2007, Moreno et al., 2008, Tuorto et al., 2003), the specific telencephalic sector that gives rise to the avian pallial interneuronal population remains undefined (Cobos et al., 2001a, Tuorto et al., 2003).

**Figure 2:**
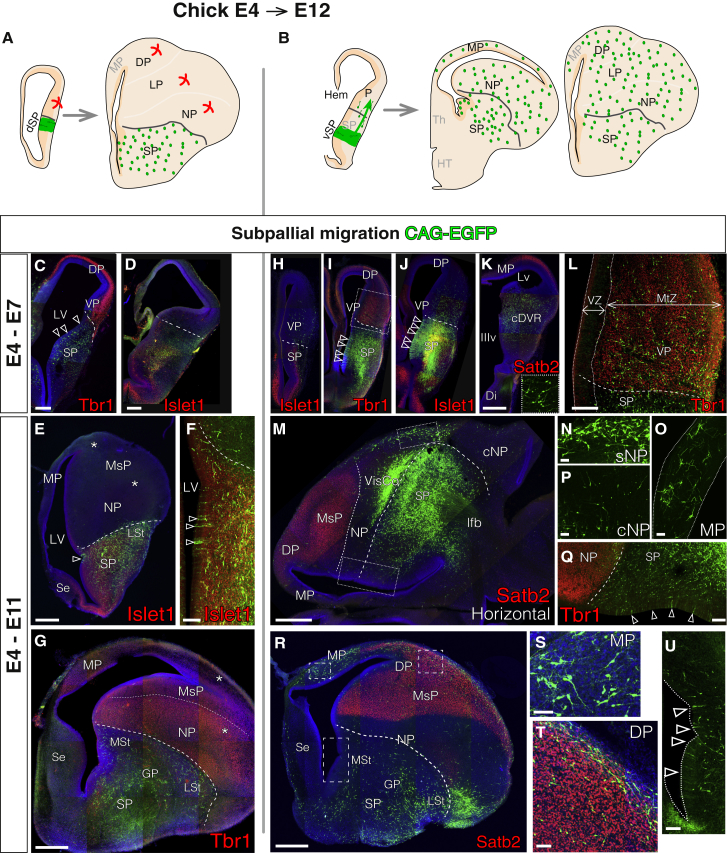
Restricted Subpallial Origin of Chick Pallial Interneurons Shown are coronal sections, medial at the left. (A and B) Schematic diagrams depicting the lineage of dorsal (dSP, A) and ventral (vSP, B) SP progenitors. (C and D) The short-term lineage of dSPs (green) does not cross the PSB at E7, delineated by Tbr1 (C) and Islet1 (D) immunostaining (red, n = 3). (E–G) dSP progeny remains within SP boundaries at the end of neurogenesis (E11, n = 4). Neurons do not enter the pallium (asterisks). Section in (E) is more anterior to (G). The electroporated SP area lies just ventral to the VP, as shown in (F). (H–K) Rostral-to-caudal examples of the short-term tracing from vSPs (rostral, H; middle, I and J; caudal, K). GFP cells migrated tangentially in the rostro-caudal and ventro-dorsal axes, crossing the PSB (n = 4). (L) Power view of the rectangle depicted in (I), showing that vSP cells migrate through the entire pallial mantle zone (MtZ) depth marked with Tbr1. (M–U) Long-term tracing colonized the whole pallium at E11 (n = 6). Horizontal (M–Q, medial at the bottom, rostral at the left) or coronal (R–U) sections show that GFP cells entered the pallium, including the DP, superficial NP (N), medial pallium (O), and caudal NP (P). (N)–(Q) show power views of the rectangles shown in (M). (T) and (U) show power views of the rectangles shown in (R). Dashed lines mark the PSB. The dotted lines in (M) define the boundary between the LP and VP. Empty arrowheads point to transfected SP progenitors. DAPI counterstain is shown in blue. Scale bars represent 500 μm in (E), (G), (M), (V), and (K) for (H)–(K); 250 μm in (D); 100 μm in (F), (L), (Q), and (U); and 50 μm in (N), (O), (S), and (T). See also Figures S1 and S2.

We transfected chick telencephalic progenitors on E4, the earliest telencephalic neurogenesis in chick, by permanent *in vivo* lineage tracing (Supplemental Experimental Procedures), which enables the identification of the electroporated progenitor region. When we labeled chick subpallial progenitors, we found two different cell lineages (Figures 2 and S1). First, the rostral and dorsal regions of the SP (striatal SP), adjacent to the ventral border of the VP, produced an exclusively radially migrating progeny (Figure 2A) because short-term lineage tracing showed that subpallial cells did not cross the PSB 2 or 3 days after electroporation (Figures 2C and 2D). Later, cells that originated at the SP territory settled radially, in the striatal Islet1+/Tbr1− areas (E11; Figures 2E–2G). Derived cells settled from the lateral striatum, near the pial surface, to the deepest nuclear regions of the medial striatum. Strikingly, cells remained in the SP and did not cross the PSB.

Electroporations at more posterior and ventral subpallial sectors (pallidal SP) showed the expected tangential waves of interneuron migration (Figures 2B and 2H–2T). On E7, 3 days after electroporation, large cohorts of neurons migrated tangentially, crossed the PSB, and invaded both the adjacent VP and LP, reaching the distant DP in some cases (Figures 2H–2K). Neurons did not restrict their movement to a particular stratum of the pallial mantle zone because they migrated through and settled along the entire depth of the pallium (Figure 2L). This migration followed a rostro-caudal pattern; cells traveled dorsally from the caudal regions of the SP to more anterior and posterior regions of the pallium (Figures 2H and 2K). Long-term tracing experiments, aided by transposase-mediated permanent labeling, showed a large number of neurons occupying the entire pallial extension, spanning from the NP to the parahippocampal area (MP-derived). These neurons were found to be negative for glutamatergic markers Satb2 and Tbr1, which confirmed their GABAergic nature (Figure S2). Many cells were also found to remain in the SP (mainly the pallidal Islet1− region; Figures 2B, 2M–2T and S2).

Taken together, these experiments revealed that tangentially migrating interneurons originate from a specific limited region of the chick SP. In addition, these experiments confirmed that the permanent staining by PB transposition (García-Moreno et al., 2014) is an efficient and sensitive method for labeling tangential migrations and identifying singular progenitor niches.

### Lack of Dorsal Tangential Migration from the Rostral Medial Telencephalic Wall in the Chick Brain

**Figure 3:**
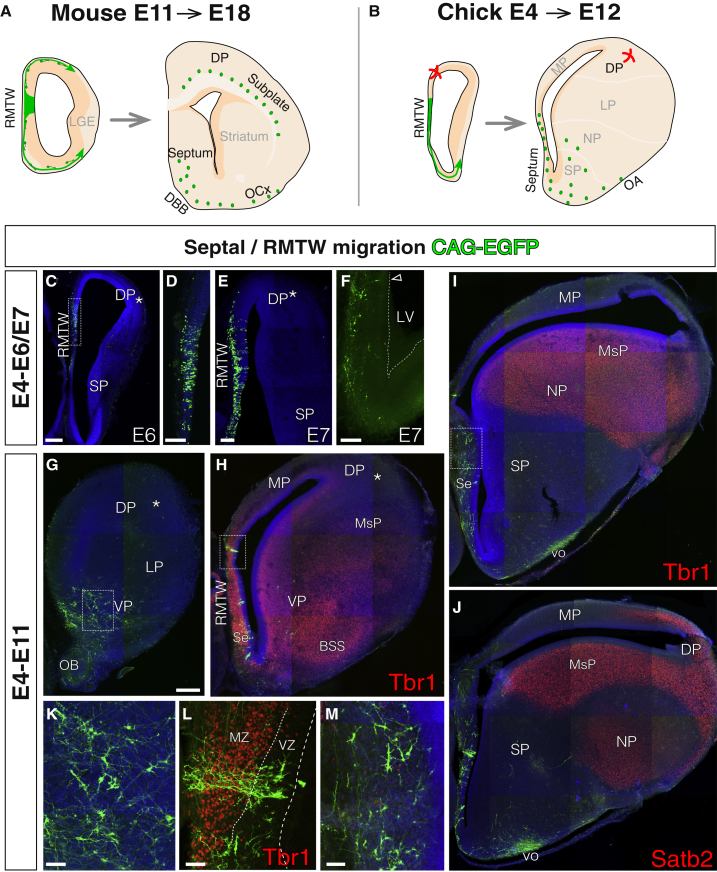
Only Ventral but No Dorsal Migration from the Avian Rostral Medial Telencephalic Wall Shown are coronal sections, medial at the left. (A and B) Schematic diagrams summarizing the lineage of RMTW progenitors in mouse (A) and chick (B). (C–F) Rostral sections from two different animals (C and E) and power magnifications (D) of the telencephalon, showing that GFP-labeled cells remain locally after short-term tracing (n = 3) and do not migrate dorsally toward the DP region (marked with an asterisk) but ventrally toward septal areas (F). (G–J) Rostral-to-caudal examples of long-term tracing from the RMTW supported by immunostaining for the pallial markers Tbr1 and Satb2 (n = 3). Labeled progenitors are visible in the RMTW in (H); the progeny migrates ventrally, reaching rostral (G) and caudal regions (I and J) of the septal and olfactory systems. No cells were found to migrate dorsally toward either the lateral regions of MP or DP (asterisks). Axons from the olfactory bulb cells were visible, traveling caudally by the ventral olfactory tract (vo). (K–M) Power magnifications of the rectangles (K, L, and M for rectangles in G, H, and I, respectively), showing the morphology of labeled neurons. The progenitors in (M) are pallial, as they lie down the Tbr1+ postmitotic region of the RMTW. DAPI counterstain is shown in blue. Scale bars represent 250 μm in (C) and (G) for (G)–(I); 100 μm in (D) and (E); and 50 μm in (K)–(M).

Subplate neurons are a heterogeneous cell population and are generated in diverse neurogenic niches (Hoerder-Suabedissen and Molnár, 2015). One of these niches is the murine rostral medial telencephalic wall (RMTW). This sector gives rise to a population of tangentially migrating Lpar1-expressing glutamatergic neurons that populate the subplate layer (Pedraza et al., 2014; Figure 3A). We investigated the presence of this particular subset of DP-colonizing migratory cells in the chick brain. Our electroporations covered the entire RMTW, which comprises germinative zones in the pallium (MP) and SP (rostral septum) (Figure 3).

Short-term lineage tracing (E4 to E7) from the RMTW revealed two different migratory behaviors of the derived cells. The majority of newly generated neurons migrated radially to the surface of the pallial and subpallial regions of the RMTW (Figures 3C–3F). Additionally, a stream of cells migrated ventrally toward the septal surface (Figure 3F), similarly to migrations described in the mouse (García-Moreno et al., 2008). However, no dorsal trajectory was found in the chick. Similarly divergent migrations were observed in long-term experiments, in which the transposase guarantees the labeling of the entire progeny from E4 to E11 (Figures 3G–3M). Germinative zones of the RMTW contributed only to the overlying mantle zone of the rostral MP and septal area. More rostrally, the RMTW cells settled to the anterior olfactory region above the olfactory bulb in the VP, alongside the olfactory bulb itself (Figures 3G and 3M). However, RMTW-derived cells in the chick never invaded the distant DP (asterisks in Figure 3). The stream of cells migrating ventrally came to populate the surface of the septum, diagonal band, lateral striatum, and superficial olfactory areas next to the ventral olfactory tract (Figures 3I and 3J). These experiments suggest that subplate-like cells derived from the RMTW do not contribute to the avian DP (Figure 3B).

### Absence of Tangential Migration of Cajal-Retzius-like Neurons in the Chick Telencephalon

**Figure 4:**
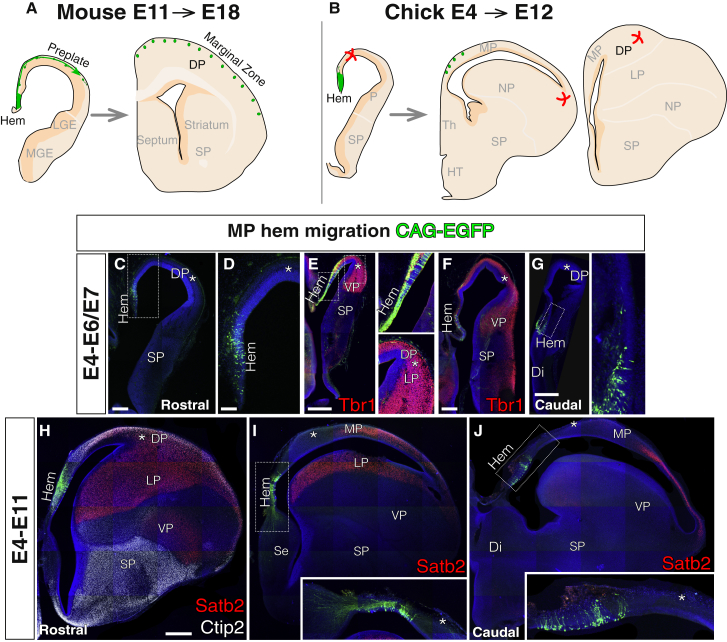
Lack of Tangential Migration from the Avian Cortical Hem Coronal sections are shown, medial at the left. (A and B) Schematic diagrams depicting the lineage of cortical hem progenitors in mouse (A) and chick (B). (C–G) Rostral-to-caudal series of sections (rostral, C; middle, E; caudal, F and G) of early embryos electroporated at the cortical hem (n = 3) and its power magnification (D for C). GFP cells did not migrate dorsally toward the DP (asterisk) at E6/E7. Even when other MP progenitors next to the cortical hem were transfected (E and F), the lineage remained locally. Tbr1 immunostaining supported regional identification. (H–J) Examples of telencephalic sections after long-term tracing from the cortical hem (E11, n = 3), immunostained for Satb2 (red) and Ctip2 in (H) (white). At the end of neurogenesis, cortical hem progenitors were still visible and displayed a radial morphology (see insets in I and J). Cortical hem derivatives did not migrate through the MP toward the DP and settled locally in the most ventral part of the MP. DAPI counterstain is shown in blue. Scale bars represent 500 μm in (E), (G), and (H) for (H)–(J); 250 μm in (C) and (F); and 100 μm in (D). See also Figure S3.

In the mammalian brain, several pallial germinative zones generate Cajal-Retzius cells (Barber and Pierani, 2016). We searched for these migratory cells in the avian DP, focusing first on the medial source of Cajal-Retzius cells, the cortical hem (Figure 4). This structure appears from medial to caudal levels of the MP, at its most distant and ventral region (Grove et al., 1998, Abellán et al., 2014), and, in the mouse, it produces migratory cells that settle early in the DP (Figure 4A). Short-term electroporations of the presumptive chick cortical hem on E4 revealed the absence of a tangentially migrating neuronal lineage during early telencephalic neurogenesis (Figures 4C–4G). Because of the reduced size of the cortical hem, electroporations occasionally extended to other, unintended MP progenitors. However, neither cortical hem nor MP progenitors produced tangentially migrating cells. The lineage of these progenitors only occupied MP areas and never reached the distant DP regions (Figures 4C–4G). Furthermore, when the long-term lineage was analyzed at the end of neurogenesis (E11), no MP-derived neurons were found in the DP (Figures 4H–4J and S3). The transposase-mediated labeling confirmed that cortical hem neurons generated from E4 to E11 did not migrate to the DP. Therefore, these progeny-tracing experiments suggest that the avian cortical hem does not produce tangentially migrating Cajal-Retzius-like cells (Figure 4B).

**Figure 5:**
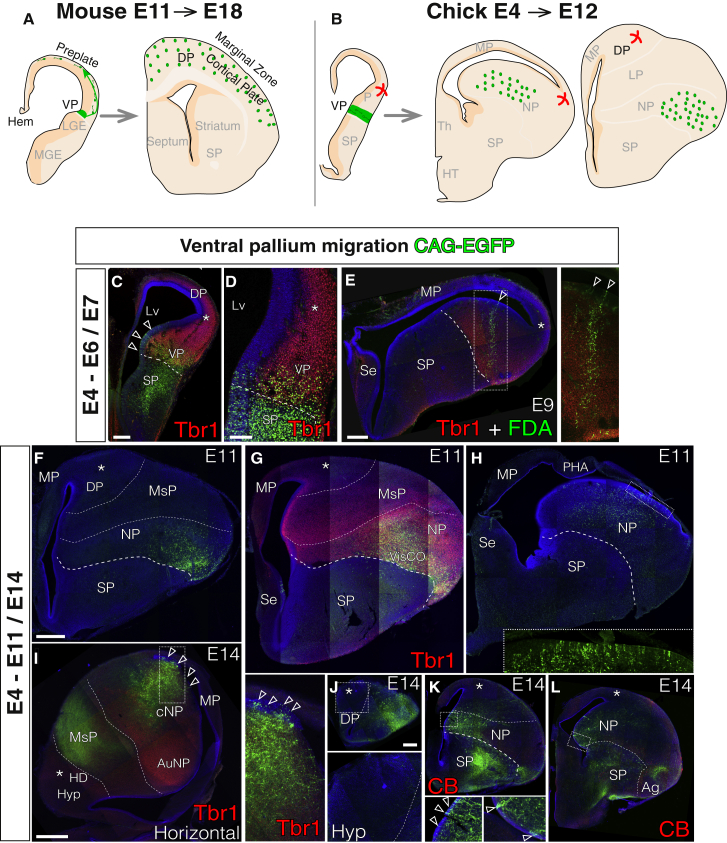
Exclusively Radial Derivatives from the Chick VP Coronal sections are shown (medial at the left), except in (I), which is a horizontal section (medial at the bottom, rostral at the left). (A and B) Schematic diagrams depicting the lineage of VP progenitors in mouse (A) and chick (B). (C and D) Short-term electroporations at the VP produced a progeny that only settled radially within VP limits (E6/E7, n = 6). (D) Power magnification of another specimen (C). Pallial Tbr1 immunostaining confirmed the VP regional identity of the labeled progenitors. No tangential migration toward dorsal areas of the pallium (asterisk) was found. (E) Columnar radial derivatives within the VP 5 days after FDA injection in the VP GZ (E9, n = 2). The rectangle is magnified in the right inset. Tbr1 immunostaining showed that the traced neuronal column belongs to the VP. (F–L) Series of sections representative of three independent long-term lineage studies (F–H; I and J–L show calbindin and Tbr1 immunostaining; analysis based on n = 5). Derived cells at E11 were confined to the VP derivatives (NP). VP-labeled progenitors occupied caudal positions to the superficial lineage traced. The membrane-tagged EGFP reporter was used in (I)–(L), showing major VP projections to either the MsP (I) or through the SP toward the diencephalon (ventral amygdalofugal pathway and lateral forebrain bundle, K and L). The insets in (H), (I), (K), and (L) show the presence of labeled progenitors at the VP. The inset in (J) shows the lack of labeled cells in the rostral DP. Thick dashed lines represent the PSB. Thin dashed lines establish the boundaries of the MsP. Empty arrowheads point to VP progenitors. DAPI counterstain is shown in blue. Scale bars represent 1 mm in (I); 500 μm in (F) for (F)–(I) and in (J) for (J)–(L); 250 μm in (C) and (E); and 100 μm in (D). See also Figure S4.

Early murine Dbx1+VP progenitors give rise to tangentially migrating Cajal-Retzius cells that populate the neocortex (Figure 5A). Electroporations of the early chick VP labeled a very large radially migrating progeny toward the dorsal ventricular ridge (DVR) mantle zone (Figure 5B). In short-term tracing experiments, no tangentially migrating cells were found to be directed toward more dorsal regions of the pallium (Figures 5C and 5D). This lack of VP-derived tangential migration was consistent from anterior to posterior regions of the telencephalon. The focus of our electroporation ranged from very extensive, covering the entire ventral and lateral pallial or subpallial ventricular regions, to a much smaller and precise sector of VP progenitors. We also performed experiments targeting a smaller but more precise labeling of progenitors using injections of the cell tracker (García-Moreno et al., 2008) fluorescein dextran amine (FDA) in E4 embryos. Cells generated in the small and circumscribed injection site in the VP migrated only radially, and at E9, they occupied the whole depth of the VP (Figure 5E).

Together with the absence of cortical hem-derived tangential migration (Figure 4), this set of experiments in the VP indicates that the avian DP does not receive a population homologous to the tangentially migrating Cajal-Retzius cells in mammals.

### The Avian VP Does Not Provide Neurons for the DP

Observations in the Dbx1-LacZ mouse model suggest that, at mid-stages of neurogenesis, Dbx1+VP progeny migrate tangentially to the cortical plate, where they settle to differentiate into a transient glutamatergic pyramidal neuron population (Teissier et al., 2010). We investigated the presence of such tangential migration in the avian brain using long-term permanent tracing. If avian VP progenitors generate a tangentially migrating neuronal population, then our permanent transposase-driven labeling should identify such neurons derived from this germinative region in the chick brain. However, our long-term experiments, in which the transposase guarantees the labeling of the entire progeny from E4 to E11, revealed only radial and not tangential migrations from the VP during mid- and late-neurogenesis in the chick (Figures 5F–5L and S4). We tested this across different rostro-caudal levels of the chick VP, and the lineages detected were invariably radial, extending from caudal-deep regions to rostral-superficial regions of the NP. VP-derived neurons settled at rostral levels in the olfactory, visual, and somatosensory NP areas (Figures 5F and 5G), in the auditory NP, and amygdalar nuclei at the caudal DVR (Figures 5H, 5I, and 5L). With the rare exception of a few neurons in the ventral mesopallium (Figure 5F), no cells were found in the hyperpallium or other DP-derived regions (asterisks in Figure 5).

Similar to RMTW-derived subplate cells (Figure 3) and Cajal-Retzius cells (Figures 4 and 5), no glutamatergic VP-derived neurons were found in the DP of developing chick brains.

### Ectopic Expression of Mammalian *Dbx1* in the Chick VP Modifies Neurogenesis

A crucial difference between the VP germinative zones (GZs) in mammals and avian brains is the expression of DBX1. Unlike in mammals, avian VP progenitors do not express DBX1 (Bielle et al., 2005). We hypothesized that recruitment of DBX1 expression in the ancestral mammalian VP progenitors may have been partly responsible for the divergence of developmental programs during evolution, leading to reduced neurogenesis in VP and tangential migration of VP-derived neurons in mammals.

**Figure 6:**
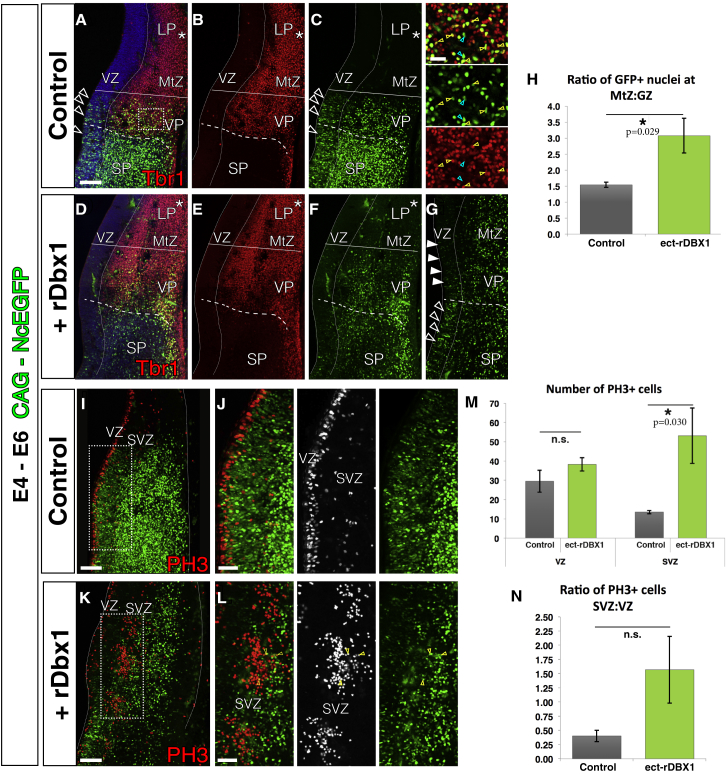
Ectopic rDbx1 Expression Modifies VP Neurogenesis but Does Not Induce Tangential Migration in the Chick Brain Coronal sections are shown, medial at the left. (A–F) Short-term ectopic rDbx1 expression electroporations of VP progenitors (D–F) produced, at E6, a radial progeny of VP-derived cells (n = 5), similar to control experiments (A–C, n = 4). No cells were found to migrate dorsally toward the LP or DP (asterisks). The Tbr1 pallial marker is shown in red; the inset in (C) shows many nuclear GFP-labeled cells derived from the VP that express Tbr1 (yellow arrowheads), whereas a minority derived from the subpallium do not express Tbr1 (cyan arrowheads). (G) Two days after ect-rDBX1, VP GZ GFP expression was strongly diminished (arrowheads), whereas GFP was still clearly visible in SP electroporated progenitors (empty arrowheads). (H) Ratio of GFP+ cells in the MtZ relative to the GZ (MtZ:GZ; 1,803 GFP+ ect-rDBX1 cells counted in n = 5; 1,113 GFP+ control cells counted in n = 4). (I–L) Short-term ect-rDBX1 (K and L) promoted an increase in SVZ divisions with respect to the control experiment (I and J; control, n = 4, 588 PH3+ cells counted; ect-rDBX1, n = 5, 883 PH3+ cells counted). Mitotic nuclei were detected by immunohistochemistry for PH3 (in red, except in the center of J and L, in white). (J) and (L) are high power views of the rectangles shown in (I) and (K), respectively. Mitotic nuclei are arranged in clusters, but few mitotic cells derived from electroporated ect-rDBX1 electroporated progenitors (yellow arrowheads). (M) Number of mitotic pH3+ cells in the GZs of the VP in control and ect-rDBX1 animals. (N) Ratio of pH3+ cells in the subventricular zone relative to the ventricular zone (SVZ:VZ) in control and ect-rDBX1 experiments. DAPI counterstain is shown in blue. Scale bars represent 100 μm in (I), (K), and (A) for (A)–(G); 50 μm in (J) and (L); and 25 μm in (A) in the inset. Error bars correspond to SD. See also Figure S5.

To test this hypothesis, we performed ectopic expression of DBX1 (ect-rDBX1) in E4 chick VP by electroporating a nuclearly tagged EGFP plasmid together with the pCAX-rDbx1 construct or without it (a plasmid that expresses the rat *Dbx1* gene; ectopic or control experiment, respectively). Embryos were harvested on E6. The results revealed that ect-rDBX1 did not modify the migratory behavior of VP-derived cells (Figures 6 and S4). The short-term lineage experiment showed migrations in early embryos to be exclusively radial (Figures 6A–6G; as described in Figures 5C–5E). No changes in migration or neuronal morphology were detected after ect-rDBX1.

We also analyzed the neurogenic properties of ect-rDBX1 progenitors by comparing the proportion of neurons derived from each VP progenitor. Given that the boundary between the VP and LP at these early stages is not easily distinguishable (Figure 1), we defined VP as the region 300 μm dorsal to the PSB. Because a variable number of SP progenitors can also be labeled in most experiments because of the very small territory of the VP at E4, all EGFP+/Tbr1− cells in the mantle zone (MtZ) were subtracted to account for the migration of SP-derived interneurons. In ect-rDBX1 chicks, the number of neurons derived per VP progenitor doubled, defined as the ratio of EGFP+ cells in the MtZ versus the GZs (Figure 6H; control, 1.53 ± 0.18; ect-rDBX1, 3.07 ± 1.08; mean ± SD, p = 0.029). In contrast to control animals, in the majority of ect-rDBX1 electroporated animals, the VP progenitor pool was depleted 2 days post-electroporation (Figure 6G). Together, these data suggest that ect-rDBX1 induced terminal differentiation, leading to premature depletion of the VP progenitor pool after generating new neurons or transient SVZ progenitors.

In addition, the number of mitotic (PH3-immunoreactive) nuclei in the chick SVZ was four times higher after ect-rDBX1 expression compared with the control (Figures 6M and 6N; control, 13.53 ± 1.98; ect-rDBX1, 53.16 ± 28.72; p = 0.030), whereas no differences were observed in the VZ. Furthermore, these mitotic nuclei appeared to cluster and form groups of 30–80 cells, which were not seen in control chick brains (Figures 6I–6L). Almost no cells in these clusters were EGFP+ (Figure 6L). This may indicate that the mechanism promoting SVZ proliferation was non-cell-autonomous.

These experiments showed that, although it did not confer a tangential phenotype to the neuronal lineage, ectopic expression of rDBX1 in the chick VP altered neurogenesis in two ways. First, chick VP divisions were more differentiative and less self-renewing in ect-rDBX1animals. And second, proliferation in SVZ increased, and this may be due to an additional non-cell-autonomous effect.

### Potential Regulatory Differences behind Distinct Behaviors in Avian and Mammalian VPs

**Figure 7:**
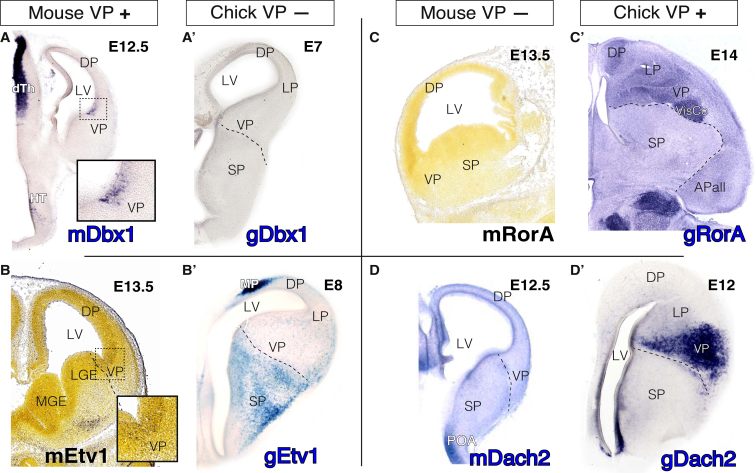
Genes Divergently Expressed in the VP of Mouse and Chick Coronal sections are shown, medial at the left, except for (C), which shows a sagittal section, rostral at the left, and (C’), which shows a horizontal section, rostral at the top. (A–D’) Expression data from *in situ* hybridization on mouse (A–D) and chick (A’–D’) embryonic specimens at neurogenic stages as detailed in the figure. (A–B’) Dbx1 and Etv1 are expressed in the early murine VP but not in the chick homologous region. (A) Mouse Dbx1. (A’) Chick Dbx1. (B) Mouse Etv1. (B’) Chick Etv1. (C–D’) RorA and Dach2 show an opposite expression pattern and are expressed in the chick VP but not in the mouse. (C) Mouse RorA. (C’) Chick RorA. (D) Mouse Dach2. (D’) Chick Dach2. See also Figures S5–S7.

In search of the mechanisms driving the divergent migration of VP neurons, we aimed to find transcription factor genes whose expression differs qualitatively in the developing VP of both species (Figure 7). Despite high a similarity in the expression patterns of transcription factors in early telencephalic progenitors, we could identify 4 genes that display divergent expression patterns: *Dbx1* and *Etv1* were found to be expressed in the murine VP but not in the chick VP (Figures 7A–7B’), whereas *RorA* and *Dach2* showed the opposite expression pattern in the two species (Figure 7C–7D’). We suggest that the mammalian novel expression of any of these genes, or a combination of them, could be responsible for the different migratory behavior of VP cells. To get further insights into the potential regulatory differences leading to these distinct behaviors, we next analyzed the regulatory landscapes of two genes that are expressed only in the mammalian VP to identify putative mammal-specific enhancers driving this expression. We used chromatin accessibility data (assay for transposase-accessible chromatin using sequencing [ATAC-seq]) generated by the Encyclopedia of DNA Elements (ENCODE) project from forebrain at E11.5, E12.5, and E14.5 to define active enhancers at these stages and searched for global conservation patterns using multi-vertebrate chain genome alignments. Most enhancers in the large potential regulatory landscapes of *Dbx1* and *Etv1* (155 Kbp and 1.7 Mbp, respectively) were detected in all studied jawed vertebrates (Figures S5 and S6), including a proximal regulatory region ∼3.7 Kbp upstream of *Dbx1* that was previously shown to drive expression to the forebrain (Lu et al., 1996; Figure S5, blue box). However, we also observed several distal mammal-specific ATAC-seq peaks associated with both loci (Figures S5 and S6, red boxes), including a mammal-specific region linked to a vertebrate-specific highly conserved non-coding region in *Dbx1* (Figure S5, orange box). Finally, we also investigated the qualitative genomic conservation of two VISTA enhancers (hs636 and hs876), which have been reported to drive expression in the mouse VP (Pattabiraman et al., 2014). Interestingly, as in the case of the proximal *Dbx1* enhancer, these enhancers were found in all jawed vertebrates (Figure S7), suggesting that functional differences may be due to specific nucleotide substitutions that occurred in the mammalian lineage.

## Discussion

Several populations of cortical glutamatergic neurons critical for neocortical development are generated outside of the cortical neuroepithelium of the DP. We show here that none of these external populations migrate to the DP in the avian brain: Cajal-Retzius cells, subplate neurons, and transient pyramidal neurons. The novel arrival of these external cells may have been crucial in early cortical evolution because it could have influenced the developmental program of the mammalian DP at the level of neurogenesis, layering, and circuit formation.

### Conserved Migration of Pallial Interneurons

The subpallial origin of pallial GABAergic interneurons has been extensively studied, and its homology has been proven in all extant vertebrates studied so far (Cobos et al., 2001a, Tuorto et al., 2003, Métin et al., 2007, Carrera et al., 2008, Moreno et al., 2008). However, most of the data have been limited to studies based on the expression of related transcription factors and gamma-aminobutyric acid (GABA). This approach fails to precisely identify the origin and progression of the relevant cell populations, which can only be investigated with cell lineage tracing methods. In chick, some pioneering studies were based on quail-to-chick heterotopic graft transplants (Cobos et al., 2001b) which, because they were performed very early in the embryonic period, could not discriminate the precise subpallial region of origin. Another study used *in vitro* slice cultures (Tuorto et al., 2003), but these offer restricted migratory alternatives to newborn neurons. Although the slice culture experiments pointed to a dorsal region of the subpallium as the origin of interneurons, our *in vivo* whole-brain tracing analysis proved that the ventral subpallium is the major source. Our full lineage tracing demonstrated that subpallial migration in the chick is homologous to the one observed in mammals because the same subpallial subsectors (pallidal) were found to be the main source of migratory interneurons in both taxa (Batista-Brito and Fishell, 2009). This part of the study also validated the experimental approach we used for other tangentially migrating glutamatergic neuronal populations.

### The Chick DP Lacks a Homologous Tangentially Migrating Population of Preplate Neurons

In the early stages of cortical development, Cajal-Retzius cells and subplate neurons travel to the DP preplate (Aboitiz et al., 2005) from extra-neocortical sources before the genesis of the rest of cortical neurons (Barber and Pierani, 2016, García-Moreno et al., 2007, Pedraza et al., 2014). These early cell groups are also inherently transient because they die during early postnatal life after completion of their roles in cortical development. We showed here that none of these neurons migrate to the chick DP. To understand more precisely the evolutionary origin of these migrations, we need to study homologous populations in other outgroups, such as reptiles. Considering that the telencephalic structure of reptiles is less complex than that of both mammals and birds, we hypothesize that the reptilian brain also lacks a homolog of the preplate glutamatergic tangential migration. If this is the case, then it would indicate that the pallio-pallial tangential glutamatergic migrations observed in mammalian brains are a mammalian innovation, whereas the GABAergic tangential migrations are highly conserved (Métin et al., 2007).

It has been suggested, based on molecular profiling, that homolog populations of Cajal-Retzius cells and subplate cells exist in avian brains (Bernier et al., 2000, Wang et al., 2011). However, in light of our findings, the developmental origins of these cells were not conserved. Regarding Cajal-Retzius cell migration in sauropsids, a previous study investigated the developmental origin of avian reelin-expressing pallial neurons (Nomura et al., 2008). Although the authors claim that the quail cortical hem gives rise to tangentially migrating Cajal-Retzius cells, the broad tracing performed could not resolve the precise origin of the migratory population described (see their Figure 2). Alternatively, it is possible that those EGFP+ cells in quail experiments may migrate ventrally from the DP. This is a behavior we have also observed in our experiments, and it is in accordance with what is described in the mouse (García-Moreno et al., 2008). In addition, another study using chick pallial lineage tracing experiments showed results identical to ours (see Figure S2 in Suzuki et al., 2012): a lack of tangential migration from pallial sources.

### The Evolutionary Role of Dbx1: Shaping the Ventral and Dorsal Pallia

Our data show that avian VP progenitors differ widely in their neurogenic behavior from their mammalian homologs. Avian VP progenitors produce a relatively larger lineage, as made clear when comparing the avian DVR with the mammalian piriform cortex and pallial portion of the amygdala (Puelles et al., 2016). Furthermore, avian VP progenitors did not produce cells contributing to the DP. Our data point to a partial function for Dbx1 in the generation of this VP divergence. Ectopic expression of rDbx1 in chick VP progenitors led to more differentiative mitoses, reducing the VZ progenitor pool over time, and increased proliferation in the SVZ because of a non-cell-autonomous effect, which must be transient because SVZ progenitors can only divide a limited number of times (Noctor et al., 2004). Together, these two changes, triggered by expression of Dbx1, led to a reduction of the VP progeny. Altogether, it is likely that *de novo* expression of Dbx1 in mammalian VP progenitors elicited a reduction in VP size, which could have allowed for the relative expansion of the DP.

We show here a crucial migratory divergence in amniotes species. However, this is not driven by the differential expression of Dbx1, as we hypothesized. We found only a handful of transcription factor genes that are differentially expressed in the early pallium of amniotes. Our comparative study of potential regulatory regions further identified several mammal-specific regions associated with *Dbx1* and *Etv1*, which could be responsible, at least in part, for their novel expression in the mammalian VP. However, several other previously characterized enhancers that drive expression in the mouse VP (including those in Dbx1 and VISTA hs636 and hs876 elements) are present in all vertebrates. Enhancer reporter assays of the mammalian and chicken sequences in both systems may shed light onto whether the differences in tangential migration of VP neuroblasts are due to nucleotide substitutions in one lineage (*cis* changes) or to distinct regulatory states in the mouse and chick VP (*trans* changes) (Davidson, 2006).

### Tangential Migration as a Source of Evolutionary Divergence

Tangentially migrating cells are generated in distant GZs with different transcriptional control. Therefore, the morphological, hodological, and neurochemical features of the cells arriving through tangential migration differ from those of the locally born cells. Tangentially migrating glutamatergic cells orchestrate the early circuit assembly at their fate region, as GABAergic interneurons do in the neocortex. Therefore, tangential migration undoubtedly increases the cell diversity, circuit complexity, and computational capabilities of any given brain area. If there is an evolutionary divergence on neuronal tangential migration, then it could be translated into a selective advantage.

In the case of transient cortical populations, the divergence created by tangential migration is not restricted to the complex elaboration of circuitry. These cells also play critical roles during cortical development (Puelles, 2011). The neurogenesis-related functions of Cajal-Retzius cells (Ogawa et al., 1995, Supèr et al., 2000) and the control of early circuitry by subplate cells (Hoerder-Suabedissen and Molnár, 2015) are essential platforms for the subsequent developmental program of the mammalian DP. Without these transient cell groups, the mammalian neocortex would be structured very differently. Therefore, we can speculate that the novel arrival of these cells triggered a divergent plan of DP development. This divergence may have sculpted the mammalian DP by promoting its layering, increased production of neurons, and columnar circuit assembly. The development of all known mammalian neocortical circuitry is centered on subplate and Cajal-Retzius neurons. Such relevant divergences lead us to suggest that novel external pallial contributions to the ancient mammalian DP may have been a key factor in the origin of the current mammalian neocortex.

## Experimental Procedures

### Animals

All animal experiments were approved by a local ethical review committee and conducted in accordance with personal and project licenses under the UK Animals (Scientific Procedures) Act (1986) and in compliance with the current normative standards of the European Union (Directive 2010/63/EU) and the Spanish Government (Royal Decrees 1201/2005 and 53/2013, Law 32/107). Details regarding experimental chick and mice can be found in the Supplemental Experimental Procedures.

### *In Ovo* Electroporation and Tracing

Electroporation of chick embryos was performed as described previously (García-Moreno et al., 2014). For targeted electroporations of focal telencephalic areas, the positive pole was placed next to the telencephalic areas to be electroporated, whereas the negative electrode was positioned at the opposite brain site. Here we exploit the *piggybac* (PB) transposase system (Ding et al., 2005), which delivers transgenes into the genome of the transfected cells. PB turns on the expression of the reporter gene permanently both in the electroporated stem cells and their whole progeny (García-Moreno et al., 2014). In this work, we electroporated chick embryos *in ovo* at the onset of telencephalic neurogenesis on E4, Hamburger and Hamilton (HH) stage 23–24 (Hamburger and Hamilton, 1951). Details regarding *in ovo* electroporation can be found in the Supplemental Experimental Procedures.

In other experiments, FDA (molecular weight [MW], 3,000) was injected into the embryonic neuroepithelium (E4). This way we obtained more precise, although not permanent, labeling of progenitors (García-Moreno et al., 2008, Métin et al., 2007).

### *In Utero* Electroporation

Transfection by electroporation of embryonic neural progenitors was performed as described previously (García-Moreno et al., 2010).

### Plasmids

Most of the plasmid constructs employed in this study were also employed in a previous study (García-Moreno and Molnár, 2015), which describes these in detail. Details regarding the plasmids employed can be found in the Supplemental Experimental Procedures.

### Tissue Processing and Immunohistochemistry

Mice and chick embryo fixation and perfusion, tissue processing, and immunohistochemical analysis were performed as described previously (García-Moreno and Molnár, 2015); details can be found in the Supplemental Experimental Procedures.

## Supplemental Information

Document S1. Supplemental Experimental Procedures and Figures S1–S7

Document S2. Article plus Supplemental Information
