# Supplementary material for: Absence of Tangentially Migrating Glutamatergic Neurons in the Developing Avian Brain
Source: Cell Rep. 2018 Jan 2;22(1):96–109. doi: 10.1016/j.celrep.2017.12.032 (PMC5770341; doi:10.1016/j.celrep.2017.12.032)
Supplement: Document S1. Supplemental Experimental Procedures and Figures S1–S7 [file mmc1.pdf]

Cell Reports, Volume *22*

Supplemental Information

Absence of Tangentially Migrating Glutamatergic Neurons in the Developing Avian Brain

Fernando García-Moreno, Edward Anderton, Marta Jankowska, Jo Begbie, Juan Manuel Encinas, Manuel Irimia, and Zoltán Molnár

Supplementary Experimental Procedures

Animals

All animal experiments were approved by a local ethical review committee and conducted in accordance with personal and project licenses under the UK Animals (Scientific Procedures) Act (1986), and were conducted in compliance with the current normative standards of the European Union (Directive 2010/63/EU) and the Spanish Government (Royal Decree 1201/2005 and 53/2013; Law 32/107). Fertilized hen eggs, obtained from Winter Egg Farm (UK), were incubated at 38 °C in humidified atmosphere until required stages (Haumburger and Hamilton, 1951). The day when eggs were incubated was considered E0. Adult C57BL/6 mice were obtained from a local breeding colony at the University of Oxford (based on the Harlan (UK) strain). These were maintained on a 12/12-hour light/dark cycle (7 AM, lights on) and provided with ad libitum access to food and water. The day when vaginal plug was detected was referred to as E0. Embryos from both genders were analyzed at different developmental stages, as stated in the figures.

In ovo electroporation and tracing

The main purpose of our research is to describe comparable glutamatergic tangential migrations during chick telencephalic development. For this purpose, our electroporation method for labeling neural progenitors is likely the most powerful methodology. With this method, cells are enabled to migrate in vivo, inside the intact whole brain. It shows a direct relationship between the cells' origin in the VZ and their fate within the tissue days later. Our electroporation technique is able to show (1) whether the migration of telencephalic neurons actually happens, (2) the migratory route of these neurons, (3) the distribution of them after migration and (4) their identity. However, electroporation also shows some limitations. The number and location of progenitors transfected ranges from one animal to another in a way impossible to keep under control.

Electroporation of chick embryos was performed as previously described (Garcia-Moreno et al., 2014). For targeted electroporations of focal telencephalic areas the positive pole was placed next to the telencephalic areas to be electroporated, whereas the positive electrode was positioned at the opposite brain site.

Embryonic electroporation of reporter genes has been widely used to describe the lineage of neural stem cells. Here we exploit the piggybac (PB) transposase system (Ding et al., 2005) that delivers transgenes into the genome of the transfected cells. PB turns on the expression of the reporter gene permanently both in the electroporated stem cells and their whole progeny (Garcia-Moreno et al., 2014). In this work we electroporated chick embryos in ovo at the onset of telencephalic neurogenesis at E4, Hamburger and Hamilton (HH) stage 23-24 (Haumburger and Hamilton, 1951). We later investigated the labeled lineage at different time points, short-term tracing between E6-E8 (HH29-34) to observe the cells during the migratory process, and between E10-E14 (HH36-40) to describe the definitive long-term neuronal progeny at the end of telencephalic neurogenesis. The transposable system employed here offers two notable improvements for the study of cellular migration. First, as it labels stem progenitors permanently, these are always detectable. Therefore we can define the telencephalic identity of the specific ventricular sector where the electroporation was performed. And secondly, since the whole progeny inherits the genomic label from the progenitor, cell populations generated late after electroporation are also detectable, as opposed to traditional non-transposable electroporation. Moreover, this methodology enables native three-dimensional cellular trajectories in the whole intact live brain.

In other experiments, fluorescein dextran amine (3000MW) was injected within the embryonic neuroepithelium (E4). This way we obtained a more precise although not permanent labeling of progenitors (Garcia-Moreno et al., 2008; Metin et al., 2007).

In utero electroporation

Transfection by electroporation of embryonic neural progenitors was performed as described previously (Garcia-Moreno et al., 2010).

Plasmids

Most of the plasmid constructs employed in this study were employed in a previous study (Garcia-Moreno and Molnár, 2015), which describes these in detail. The concentration of different plasmids was kept constant among the different experiments in chick and mouse embryos. Labeling constructs (pPB-CAG-EYFP; pPB-CAG-MbEGFP) were transfected at a final concentration ranging between 200-500 ng/µl; the transposase enzyme expressing-construct (mPB) was consistently transfected at 300 ng/µl.

Tissue processing

Embryonic murine brains and chick embryonic brains up to E9 were fixed by immersion in 4% paraformaldehyde (PFA, diluted in phosphate buffered saline 0.1M– PBS, pH 7.3), whereas E10-E14 chick embryos were transcardially perfused with PBS followed by PFA. Brains were transferred to PBS 24h after fixation. All brains were sectioned in either the coronal or horizontal planes at 50-70 µm thickness in a vibrating microtome (Leica VT1000S).

Immunohistochemistry

Single and dual immunohistochemical reactions were performed as described previously (Garcia-Moreno et al., 2012) using the following primary antibodies: Rabbit antibody to EGFP (Molecular Probes, A11122, 1:1000), mouse antibody to EGFP (Abcam, ab1218, 1:1000), chick antibody to EGFP (Aves GFP 1020, 1:10000), rabbit anti calbindinD-28 (Swant, CB38, 1:10000), rabbit anti calretinin (Swant, 7697, 1:2000), rabbit antibody to Dbx1 (1:200; kind gift by Prof. Nakagawa, Univ. Minessota, US), rabbit antibody to PH3 (Chemicon, 06-570, 1:1,000), rabbit antibody to Tbr1 (Chemicon, AB9616, 1:1,000), rat antibody to Ctip2 (Abcam, ab18465, 1:500), rabbit antibody to dsRed2 (Takara, 632475, 1:1000), mouse antibody to Islet1 (DSHB, 39.4D5, 1:500) AND mouse antibody to Satb2 (Abcam, ab51502, 1:500). Dbx1 immunostaining required antigen retrieval with citrate acid.

For secondary antibodies (all 1:1000), we used Alexa 568 goat antibody to rabbit IgG (Molecular Probes, A11011), Alexa 647 goat antibody to rabbit IgG (Molecular Probes, A21245), Alexa 488 goat antibody to rabbit IgG (Molecular Probes, A11034), Alexa 488 goat antibody to mouse IgG (Molecular Probes, A11001), Alexa 568 antibody to mouse IgG (Molecular Probes, A11004), Alexa 568 goat antibody to rat IgG (Molecular Probes, A11077), and Alexa 488 goat antibody to chicken (Invitrogen, A11039,).

In situ hybridization

M13forward (5´GCCAGGGTTTTCCCAGTCAC3´) and M13reverse (5´GGAAACAGCTATGACCATG3´) primers were used to obtain the fragments from our cloned genes that were used to get riboprobes. Sense and antisense digoxygenin-11-UTP-labeled (Roche, Lewes, United Kingdom) riboprobes were synthesized according to the detailed procedures

previously described (Ferran et al., 2015). All the steps and procedures related with the extraction of brain samples, tissue processing and in situ hybridization in cryostat and floating sections were done as previously described (Ferran et al., 2015).

In silico analysis of potential regulatory elements

ATAC-seq data was downloaded from the ENCODE portal. Data for mouse forebrain at E11.5, E12.5 and E14.5 (ENCSR273UFV, ENCSR559FAJ and ENCSR810HQR, respectively; two replicates each) was used for the analysis. Raw ATAC-seq reads were mapped to the mouse genome (mm10 assembly) using bowtie2 (Langmead and Salzberg 2012) with the following options: --very-sensitive -X 2000 -I 0. Read pairs with a fragment length < 120bp were considered "nucleosome free" and subsequently used for peak calling. For this, MACS was used for low-thresholded peak calling using the following parameters: callpeak --nomodel --keep-dup 1 --llocal 10000 --extsize 74 --shift -37 -p 0.07 (middle track in UCSC browser screenshots). Next, the IDR framework (idr 0.1; https://github.com/nboley/idr) was used to determine high confidence peaks based on replicate information (bottom track; IDR). Density of ATAC-seq reads was displayed from bigwig files (top track).

To evaluate evolutionary conservation at a qualitative level, we used the PhyloP and conservation tracks from the UCSC browser. For the latter, we selected the following species: rat, rabbit, human, rhesus, cow, dog, elephant (Eutherian mammals); opossum, platypus (non-Eutherian mammals); chicken, zebra finch, painted turtle, xenopus tropicalis, coelacanth and zebrafish (non-mammalian verebrates). A putative enhancer (ATAC-seq IDR peaks in the three stages) was considered "mammalian-specific" if present in most Eutherian mammals (inferred to be present in the last common ancestor of Eutherians) but absent in all non-mammalian vertebrates.

Imaging and analysis

Images were captured using a Zeiss LSM 710 confocal microscope (Carl Zeiss Microimaging). Similar image parameters (laser power, gain, pinhole and wavelengths) were maintained for images from each brain and adjusted for new specimens. The employed fluorophores were DAPI, EGFP, Alexa 488, mCherry, Alexa 568 and Alexa 647. In selected cases, Z-stacks were taken individually for each channel and then collapsed to get maximum intensity projections. For panoramic views of big brain sections, tile-scan images were composed. Images were adjusted and analyzed using ImageJ (Image Analysis in Java, NIH) and Adobe Photoshop CS6 (Adobe Systems Inc.).

Images were taken from a minimum of 3 animals successfully electroporated in each pallial region (specific numbers for each experimental paradigms are included into the relevant figure legends). In addition to these numbers, dozens of other animals with broader electroporations were also analyzed and taken into consideration for the interpretations of results. This study is based on over 300 hundred chicken embryos electroporated and analyzed.

The lack of migratory cells arriving to DP from the different pallial sources did not need to be quantified, as there were no cells migrating to DP. Cells were counted on single confocal planes. Nuclear-tagged GFP made the counting more reliable. A minimum of three representative sections was taken into account to estimate the average number of cells per animal. Control and ect-rDBX1 animals were compared by t-Student test (significance $p<0.05$).

Abbreviations

The atlas “The chick brain in stereotaxic coordinates” by Puelles et al. (2007) primarily considers the Avian Brain Nomenclature Forum (Reiner et al., 2004), but it also applies a major developmental point of view over controversial regions. Having a developmental focus in our study, we have followed this atlas for our anatomical nomenclature in chick telencephalon.

Ac - anterior commissure

Ag - Amygdala

AuNP - Auditory nidopallium

BSS - Basal somatosensory nucleus of the nidopallium

cDVR - Caudal portion of the dorsal ventricular ridge

Cl - Claustrum

Cng - Cingulate cortex

cNP - Caudal region of the nidopallium

CP - Cortical plate

C-R - Cajal-Retzius cells

DP - Dorsal pallium

DVR - Dorsal ventricular ridge

GP - Globus pallidus

Hc - Hippocampus

HD -Densocellular hyperpallium

Hem -Cortical hem

Hyp - Hyperpallium

Ins - Insular cortex

IZ - Intermediate zone

L5 - Cortical layer 5

L6 - Cortical layer 6

lfb - Lateral forebrain bundle

LSt - Lateral striatum

LV - Lateral ventricle

MP - Medial pallium

MsP - Mesopallium

MSt - Medial striatum

MZ - Marginal zone

MtZ - Mantle zone

NP - Nidopallium

OB - Olfactory bulb

P - Pallium

PHA - Parahippocampal area

PO - Preoptic area

Pir - Piriform cortex

RMTW - Rostral medial telencephalic wall

Se - Septum

sNP - Superficial nidopallium

SP - Subpallium

St - Striatum

SVZ - Subventricular zone

TE - Thalamic eminence

VMS - Ventropallial migratory stream

VisCo - Visual nidopallial nucleus

vo - Ventral olfactory tract

VP - Ventral pallium

VZ - Ventricular zone

Supplementary Reference list

Ding, S., Wu, X., Li, G., Han, M., Zhuang, Y., Xu, T., 2005. Efficient Transposition of the piggyBac (PB) Transposon in Mammalian Cells and Mice. Cell 122, 473–483. doi:10.1016/j.cell.2005.07.013

Ferran, J.L., Ayad, A., Merchán, P., Morales-Delgado, N., Sánchez-Arrones, L., Alonso, A., SandovalSandoval, J.E., Bardet, S.M., Corral-San-Miguel, R., Sánchez-Guardado, L.Ó., Hidalgo-Sánchez, M., Martínez-de-la-Torre, M., Puelles, L., 2015. Exploring Brain Genoarchitecture by Single and Double Chromogenic In Situ Hybridization (ISH) and Immunohistochemistry (IHC) on Cryostat, Paraffin, or Floating Sections, in: Neuromethods, Neuromethods. Springer New York, New York, NY, pp. 83–107. doi:10.1007/978-1-4939-2303-8_5

Garcia-Moreno, F., López-Mascaraque, L., De Carlos, J.A., 2008. Early telencephalic migration topographically converging in the olfactory cortex. Cereb Cortex 18, 1239–1252. doi:10.1093/cercor/bhm154

Garcia-Moreno, F., Pedraza, M., Di Giovannantonio, L.G., Di Salvio, M., López-Mascaraque, L., Simeone, A., De Carlos, J.A., 2010. A neuronal migratory pathway crossing from diencephalon to telencephalon populates amygdala nuclei. Nat Neurosci 13, 680–689. doi:10.1038/nn.2556

Garcia-Moreno, F., Vasistha, N.A., Begbie, J., Molnár, Z., 2014. CLoNe is a new method to target single progenitors and study their progeny in mouse and chick. Development 141, 1589–1598. doi:10.1242/dev.105254

Hamburger, V., and Hamilton, H.L., 1951. A series of normal stages in the development of the chick embryo. J Morphol 88, 49–92.Metin et al., 2007

Puelles, L., Martinez de la Torre, M., Watson, C., Paxinos, G., Martinez, S., 2007. The Chick Brain in Stereotaxic Coordinates: An Atlas Featuring Neuromeric Subdivisions and Mammalian Homologies. Academic Press

Reiner, A., Perkel, D.J., Bruce, L.L., Butler, A.B., Csillag, A., Kuenzel, W., Medina, L., Paxinos, G., Shimizu, T., Striedter, G., Wild, M., Ball, G.F., Durand, S., Güntürkün, O., Lee, D.W., Mello, C.V., Powers, A., White, S.A., Hough, G., Kubikova, L., Smulders, T.V., Wada, K., Dugas-Ford, J., Husband, S., Yamamoto, K., Yu, J., Siang, C., Jarvis, E.D., Gütürkün, O., Avian Brain Nomenclature Forum, 2004. Revised nomenclature for avian telencephalon and some related brainstem nuclei. J Comp Neurol 473, 377–414. doi:10.1002/cne.20118

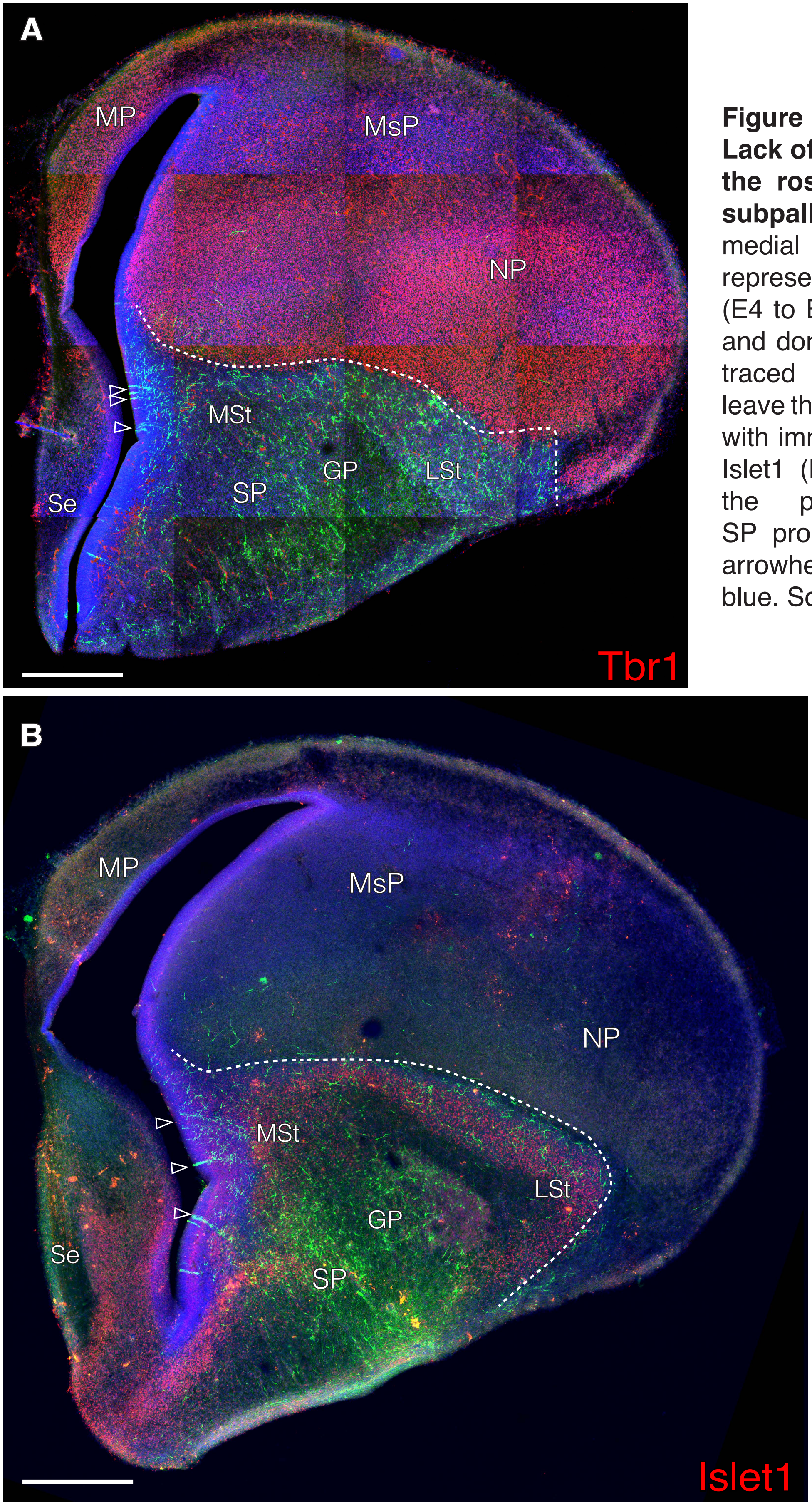

Figure S1. Related to Figure 2 – Lack of tangential migration from the rostral portion of the chick subpallium. Coronal sections, medial at the left. (A,B) Two representative images of long-term (E4 to E11) tracing from the rostral and dorsal subpallium. The lineage traced with GFP (green) did not leave the subpallial limits, as marked with immunostaining for Tbr1 (A) or Islet1 (B). Dashed line represents the pallial-subpallial boundary. SP progenitors are indicated with arrowheads. DAPI counterstain in blue. Scale bars represent 500 µm.

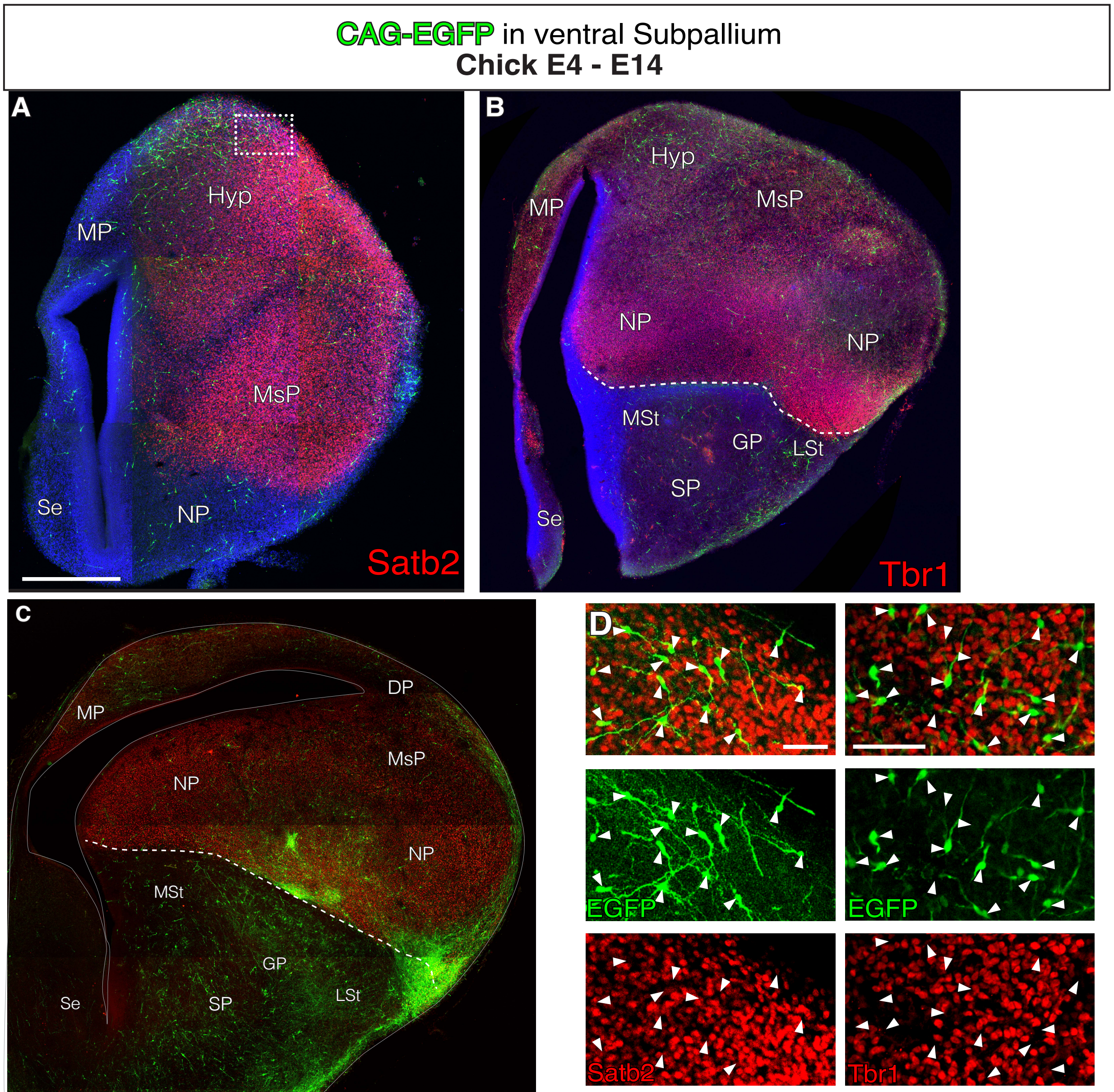

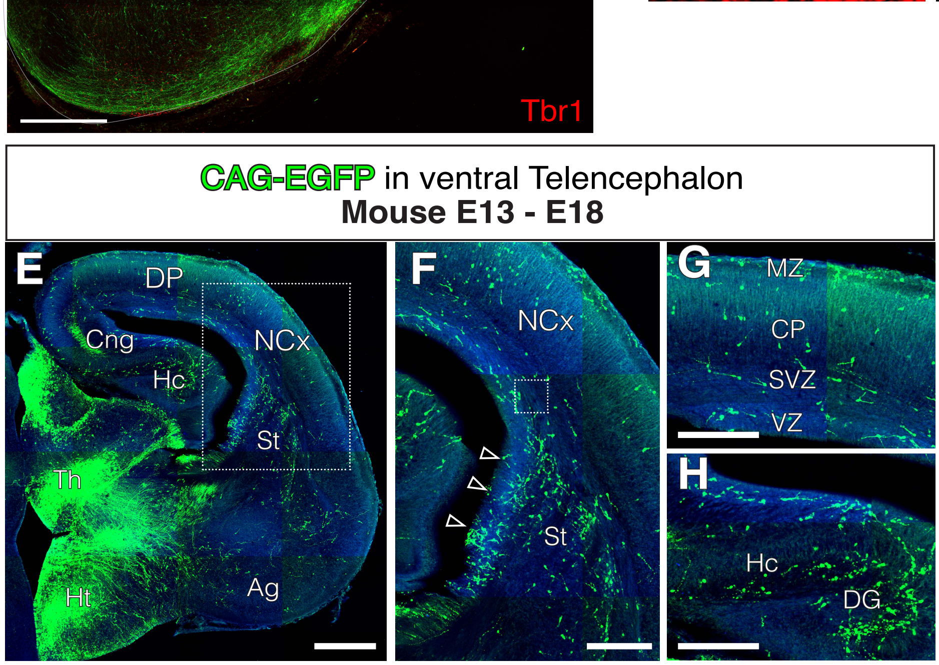

Figure S2. Related to Figure 2 – Tangential migration of interneurons from the cadual portion of the chick subpallium. Coronal sections, medial at the left. (A,B,C) Three representative images of long-term (E4 to E11) tracing from the caudal subpallium. The lineage traced with EGFP (green) migrated to and settled the whole pallium, as marked with immunostaining for Satb2 and Tbr1 (red). Dashed line represents the pallial-subpallial boundary. (D) High magnification showing EGFP derived neurons are neither Satb2 nor Tbr1 immunoreactive (arrowheads). (E-H) Electroporations on mice embryos at E13 on ventral telencephalic regions (including the ventral SP) labeled tangential migratory interneurons. (F) Power view of the rectangle in E. (G-H) Power views from the dorsal neocortex and hippocampus showing the horizontally-oriented tangential interneurons. DAPI counterstain in blue. Scale bars represent 500 µm in A-C,E; 250 µm in F-H; 50 µm in D.

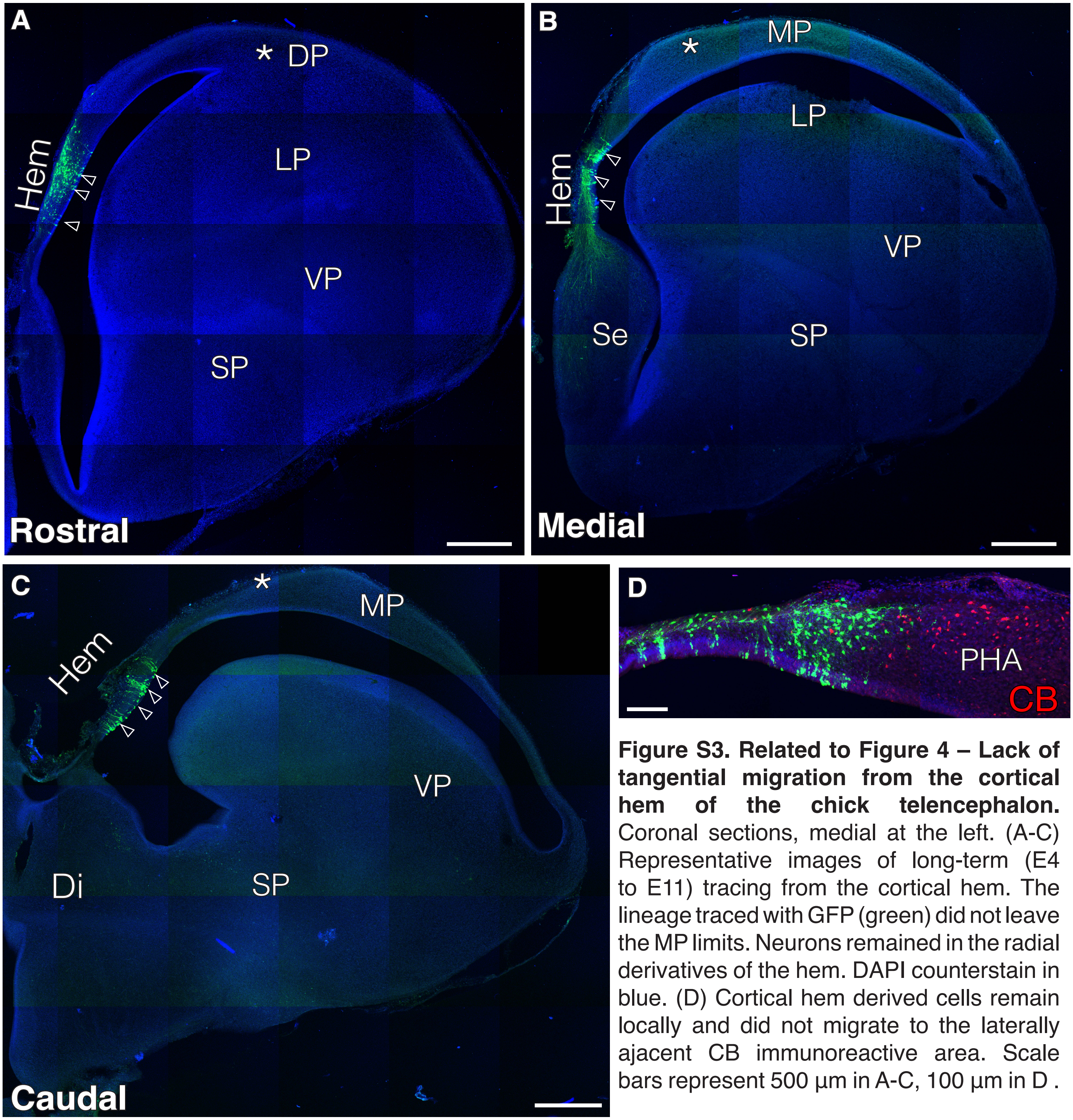

Figure S3. Related to Figure 4 – Lack of tangential migration from the cortical hem of the chick telencephalon. Coronal sections, medial at the left. (A-C) Representative images of long-term (E4 to E11) tracing from the cortical hem. The lineage traced with GFP (green) did not leave the MP limits. Neurons remained in the radial derivatives of the hem. DAPI counterstain in blue. (D) Cortical hem derived cells remain locally and did not migrate to the laterally ajacent CB immunoreactive area. Scale bars represent 500 µm in A-C, 100 µm in D .

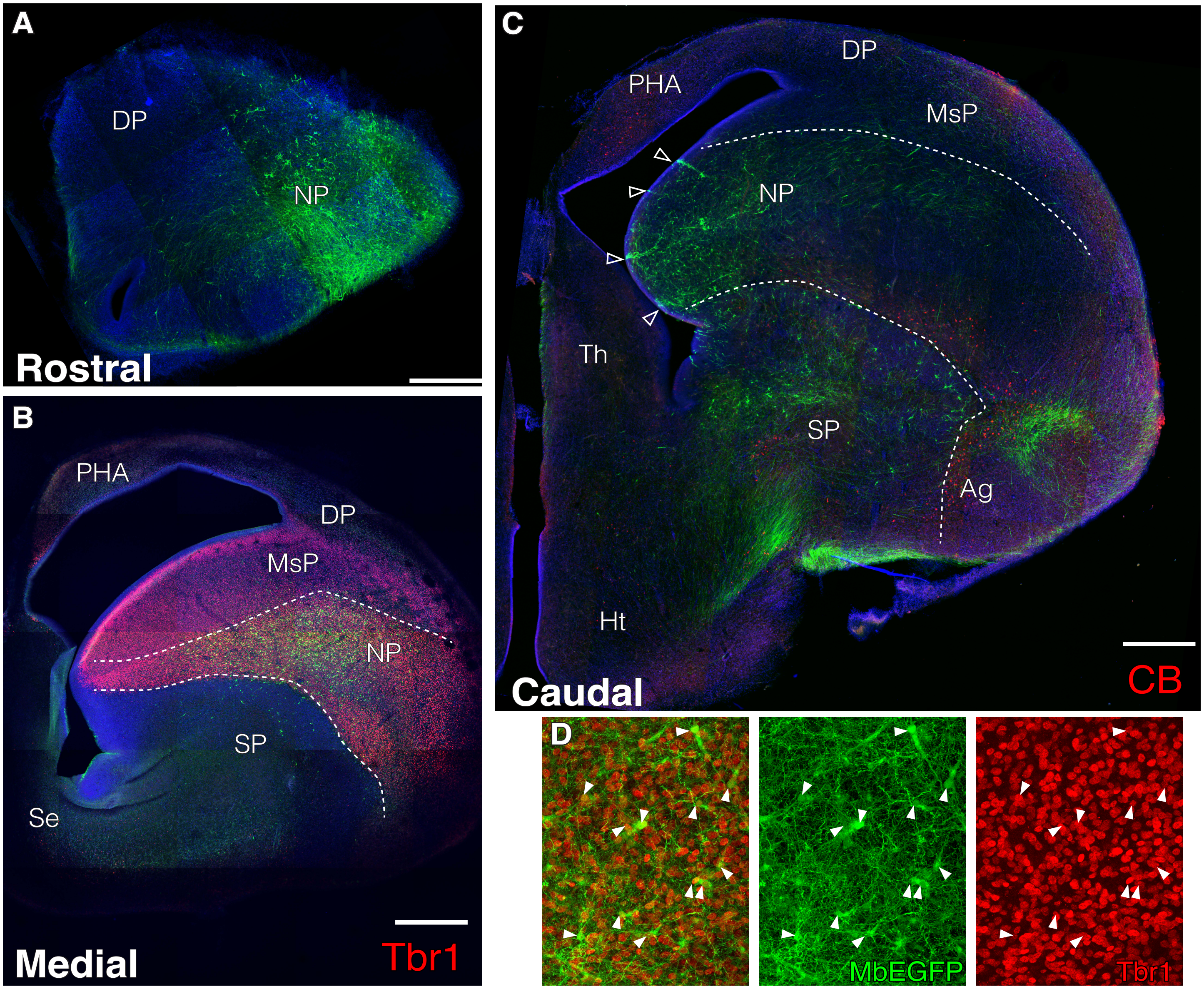

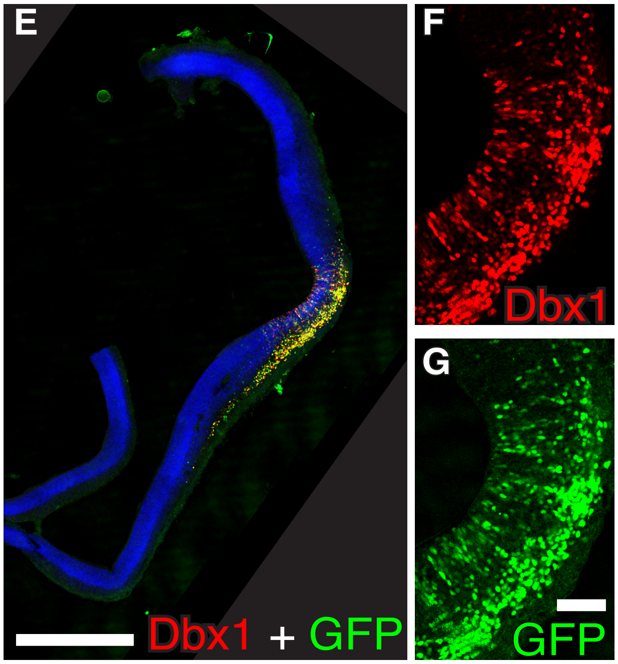

Figure S4. Related to Figures 5 and 6 – Radial migration from the chick ventral pallium and ectopic expression of rDbx1. Coronal sections, medial at the left. (A-C) Sections at three rostro-caudal levels representative of long-term (E4 to E11) tracing from the ventral pallium. The lineage traced with GFP (green) did not leave the nidopallial limits, as marked with immunostaining (red) for Tbr1 (B) or CB (C). Cells migrated from caudal portions of the brain (progenitors are indicated with arrowheads, C) to more superficial and rostral areas of the NP. Dashed line represents the ventral palliium boundaries. (D) Colocalization of membrane-tagged EGFP labeled cells with pallial marker Tbr1 (arrowheads) confirmed the VP-derived neurons are glutamtergic. (E-G) Ectopic expression of rDbx1 in E4 chick ventral pallium. Coronal sections at E6, medial at the left. Immunostaining for Dbx1 (red, F) and GFP (green, G) after electroporation of constructs for the expression of both proteins. DAPI counterstain in blue. Scale bars represent 500 µm in E, 100 µm in B,C. Scale bars represent 500 µm in A-C,E, 100 µm in F,G, 50 µm in D.

Figure S5. Related to Figure 7 – Regulatory landscape of Dbx1. Screenshot from the UCSC browser showing the potential regulatory landscape for the *Dbx1* locus in mouse (mm10). Tracks, from top to bottom: UCSC gene annotations, PhyloP score (placental mammal score; positive-blue indicates high conservation, whereas negative-red report accelerated sequence evolutionary rates), conservation (black/grey blocks show likely homologous regions), transposable element annotation by RepeatMasker, common SNPs, and, for each ATAC-seq sample (E11.5, E12.5 and E14.5), mapped read density, top 120,000 peaks genome-wide, high confidence IDR peaks. Red/blue boxes highlight regions with mammalian-specific/vertebrate-conserved peaks, which are zoomed out below. Orange box highlights a special case of an ATAC-seq peak overlapping largely a mammalian-specific sequence next to a non-coding region conserved across all studied vertebrates except frog.

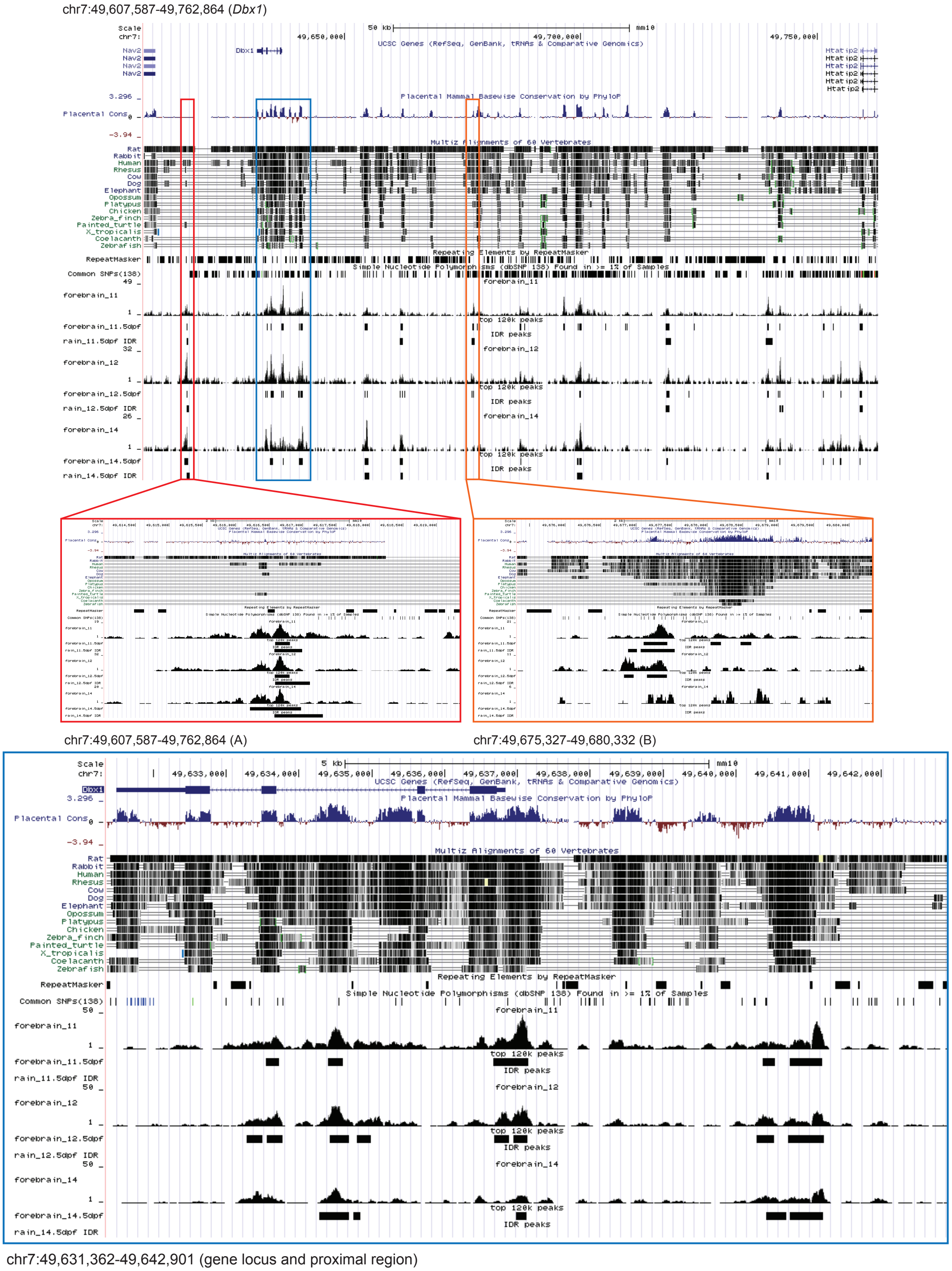

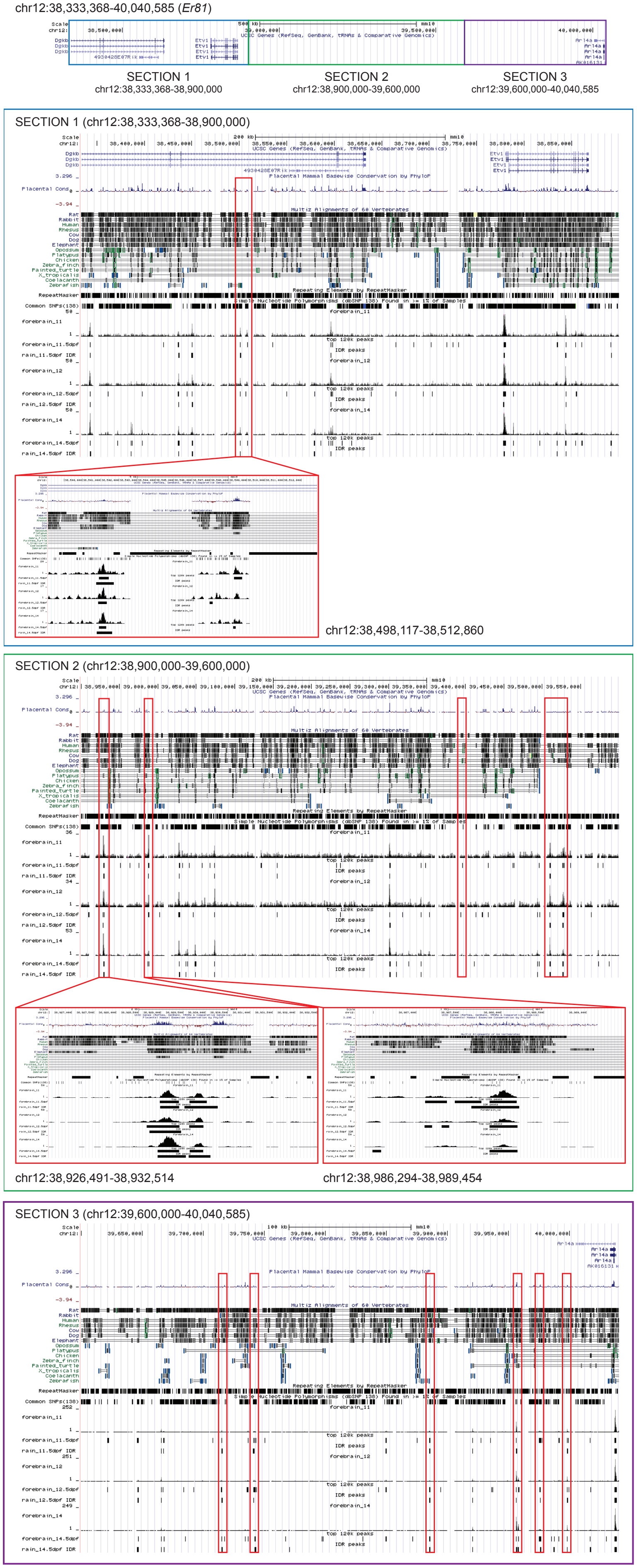

Figure S6. Related to Figure 7 – Regulatory landscape of Er81 (Etv1). The large (~1.7 Mbp) potential regulatory landscape of mouse *Etv1* was divided into three sections (A-C), for which screenshots from the UCSC browser (mm10) are shown below. Tracks, from top to bottom: UCSC gene annotations, PhyloP score (placental mammal score; positive-blue indicates high conservation, whereas negative-red report accelerated sequence evolutionary rates), conservation (black/grey blocks show likely homologous regions), transposable element annotation by RepeatMasker, common SNPs, and, for each ATAC-seq sample (E11.5, E12.5 and E14.5), mapped read density, top 120,000 peaks genome-wide, high confidence IDR peaks. Red boxes highlight regions with mammalian-specific peaks, some of which are enlarged below each section.

chr3:66,873,516-67,365,272 (*Shox2* - *Rsrc1*) chr2:31,617,538-31,670,120 (*Prdm12*)

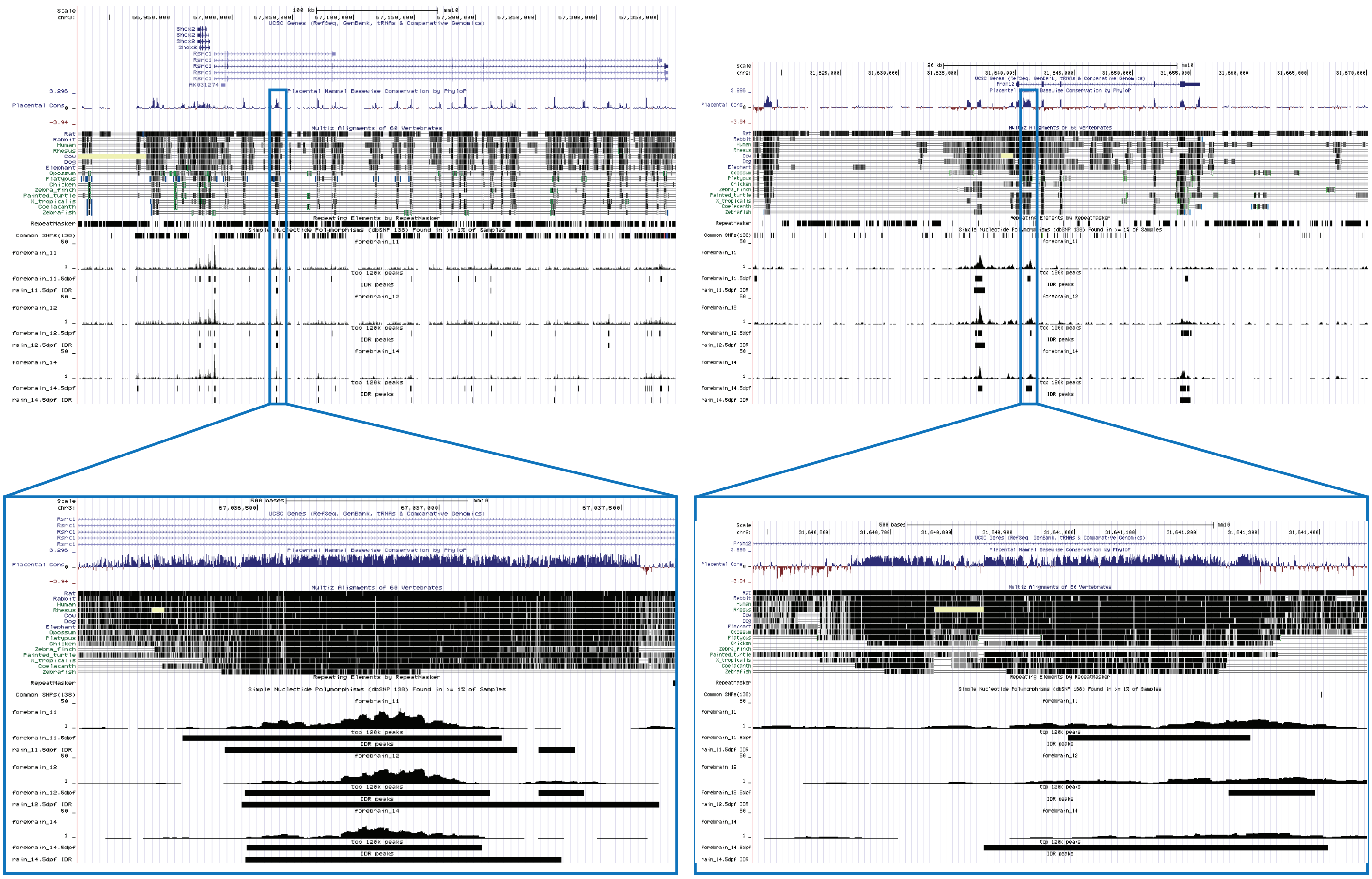

chr3:67,036,007-67,037,651 (hs636) chr2:31,640,477-31,641,478 (hs876)

Figure S7. Related to Figure 7 – Genomic features and conservation analysis of two VISTA enhancers (hs636 and hs876). Screenshot from the UCSC browser showing the genomic context (top) of two VISTA enhancers (hs636 and hs876; bottom). Tracks, from top to bottom: UCSC gene annotations, PhyloP score (placental mammal score; positive-blue indicates high conservation, whereas negative-red report accelerated sequence evolutionary rates), conservation (black/grey blocks show likely homologous regions), transposable element annotation by RepeatMasker, common SNPs, and, for each ATAC-seq sample (E11.5, E12.5 and E14.5), mapped read density, top 120,000 peaks genome-wide, high confidence IDR peaks.
