# Supplementary material for: Absence of Tangentially Migrating Glutamatergic Neurons in the Developing Avian Brain
Source: Cell Rep. 2018 Jan 2;22(1):96–109. doi: 10.1016/j.celrep.2017.12.032 (PMC5770341; doi:10.1016/j.celrep.2017.12.032)
Supplement: Document S2. Article plus Supplemental Information [file mmc2.pdf]

Cell Reports

Article

Absence of Tangentially Migrating Glutamatergic Neurons in the Developing Avian Brain

Graphical Abstract

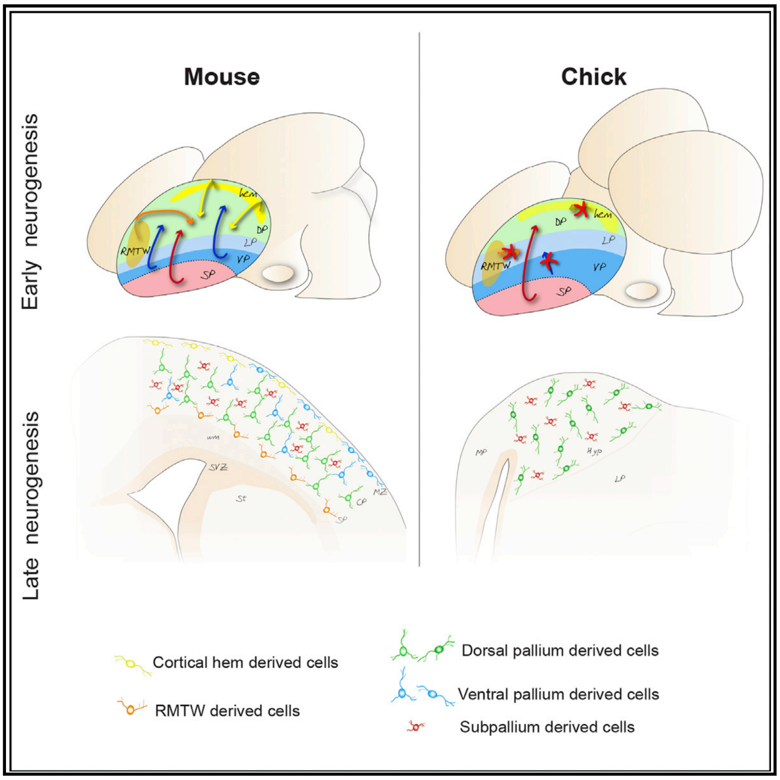

Authors

Fernando García-Moreno, Edward Anderton, Marta Jankowska, Jo Begbie, Juan Manuel Encinas, Manuel Irimia, Zoltán Molnár

Correspondence

fernando.garcia-moreno@achucarro.org (F.G.-M.), zoltan.molnar@dpag.ox.ac.uk (Z.M.)

In Brief

Neocortical formation crucially depends on the early tangential arrival of several transient glutamatergic neuronal populations. García-Moreno et al. find that these neuronal migrations are absent in the developing brain of chicks. The mammalian uniqueness of these developing migrations suggests a crucial role of these cells in the evolutionary origin of the neocortex.

Highlights

- Lack of tangential migration of Cajal-Retzius and subplate cells in chick
- No intra-pallial tangential migrations contribute to the dorsal pallium in chick
- Mammalian Dbx1 promotes differentiative divisions in chick ventral pallium
- Unique tangential migrations could have played a major role in cortical evolution

García-Moreno et al., 2018, Cell Reports *22*, 96–109
January 2, 2018 © 2017 The Authors.
https://doi.org/10.1016/j.celrep.2017.12.032

OPEN ACCESS
CellPress

Cell Reports
Article

Absence of Tangentially Migrating Glutamatergic Neurons in the Developing Avian Brain

Fernando García-Moreno,[1,2,3,8,*] Edward Anderton,[1] Marta Jankowska,[1,4,5] Jo Begbie,[1] Juan Manuel Encinas,[2,3] Manuel Irimia,[6,7] and Zoltán Molnár[1,*]
[1]Department of Physiology, Anatomy and Genetics, University of Oxford, Oxford OX1 3QX, UK
[2]Achucarro Basque Center for Neuroscience, Parque Científico UPV/EHU Edif. Sede, 48940 Leioa, Spain
[3]IKERBASQUE Foundation, María Díaz de Haro 3, 6th Floor, 48013 Bilbao, Spain
[4]College of Inter-Faculty Individual Studies in Mathematics and Natural Sciences, University of Warsaw, Banacha 2C, 02-097 Warsaw, Poland
[5]Faculty of Biology, University of Warsaw, Ilji Miecznikowa 1, 02-096 Warsaw, Poland
[6]EMBL/CRG Systems Biology Research Unit, Centre for Genomic Regulation (CRG), Barcelona Institute for Science and Technology, 08003 Barcelona, Spain
[7]Universitat Pompeu Fabra (UPF), 08003 Barcelona, Spain
[8]Lead Contact
*Correspondence: fernando.garcia-moreno@achucarro.org (F.G.-M.), zoltan.molnar@dpag.ox.ac.uk (Z.M.)
https://doi.org/10.1016/j.celrep.2017.12.032

SUMMARY

Several neuronal populations orchestrate neocortical development during mammalian embryogenesis. These include the glutamatergic subplate-, Cajal-Retzius-, and ventral pallium-derived populations, which coordinate cortical wiring, migration, and proliferation, respectively. These transient populations are primarily derived from other non-cortical pallial sources that migrate to the dorsal pallium. Are these migrations to the dorsal pallium conserved in amniotes or are they specific to mammals? Using *in ovo* electroporation, we traced the entire lineage of defined chick telencephalic progenitors. We found that several pallial sources that produce tangential migratory neurons in mammals only produced radially migrating neurons in the avian brain. Moreover, ectopic expression of VP-specific mammalian Dbx1 in avian brains altered neurogenesis but did not convert the migration into a mammal-like tangential movement. Together, these data indicate that tangential cellular contributions of glutamatergic neurons originate from outside the dorsal pallium and that pallial Dbx1 expression may underlie the generation of the mammalian neocortex during evolution.

INTRODUCTION

Understanding the functional and structural complexity of the neocortex requires knowledge of its evolutionary origin and embryonic development (Geschwind and Rakic, 2013). Because morphological evolution is underlain by changes in embryo development (Gould, 1977), modifications in the neurogenic programs of neocortical progenitors and alteration of the migratory patterns of derived neurons must have been key in shaping the mammalian brain. Comparative developmental analysis of homologous regions to the neocortex in non-mammalian brains provides insight into the origin of the cerebral cortex.

The mammalian neocortex is derived from the most dorsal region of the embryonic telencephalon, the dorsal pallium (DP) (Puelles, 2011). Most cortical excitatory glutamatergic neurons are born in the germinal zones of the DP and migrate radially toward the pial surface (Rakic, 1971). The vast majority of the inhibitory GABAergic interneurons are generated in the subpallium and migrate tangentially to the cortex (Anderson et al., 1997; de Carlos et al., 1996; Tremblay et al., 2016). In addition, several populations of glutamatergic neurons are generated outside of the neocortical neuroepithelium and travel to the DP through tangential migration (Barber and Pierani, 2016; Marín, 2013). These neurons are largely transient but play essential roles in orchestrating the telencephalic developmental program. Cajal-Retzius (C-R) cells settle early in the most superficial of the cortical layers, the marginal zone (MZ), from where they exert important roles in cortical formation, such as leading the inside-out neurogenic pattern (Beffert et al., 2004; Ogawa et al., 1995) and maintaining radial glial cell function (Supèr et al., 2000). Cajal-Retzius cells reach the neocortex by tangential migration from a variety of telencephalic origins, such as the cortical hem (García-Moreno et al., 2007), the rostral septum, and the ventral pallium (VP) (Bielle et al., 2005). Subplate neurons populate the deepest region of the cortical plate, from where they control the entry of early thalamic fibers into the cortex (Hoerder-Suabedissen and Molnár, 2015). Although their precise origin remains unclear, a subpopulation of subplate neurons characterized by the expression of the *Lpar1* gene is generated outside of the cortical boundaries (García-Moreno et al., 2008; Pedraza et al., 2014), in the rostral and medial region of the telencephalic wall. A population of transient glutamatergic pyramidal neurons also reaches the neocortex by tangential migration derived from the *Dbx1*-expressing region of the VP (Teissier et al., 2010). Ablation of Dbx1-expressing populations reduces cortical neuronal numbers by 20%, and, thus, it is believed that Dbx1-derived neurons with VP origin promote local cortical neurogenesis as they migrate to the cortex (Barber and Pierani, 2016).

 © 2017 The Authors.
This is an open access article under the CC BY license (http://creativecommons.org/licenses/by/4.0/).

Lineage tracing techniques have shown direct evidence for the extra-neocortical origins of the glutamatergic tangential migrations for both Cajal-Retzius cells (García-Moreno et al., 2007; Imayoshi et al., 2008) and subplate neurons (García-Moreno et al., 2008; Pedraza et al., 2014) in the mouse cortex. However, these techniques have not been directly applied to Dbx1-derived transient pyramidal neurons *in vivo*. In addition, despite their importance in mammalian cortical development, very little is known about these transient populations in non-mammalian species (Nomura et al., 2008; Puelles, 2011). Are such tangential contributions to the DP conserved among amniote brains or are they a mammalian novelty? Did their appearance coincide with or lead to the origin of the current six-layered neocortex? What developmentally divergent mechanisms regulate neurogenesis in the DP and VP in amniote brains?

Here we compared the generation and migration of various neuronal populations in mouse and chick. We performed *in ovo* electroporation to trace the complete lineages of chick neural progenitors from defined sectors of the telencephalon, aided by transposase-mediated permanent labeling of the progenitors. We describe that none of these tangentially migrating transient neuronal populations were present during avian dorsal pallial development. In addition, ectopic expression of the mammalian homeobox gene *Dbx1*, a transcription factor expressed in the mammalian but not avian VP, did not promote tangential migration in the chick brain, although it triggered more differentiative and less self-renewing neurogenesis. We propose that these two novelties in mammals, tangential arrival of the transient glutamatergic populations and the decrease of VP neurogenesis caused by *de novo* Dbx1 expression, could have been a major developmental divergence to drive mammalian cortical evolution.

RESULTS

Identification of Internal Boundaries of the Developing Chick Telencephalon

The avian telencephalon is comprised of a series of nuclear regions that are strikingly different from the predominantly laminated mammalian telencephalon (Jarvis et al., 2013; Reiner et al., 2005). To compare the different origins and fates of neuronal populations in the mature brain, we first established the positions and boundaries of the different pallial sectors in the chick brain after the completion of neurogenesis and most neuronal migrations. We used well-known markers of these sectors to reveal their boundaries, focusing on markers whose expression is constant during the neurogenic period. Tbr1 is a commonly used marker of both mouse and chick pallium (Puelles et al., 2000). In both early (embryonic day [E]6/E7) and late (E11) stages of chick neurogenesis, Tbr1 expression was detected in all pallial areas, defining a clear pallial-subpallial boundary (PSB; dashed lines in Figures 1A and 1D–1J). The brightest Tbr1 expression was found in the deep mesopallium (MsP) and in the nidopallium (NP), which derive from the lateral pallium (LP) and VP, respectively. We used Islet1 (Abellán and Medina, 2009) and Ctip2 (Suzuki et al., 2012) as markers to define the PSB because they are specifically expressed in the subpallial mantle zone (Figures 1B and 1E–1J). In addition, Ctip2 exhibited low expression in other telencephalic areas because it was detected in the parahippocampal area derived from the medial pallium (MP) (Figures 1E–1H). Satb2 (Suzuki and Hirata, 2014; Suzuki et al., 2012) delineated the boundary between the LP and VP (Figure 1C and 1E-J). Satb2 was highly expressed in the LP derivatives and showed nearly no expression in the NP, apart from some faintly bright cells in the visual NP region.

We identified the location of the DP by differential expression of selected markers. Ctip2 was expressed more in the MP than in the DP; Satb2 defined the MP-to-DP limit because it is nearly absent in the rostral MP (Figures 1C and 1E–1J). The ventral border of the DP was identified using Satb2 and Tbr1; the former is expressed in the hyperpallial columns to a variable degree, whereas DP has a low level of Tbr1 expression.

The establishment of these pallial areas was used to define the origin and cellular destination of the different telencephalic populations studied.

Pallial GABAergic Interneurons Originate in a Restricted and Conserved Subpallial Region

The first populations we investigated were GABAergic interneurons. In mammals, interneurons have subpallial (SP) origins and migrate to the neocortex tangentially (Anderson et al., 1997; de Carlos et al., 1996). Although homologous tangential migration has been described in several vertebrate species (Carrera et al., 2008; Cobos et al., 2001a; Métin et al., 2007; Moreno et al., 2008; Tuorto et al., 2003), the specific telencephalic sector that gives rise to the avian pallial interneuronal population remains undefined (Cobos et al., 2001a; Tuorto et al., 2003).

We transfected chick telencephalic progenitors on E4, the earliest telencephalic neurogenesis in chick, by permanent *in vivo* lineage tracing (Supplemental Experimental Procedures), which enables the identification of the electroporated progenitor region. When we labeled chick subpallial progenitors, we found two different cell lineages (Figures 2 and S1). First, the rostral and dorsal regions of the SP (striatal SP), adjacent to the ventral border of the VP, produced an exclusively radially migrating progeny (Figure 2A) because short-term lineage tracing showed that subpallial cells did not cross the PSB 2 or 3 days after electroporation (Figures 2C and 2D). Later, cells that originated at the SP territory settled radially, in the striatal Islet1+/Tbr1− areas (E11; Figures 2E–2G). Derived cells settled from the lateral striatum, near the pial surface, to the deepest nuclear regions of the medial striatum. Strikingly, cells remained in the SP and did not cross the PSB.

Electroporations at more posterior and ventral subpallial sectors (pallidal SP) showed the expected tangential waves of interneuron migration (Figures 2B and 2H–2T). On E7, 3 days after electroporation, large cohorts of neurons migrated tangentially, crossed the PSB, and invaded both the adjacent VP and LP, reaching the distant DP in some cases (Figures 2H–2K). Neurons did not restrict their movement to a particular stratum of the pallial mantle zone because they migrated through and settled along the entire depth of the pallium (Figure 2L). This migration followed a rostro-caudal pattern; cells traveled dorsally from the caudal regions of the SP to more anterior and posterior regions of the pallium (Figures 2H and 2K). Long-term tracing experiments, aided by transposase-mediated permanent labeling, showed a large number of neurons occupying the entire pallial extension, spanning from the NP to the parahippocampal area

OPEN ACCESS
CellPress

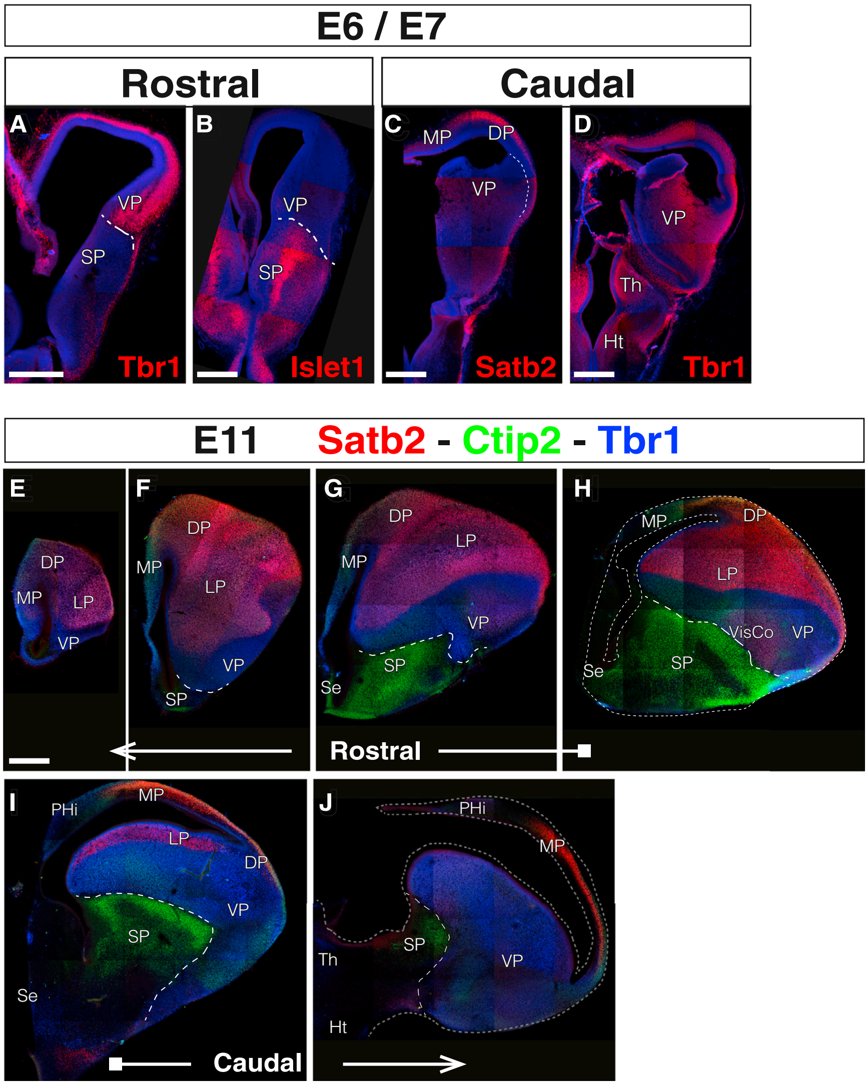

Figure 1. Pallial Subdivisions in the Chick Telencephalon at Early and Late Neurogenesis

Shown are coronal sections, medial at the left.

(A–D) Immunostainings for pallial (Tbr1, A and D; Satb2, C) and subpallial (Islet1, B) markers on early embryos (E6–E7). DAPI counterstain is shown in in blue.

(E–J) Immunostainings of pallial (Tbr1, blue; Satb2, red) and subpallial (Ctip2, green) markers at the end of neurogenesis (E11). The images show different anterior (E and F), middle (G and H), and posterior (I and J) levels of the telencephalon.

Dashed lines mark the PSB in (A), (B), and (E)–(J) and the boundary between the LP and VP in (C).

Scale bars represent 500 μm; the scale in E applies to (E)–(J).

(MP-derived). These neurons were found to be negative for glutamatergic markers Satb2 and Tbr1, which confirmed their GABAergic nature (Figure S2). Many cells were also found to remain in the SP (mainly the pallidal Islet1– region; Figures 2B, 2M–2T and S2).

Taken together, these experiments revealed that tangentially migrating interneurons originate from a specific limited region of the chick SP. In addition, these experiments confirmed that the permanent staining by PB transposition (García-Moreno et al., 2014) is an efficient and sensitive method for labeling tangential migrations and identifying singular progenitor niches.

Lack of Dorsal Tangential Migration from the Rostral Medial Telencephalic Wall in the Chick Brain

Subplate neurons are a heterogeneous cell population and are generated in diverse neurogenic niches (Hoerder-Suabedissen and Molnár, 2015). One of these niches is the murine rostral medial telencephalic wall (RMTW). This sector gives rise to a population of tangentially migrating Lpar1-expressing glutamatergic neurons that populate the subplate layer (Pedraza et al., 2014; Figure 3A). We investigated the presence of this particular subset of DP-colonizing migratory cells in the chick brain. Our electroporations covered the entire RMTW, which comprises germinative zones in the pallium (MP) and SP (rostral septum) (Figure 3).

Short-term lineage tracing (E4 to E7) from the RMTW revealed two different migratory behaviors of the derived cells. The majority of newly generated neurons migrated radially to the surface of the pallial and subpallial regions of the RMTW (Figures 3C–3F). Additionally, a stream of cells migrated ventrally toward the septal surface (Figure 3F), similarly to migrations described in the mouse (García-Moreno et al., 2008). However, no dorsal trajectory was found in the chick. Similarly divergent migrations were observed in long-term experiments, in which the transposase guarantees the labeling of the entire progeny from E4 to E11 (Figures 3G–3M). Germinative zones of the RMTW contributed only to the overlying mantle zone of the rostral MP and septal area. More rostrally, the RMTW cells settled to the anterior olfactory region above the olfactory bulb in the VP, alongside the olfactory bulb itself (Figures 3G and 3M). However, RMTW-derived cells in the chick never invaded the distant DP (asterisks in Figure 3). The stream of cells migrating ventrally came to populate the surface of the septum, diagonal band, lateral striatum, and superficial olfactory areas next to the ventral olfactory tract (Figures 3I and 3J). These experiments suggest that subplate-like cells derived from the RMTW do not contribute to the avian DP (Figure 3B).

Absence of Tangential Migration of Cajal-Retzius-like Neurons in the Chick Telencephalon

In the mammalian brain, several pallial germinative zones generate Cajal-Retzius cells (Barber and Pierani, 2016). We

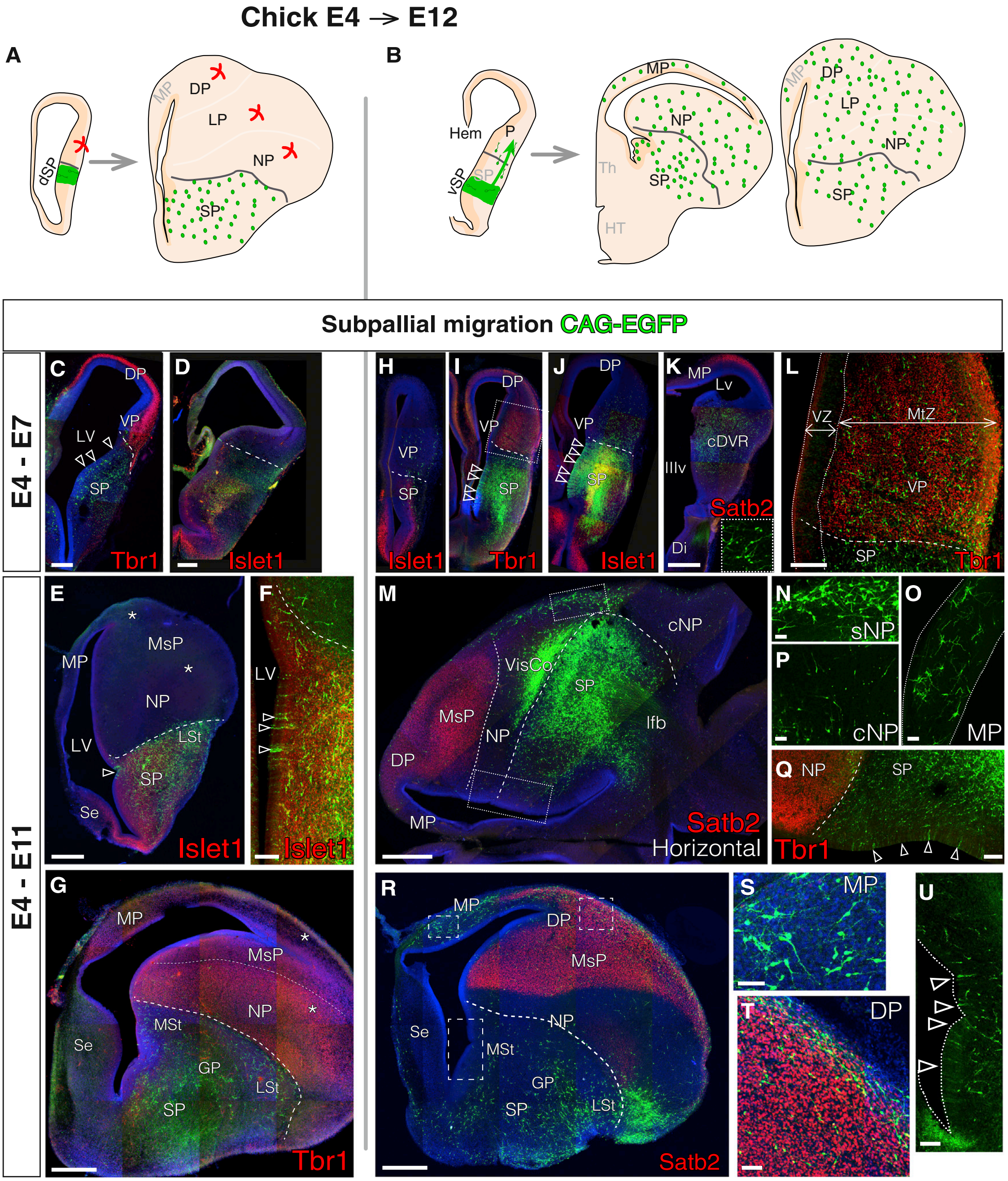

Figure 2. Restricted Subpallial Origin of Chick Pallial Interneurons

Shown are coronal sections, medial at the left.

(A and B) Schematic diagrams depicting the lineage of dorsal (dSP, A) and ventral (vSP, B) SP progenitors.

(C and D) The short-term lineage of dSPs (green) does not cross the PSB at E7, delineated by Tbr1 (C) and Islet1 (D) immunostaining (red, n = 3).

(E–G) dSP progeny remains within SP boundaries at the end of neurogenesis (E11, n = 4). Neurons do not enter the pallium (asterisks). Section in (E) is more anterior to (G). The electroporated SP area lies just ventral to the VP, as shown in (F).

(H–K) Rostral-to-caudal examples of the short-term tracing from vSPs (rostral, H; middle, I and J; caudal, K). GFP cells migrated tangentially in the rostro-caudal and ventro-dorsal axes, crossing the PSB (n = 4).

(L) Power view of the rectangle depicted in (I), showing that vSP cells migrate through the entire pallial mantle zone (MtZ) depth marked with Tbr1.

(M–U) Long-term tracing colonized the whole pallium at E11 (n = 6). Horizontal (M–Q, medial at the bottom, rostral at the left) or coronal (R–U) sections show that GFP cells entered the pallium, including the DP, superficial NP (N), medial pallium (O), and caudal NP (P). (N)–(Q) show power views of the rectangles

(legend continued on next page)

searched for these migratory cells in the avian DP, focusing first on the medial source of Cajal-Retzius cells, the cortical hem (Figure 4). This structure appears from medial to caudal levels of the MP, at its most distant and ventral region (Grove et al., 1998; Abellán et al., 2014), and, in the mouse, it produces migratory cells that settle early in the DP (Figure 4A). Short-term electroporations of the presumptive chick cortical hem on E4 revealed the absence of a tangentially migrating neuronal lineage during early telencephalic neurogenesis (Figures 4C–4G). Because of the reduced size of the cortical hem, electroporations occasionally extended to other, unintended MP progenitors. However, neither cortical hem nor MP progenitors produced tangentially migrating cells. The lineage of these progenitors only occupied MP areas and never reached the distant DP regions (Figures 4C–4G). Furthermore, when the long-term lineage was analyzed at the end of neurogenesis (E11), no MP-derived neurons were found in the DP (Figures 4H–4J and S3). The transposase-mediated labeling confirmed that cortical hem neurons generated from E4 to E11 did not migrate to the DP. Therefore, these progeny-tracing experiments suggest that the avian cortical hem does not produce tangentially migrating Cajal-Retzius-like cells (Figure 4B).

Early murine Dbx1+VP progenitors give rise to tangentially migrating Cajal-Retzius cells that populate the neocortex (Figure 5A). Electroporations of the early chick VP labeled a very large radially migrating progeny toward the dorsal ventricular ridge (DVR) mantle zone (Figure 5B). In short-term tracing experiments, no tangentially migrating cells were found to be directed toward more dorsal regions of the pallium (Figures 5C and 5D). This lack of VP-derived tangential migration was consistent from anterior to posterior regions of the telencephalon. The focus of our electroporation ranged from very extensive, covering the entire ventral and lateral pallial or subpallial ventricular regions, to a much smaller and precise sector of VP progenitors. We also performed experiments targeting a smaller but more precise labeling of progenitors using injections of the cell tracker (García-Moreno et al., 2008) fluorescein dextran amine (FDA) in E4 embryos. Cells generated in the small and circumscribed injection site in the VP migrated only radially, and at E9, they occupied the whole depth of the VP (Figure 5E).

Together with the absence of cortical hem-derived tangential migration (Figure 4), this set of experiments in the VP indicates that the avian DP does not receive a population homologous to the tangentially migrating Cajal-Retzius cells in mammals.

The Avian VP Does Not Provide Neurons for the DP

Observations in the Dbx1-LacZ mouse model suggest that, at mid-stages of neurogenesis, Dbx1+VP progeny migrate tangentially to the cortical plate, where they settle to differentiate into a transient glutamatergic pyramidal neuron population (Teissier et al., 2010). We investigated the presence of such tangential migration in the avian brain using long-term permanent tracing. If avian VP progenitors generate a tangentially migrating neuronal population, then our permanent transposase-driven labeling should identify such neurons derived from this germinative region in the chick brain. However, our long-term experiments, in which the transposase guarantees the labeling of the entire progeny from E4 to E11, revealed only radial and not tangential migrations from the VP during mid- and late-neurogenesis in the chick (Figures 5F–5L and S4). We tested this across different rostro-caudal levels of the chick VP, and the lineages detected were invariably radial, extending from caudal-deep regions to rostral-superficial regions of the NP. VP-derived neurons settled at rostral levels in the olfactory, visual, and somatosensory NP areas (Figures 5F and 5G), in the auditory NP, and amygdalar nuclei at the caudal DVR (Figures 5H, 5I, and 5L). With the rare exception of a few neurons in the ventral mesopallium (Figure 5F), no cells were found in the hyperpallium or other DP-derived regions (asterisks in Figure 5).

Similar to RMTW-derived subplate cells (Figure 3) and Cajal-Retzius cells (Figures 4 and 5), no glutamatergic VP-derived neurons were found in the DP of developing chick brains.

Ectopic Expression of Mammalian *Dbx1* in the Chick VP Modifies Neurogenesis

A crucial difference between the VP germinative zones (GZs) in mammals and avian brains is the expression of DBX1. Unlike in mammals, avian VP progenitors do not express DBX1 (Bielle et al., 2005). We hypothesized that recruitment of DBX1 expression in the ancestral mammalian VP progenitors may have been partly responsible for the divergence of developmental programs during evolution, leading to reduced neurogenesis in VP and tangential migration of VP-derived neurons in mammals.

To test this hypothesis, we performed ectopic expression of DBX1 (ect-rDBX1) in E4 chick VP by electroporating a nuclearly tagged EGFP plasmid together with the pCAX-rDbx1 construct or without it (a plasmid that expresses the rat *Dbx1* gene; ectopic or control experiment, respectively). Embryos were harvested on E6. The results revealed that ect-rDBX1 did not modify the migratory behavior of VP-derived cells (Figures 6 and S4). The short-term lineage experiment showed migrations in early embryos to be exclusively radial (Figures 6A–6G; as described in Figures 5C–5E). No changes in migration or neuronal morphology were detected after ect-rDBX1.

We also analyzed the neurogenic properties of ect-rDBX1 progenitors by comparing the proportion of neurons derived from each VP progenitor. Given that the boundary between the VP and LP at these early stages is not easily distinguishable (Figure 1), we defined VP as the region 300 μm dorsal to the PSB. Because a variable number of SP progenitors can also be labeled in most experiments because of the very small territory of the VP at E4, all EGFP+/Tbr1− cells in the mantle zone (MtZ) were subtracted to account for the migration of SP-derived interneurons. In ect-rDBX1 chicks, the number of neurons derived per VP progenitor doubled, defined as the ratio of EGFP+ cells

shown in (M). (T) and (U) show power views of the rectangles shown in (R). Dashed lines mark the PSB. The dotted lines in (M) define the boundary between the LP and VP.

Empty arrowheads point to transfected SP progenitors. DAPI counterstain is shown in blue. Scale bars represent 500 μm in (E), (G), (M), (V), and (K) for (H)–(K); 250 μm in (D); 100 μm in (F), (L), (Q), and (U); and 50 μm in (N), (O), (S), and (T).

See also Figures S1 and S2.

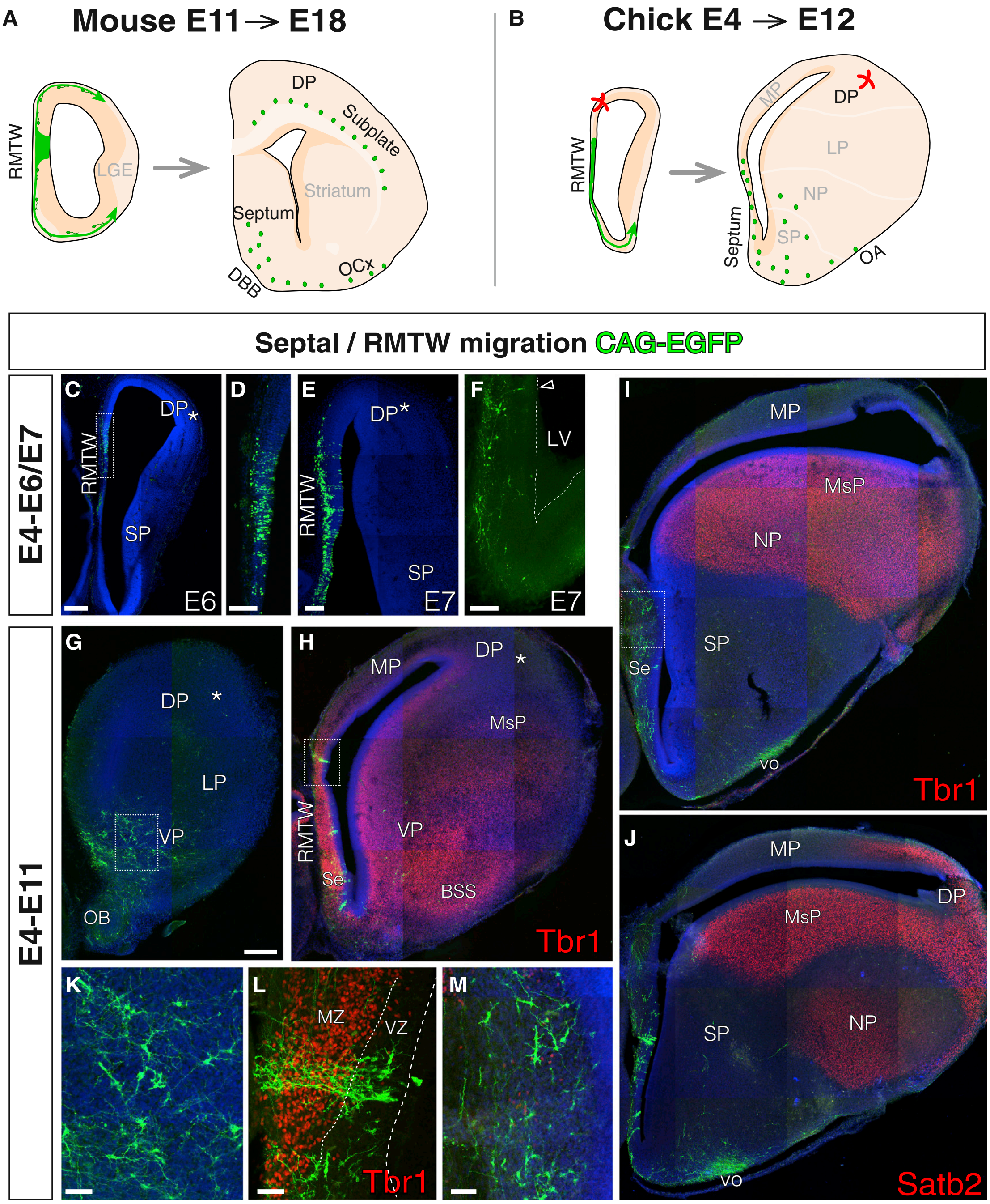

Figure 3. Only Ventral but No Dorsal Migration from the Avian Rostral Medial Telencephalic Wall

Shown are coronal sections, medial at the left.

(A and B) Schematic diagrams summarizing the lineage of RMTW progenitors in mouse (A) and chick (B).

(C–F) Rostral sections from two different animals (C and E) and power magnifications (D) of the telencephalon, showing that GFP-labeled cells remain locally after short-term tracing (n = 3) and do not migrate dorsally toward the DP region (marked with an asterisk) but ventrally toward septal areas (F).

(G–J) Rostral-to-caudal examples of long-term tracing from the RMTW supported by immunostaining for the pallial markers Tbr1 and Satb2 (n = 3). Labeled progenitors are visible in the RMTW in (H); the progeny migrates ventrally, reaching rostral (G) and caudal regions (I and J) of the septal and olfactory systems. No cells were found to migrate dorsally toward either the lateral regions of MP or DP (asterisks). Axons from the olfactory bulb cells were visible, traveling caudally by the ventral olfactory tract (vo).

(K–M) Power magnifications of the rectangles (K, L, and M for rectangles in G, H, and I, respectively), showing the morphology of labeled neurons. The progenitors in (M) are pallial, as they lie down the Tbr1+ postmitotic region of the RMTW.

DAPI counterstain is shown in blue. Scale bars represent 250 μm in (C) and (G) for (G)–(I); 100 μm in (D) and (E); and 50 μm in (K)–(M).

OPEN
ACCESS
CellPress

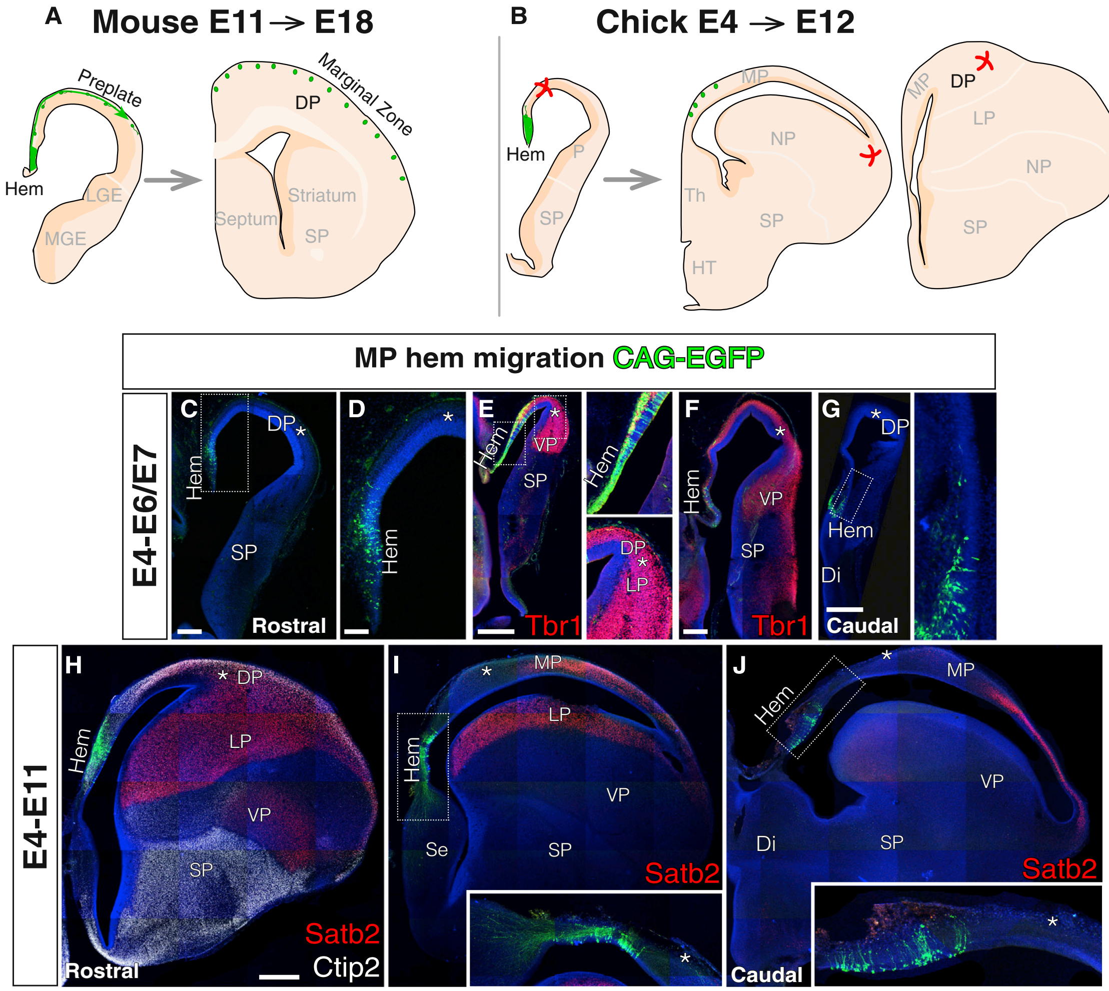

Figure 4. Lack of Tangential Migration from the Avian Cortical Hem

Coronal sections are shown, medial at the left.

(A and B) Schematic diagrams depicting the lineage of cortical hem progenitors in mouse (A) and chick (B).

(C–G) Rostral-to-caudal series of sections (rostral, C; middle, E; caudal, F and G) of early embryos electroporated at the cortical hem (n = 3) and its power magnification (D for C). GFP cells did not migrate dorsally toward the DP (asterisk) at E6/E7. Even when other MP progenitors next to the cortical hem were transfected (E and F), the lineage remained locally. Tbr1 immunostaining supported regional identification.

(H–J) Examples of telencephalic sections after long-term tracing from the cortical hem (E11, n = 3), immunostained for Satb2 (red) and Ctip2 in (H) (white). At the end of neurogenesis, cortical hem progenitors were still visible and displayed a radial morphology (see insets in I and J). Cortical hem derivatives did not migrate through the MP toward the DP and settled locally in the most ventral part of the MP.

DAPI counterstain is shown in blue. Scale bars represent 500 μm in (E), (G), and (H) for (H)–(J); 250 μm in (C) and (F); and 100 μm in (D).

See also Figure S3.

in the MtZ versus the GZs (Figure 6H; control, 1.53 ± 0.18; ect-rDBX1, 3.07 ± 1.08; mean ± SD, $p = 0.029$). In contrast to control animals, in the majority of ect-rDBX1 electroporated animals, the VP progenitor pool was depleted 2 days post-electroporation (Figure 6G). Together, these data suggest that ect-rDBX1 induced terminal differentiation, leading to premature depletion of the VP progenitor pool after generating new neurons or transient SVZ progenitors.

In addition, the number of mitotic (PH3-immunoreactive) nuclei in the chick SVZ was four times higher after ect-rDBX1 expression compared with the control (Figures 6M and 6N; control, 13.53 ± 1.98; ect-rDBX1, 53.16 ± 28.72; $p = 0.030$), whereas no differences were observed in the VZ. Furthermore, these mitotic nuclei appeared to cluster and form groups of 30–80 cells, which were not seen in control chick brains (Figures 6I–6L). Almost no cells in these clusters were EGFP+ (Figure 6L). This may indicate that the mechanism promoting SVZ proliferation was non-cell-autonomous.

These experiments showed that, although it did not confer a tangential phenotype to the neuronal lineage, ectopic expression of rDBX1 in the chick VP altered neurogenesis in two ways. First, chick VP divisions were more differentiative and

OPEN
ACCESS
CellPress

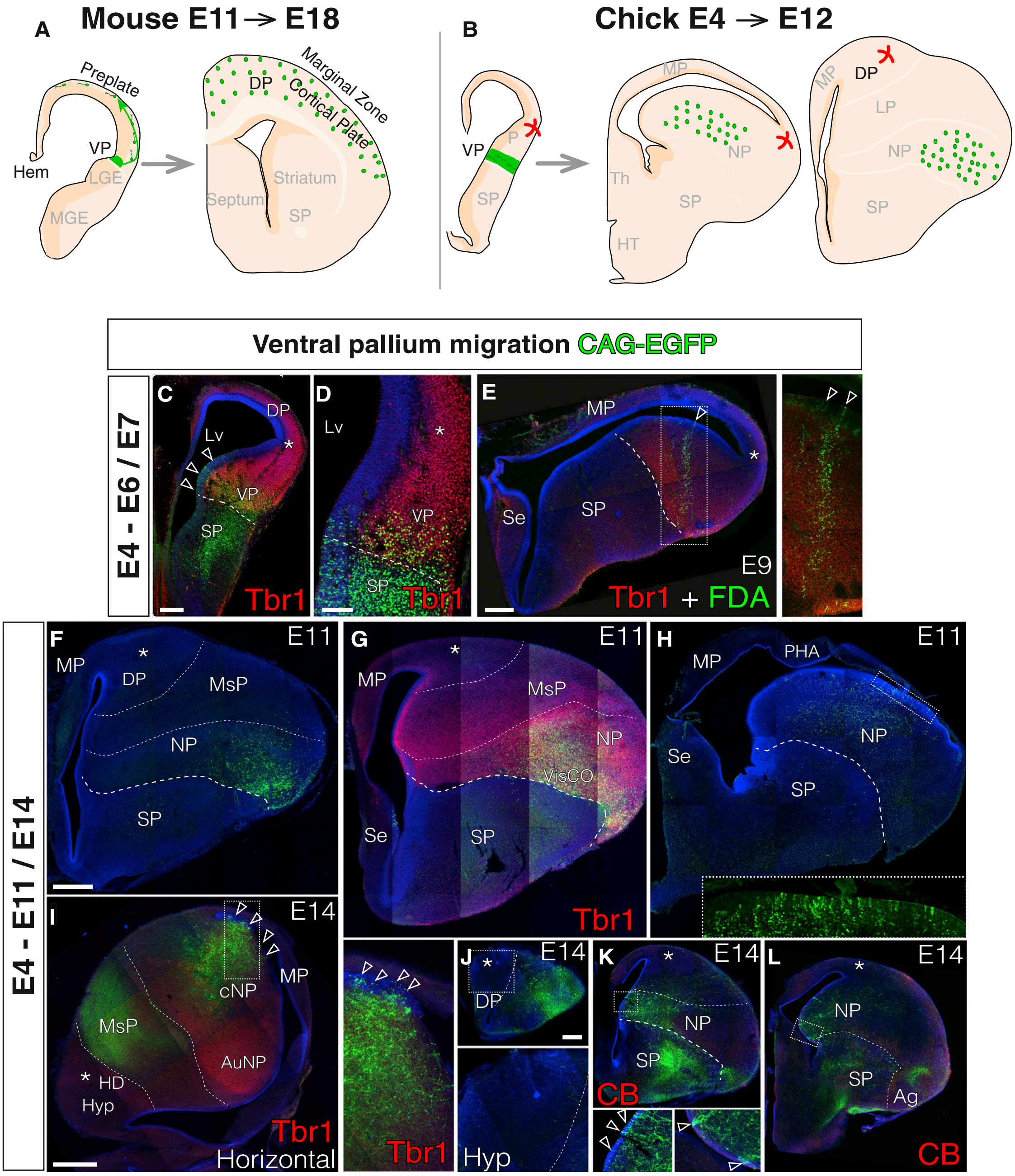

Figure 5. Exclusively Radial Derivatives from the Chick VP

Coronal sections are shown (medial at the left), except in (I), which is a horizontal section (medial at the bottom, rostral at the left).

(A and B) Schematic diagrams depicting the lineage of VP progenitors in mouse (A) and chick (B).

(C and D) Short-term electroporations at the VP produced a progeny that only settled radially within VP limits (E6/E7, n = 6). (D) Power magnification of another specimen (C). Pallial Tbr1 immunostaining confirmed the VP regional identity of the labeled progenitors. No tangential migration toward dorsal areas of the pallium (asterisk) was found.

(E) Columnar radial derivatives within the VP 5 days after FDA injection in the VP GZ (E9, n = 2). The rectangle is magnified in the right inset. Tbr1 immunostaining showed that the traced neuronal column belongs to the VP.

(F–L) Series of sections representative of three independent long-term lineage studies (F–H; I and J–L show calbindin and Tbr1 immunostaining; analysis based on n = 5). Derived cells at E11 were confined to the VP derivatives (NP). VP-labeled progenitors occupied caudal positions to the superficial lineage traced. The membrane-tagged EGFP reporter was used in (I)–(L), showing major VP projections to either the MsP (I) or through the SP toward the diencephalon (ventral

(legend continued on next page)

less self-renewing in ect-rDBX1animals. And second, proliferation in SVZ increased, and this may be due to an additional non-cell-autonomous effect.

Potential Regulatory Differences behind Distinct Behaviors in Avian and Mammalian VPs

In search of the mechanisms driving the divergent migration of VP neurons, we aimed to find transcription factor genes whose expression differs qualitatively in the developing VP of both species (Figure 7). Despite high a similarity in the expression patterns of transcription factors in early telencephalic progenitors, we could identify 4 genes that display divergent expression patterns: *Dbx1* and *Etv1* were found to be expressed in the murine VP but not in the chick VP (Figures 7A–7B'), whereas *RorA* and *Dach2* showed the opposite expression pattern in the two species (Figure 7C–7D'). We suggest that the mammalian novel expression of any of these genes, or a combination of them, could be responsible for the different migratory behavior of VP cells. To get further insights into the potential regulatory differences leading to these distinct behaviors, we next analyzed the regulatory landscapes of two genes that are expressed only in the mammalian VP to identify putative mammal-specific enhancers driving this expression. We used chromatin accessibility data (assay for transposase-accessible chromatin using sequencing [ATAC-seq]) generated by the Encyclopedia of DNA Elements (ENCODE) project from forebrain at E11.5, E12.5, and E14.5 to define active enhancers at these stages and searched for global conservation patterns using multi-vertebrate chain genome alignments. Most enhancers in the large potential regulatory landscapes of *Dbx1* and *Etv1* (155 Kbp and 1.7 Mbp, respectively) were detected in all studied jawed vertebrates (Figures S5 and S6), including a proximal regulatory region ~3.7 Kbp upstream of *Dbx1* that was previously shown to drive expression to the forebrain (Lu et al., 1996; Figure S5, blue box). However, we also observed several distal mammal-specific ATAC-seq peaks associated with both loci (Figures S5 and S6, red boxes), including a mammal-specific region linked to a vertebrate-specific highly conserved non-coding region in *Dbx1* (Figure S5, orange box). Finally, we also investigated the qualitative genomic conservation of two VISTA enhancers (hs636 and hs876), which have been reported to drive expression in the mouse VP (Pattabiraman et al., 2014). Interestingly, as in the case of the proximal *Dbx1* enhancer, these enhancers were found in all jawed vertebrates (Figure S7), suggesting that functional differences may be due to specific nucleotide substitutions that occurred in the mammalian lineage.

DISCUSSION

Several populations of cortical glutamatergic neurons critical for neocortical development are generated outside of the cortical neuroepithelium of the DP. We show here that none of these external populations migrate to the DP in the avian brain: Cajal-Retzius cells, subplate neurons, and transient pyramidal neurons. The novel arrival of these external cells may have been crucial in early cortical evolution because it could have influenced the developmental program of the mammalian DP at the level of neurogenesis, layering, and circuit formation.

Conserved Migration of Pallial Interneurons

The subpallial origin of pallial GABAergic interneurons has been extensively studied, and its homology has been proven in all extant vertebrates studied so far (Cobos et al., 2001a; Tuorto et al., 2003; Métin et al., 2007; Carrera et al., 2008; Moreno et al., 2008). However, most of the data have been limited to studies based on the expression of related transcription factors and gamma-aminobutyric acid (GABA). This approach fails to precisely identify the origin and progression of the relevant cell populations, which can only be investigated with cell lineage tracing methods. In chick, some pioneering studies were based on quail-to-chick heterotopic graft transplants (Cobos et al., 2001b) which, because they were performed very early in the embryonic period, could not discriminate the precise subpallial region of origin. Another study used *in vitro* slice cultures (Tuorto et al., 2003), but these offer restricted migratory alternatives to newborn neurons. Although the slice culture experiments pointed to a dorsal region of the subpallium as the origin of interneurons, our *in vivo* whole-brain tracing analysis proved that the ventral subpallium is the major source. Our full lineage tracing demonstrated that subpallial migration in the chick is homologous to the one observed in mammals because the same subpallial subsectors (pallidal) were found to be the main source of migratory interneurons in both taxa (Batista-Brito and Fishell, 2009). This part of the study also validated the experimental approach we used for other tangentially migrating glutamatergic neuronal populations.

The Chick DP Lacks a Homologous Tangentially Migrating Population of Preplate Neurons

In the early stages of cortical development, Cajal-Retzius cells and subplate neurons travel to the DP preplate (Aboitiz et al., 2005) from extra-neocortical sources before the genesis of the rest of cortical neurons (Barber and Pierani, 2016; García-Moreno et al., 2007; Pedraza et al., 2014). These early cell groups are also inherently transient because they die during early postnatal life after completion of their roles in cortical development. We showed here that none of these neurons migrate to the chick DP. To understand more precisely the evolutionary origin of these migrations, we need to study homologous populations in other outgroups, such as reptiles. Considering that the telencephalic structure of reptiles is less complex than that of both mammals and birds, we hypothesize that the reptilian brain also lacks a homolog of the preplate glutamatergic tangential migration. If this is the case, then it would indicate that the

amygdalofugal pathway and lateral forebrain bundle, K and L). The insets in (H), (I), (K), and (L) show the presence of labeled progenitors at the VP. The inset in (J) shows the lack of labeled cells in the rostral DP.

Thick dashed lines represent the PSB. Thin dashed lines establish the boundaries of the MsP. Empty arrowheads point to VP progenitors. DAPI counterstain is shown in blue. Scale bars represent 1 mm in (I); 500 μm in (F) for (F)–(I) and in (J) for (J)–(L); 250 μm in (C) and (E); and 100 μm in (D).

See also Figure S4.

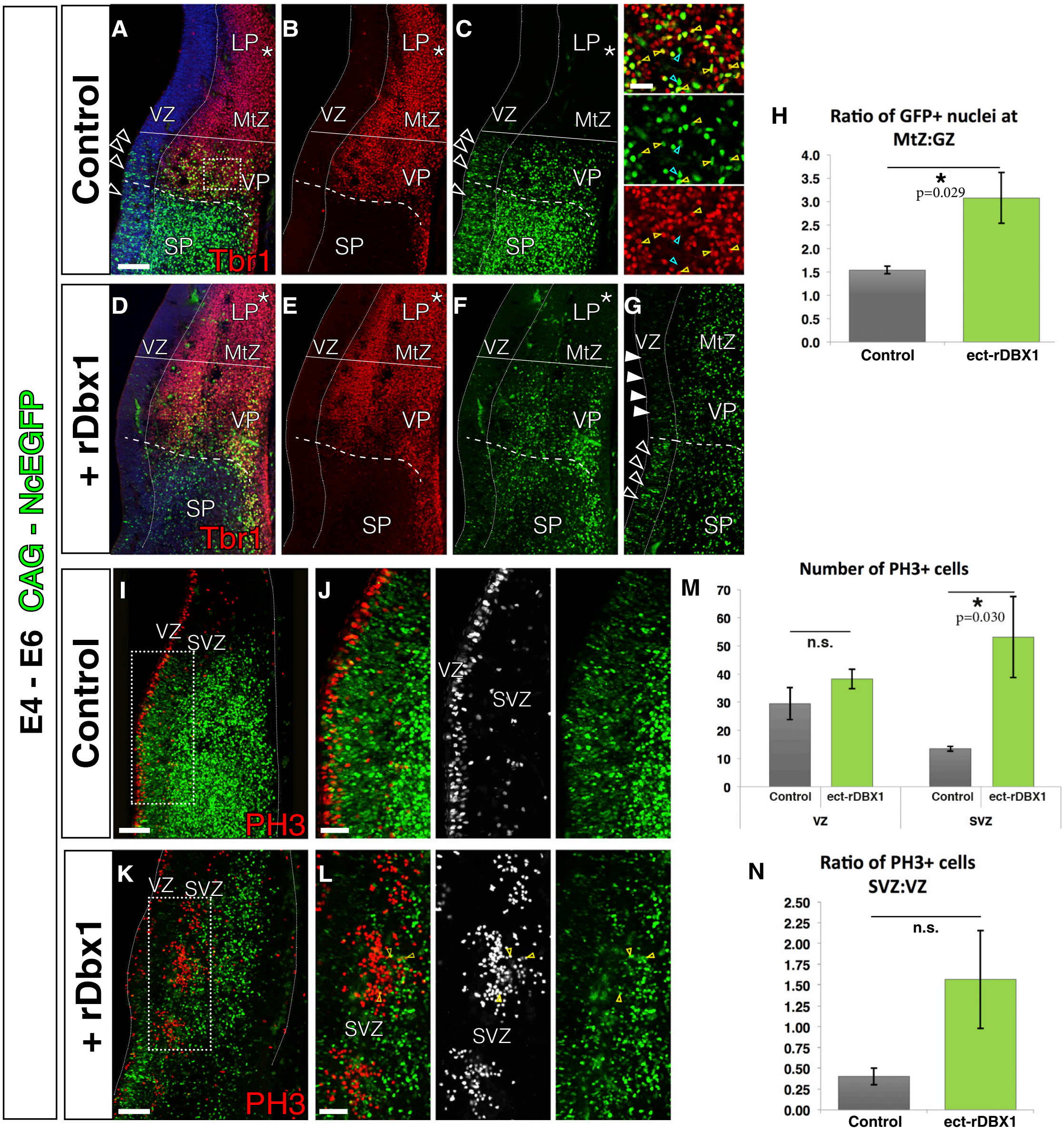

Figure 6. Ectopic rDbx1 Expression Modifies VP Neurogenesis but Does Not Induce Tangential Migration in the Chick Brain

Coronal sections are shown, medial at the left.

(A–F) Short-term ectopic rDbx1 expression electroporations of VP progenitors (D–F) produced, at E6, a radial progeny of VP-derived cells (n = 5), similar to control experiments (A–C, n = 4). No cells were found to migrate dorsally toward the LP or DP (asterisks). The Tbr1 pallial marker is shown in red; the inset in (C) shows many nuclear GFP-labeled cells derived from the VP that express Tbr1 (yellow arrowheads), whereas a minority derived from the subpallium do not express Tbr1 (cyan arrowheads).

(G) Two days after ect-rDBX1, VP GZ GFP expression was strongly diminished (arrowheads), whereas GFP was still clearly visible in SP electroporated progenitors (empty arrowheads).

(H) Ratio of GFP+ cells in the MtZ relative to the GZ (MtZ:GZ; 1,803 GFP+ ect-rDBX1 cells counted in n = 5; 1,113 GFP+ control cells counted in n = 4).

(I–L) Short-term ect-rDBX1 (K and L) promoted an increase in SVZ divisions with respect to the control experiment (I and J; control, n = 4, 588 PH3+ cells counted; ect-rDBX1, n = 5, 883 PH3+ cells counted). Mitotic nuclei were detected by immunohistochemistry for PH3 (in red, except in the center of J and L, in white). (J) and (L) are high power views of the rectangles shown in (I) and (K), respectively. Mitotic nuclei are arranged in clusters, but few mitotic cells derived from electroporated ect-rDBX1 electroporated progenitors (yellow arrowheads).

(M) Number of mitotic pH3+ cells in the GZs of the VP in control and ect-rDBX1 animals.

(N) Ratio of pH3+ cells in the subventricular zone relative to the ventricular zone (SVZ:VZ) in control and ect-rDBX1 experiments.

DAPI counterstain is shown in blue. Scale bars represent 100 μm in (I), (K), and (A) for (A)–(G); 50 μm in (J) and (L); and 25 μm in (A) in the inset.

Error bars correspond to SD. See also Figure S5.

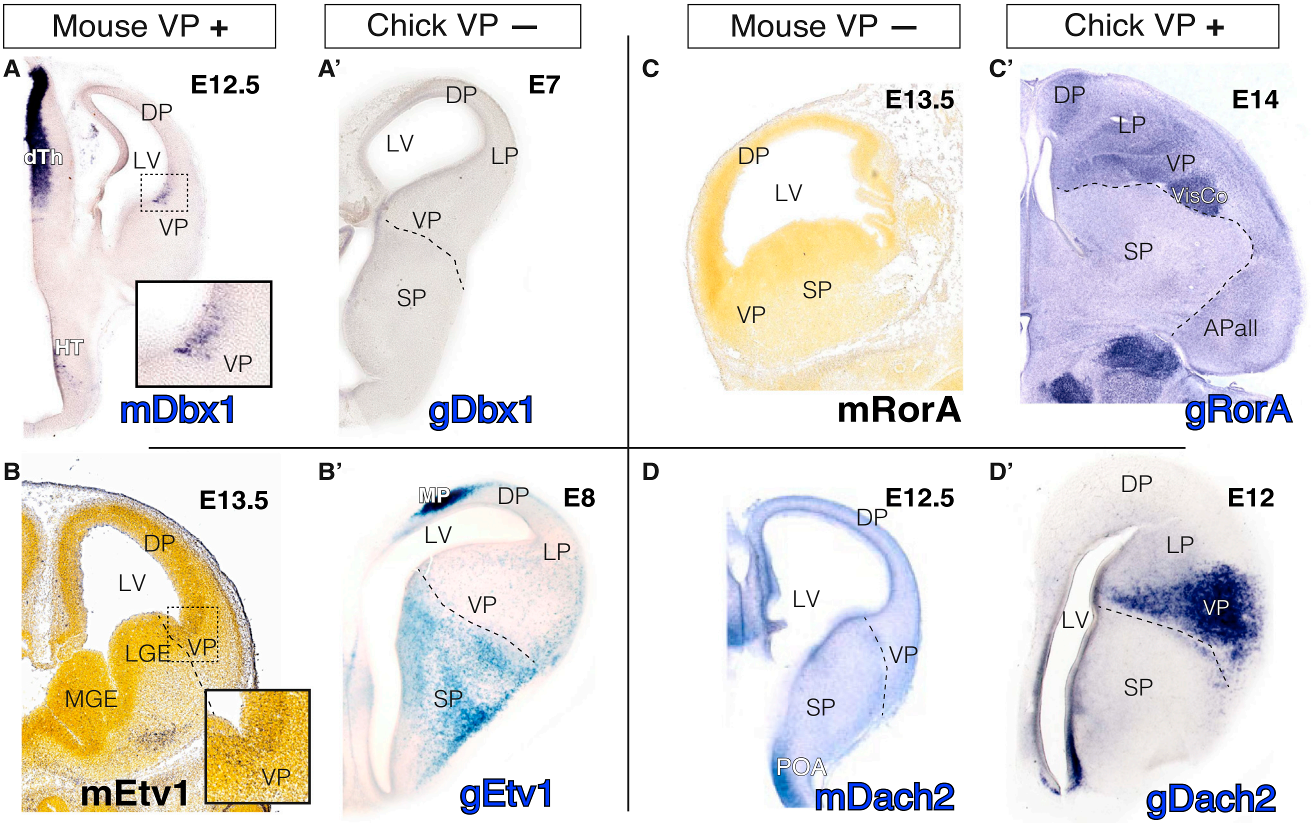

Figure 7. Genes Divergently Expressed in the VP of Mouse and Chick

Coronal sections are shown, medial at the left, except for (C), which shows a sagittal section, rostral at the left, and (C'), which shows a horizontal section, rostral at the top.

(A–D') Expression data from *in situ* hybridization on mouse (A–D) and chick (A'–D') embryonic specimens at neurogenic stages as detailed in the figure.

(A–B') Dbx1 and Etv1 are expressed in the early murine VP but not in the chick homologous region.

(A) Mouse Dbx1.

(A') Chick Dbx1.

(B) Mouse Etv1.

(B') Chick Etv1.

(C–D') RorA and Dach2 show an opposite expression pattern and are expressed in the chick VP but not in the mouse.

(C) Mouse RorA.

(C') Chick RorA.

(D) Mouse Dach2.

(D') Chick Dach2.

See also Figures S5–S7.

pallio-pallial tangential glutamatergic migrations observed in mammalian brains are a mammalian innovation, whereas the GABAergic tangential migrations are highly conserved (Métin et al., 2007).

It has been suggested, based on molecular profiling, that homolog populations of Cajal-Retzius cells and subplate cells exist in avian brains (Bernier et al., 2000; Wang et al., 2011). However, in light of our findings, the developmental origins of these cells were not conserved. Regarding Cajal-Retzius cell migration in sauropsids, a previous study investigated the developmental origin of avian reelin-expressing pallial neurons (Nomura et al., 2008). Although the authors claim that the quail cortical hem gives rise to tangentially migrating Cajal-Retzius cells, the broad tracing performed could not resolve the precise origin of the migratory population described (see their Figure 2). Alternatively, it is possible that those EGFP+ cells in quail experiments may migrate ventrally from the DP. This is a behavior we have also observed in our experiments, and it is in accordance with what is described in the mouse (García-Moreno et al., 2008). In addition, another study using chick pallial lineage tracing experiments showed results identical to ours (see Figure S2 in Suzuki et al., 2012): a lack of tangential migration from pallial sources.

The Evolutionary Role of Dbx1: Shaping the Ventral and Dorsal Pallia

Our data show that avian VP progenitors differ widely in their neurogenic behavior from their mammalian homologs. Avian VP progenitors produce a relatively larger lineage, as made clear

when comparing the avian DVR with the mammalian piriform cortex and pallial portion of the amygdala (Puelles et al., 2016). Furthermore, avian VP progenitors did not produce cells contributing to the DP. Our data point to a partial function for Dbx1 in the generation of this VP divergence. Ectopic expression of rDbx1 in chick VP progenitors led to more differentiative mitoses, reducing the VZ progenitor pool over time, and increased proliferation in the SVZ because of a non-cell-autonomous effect, which must be transient because SVZ progenitors can only divide a limited number of times (Noctor et al., 2004). Together, these two changes, triggered by expression of Dbx1, led to a reduction of the VP progeny. Altogether, it is likely that *de novo* expression of Dbx1 in mammalian VP progenitors elicited a reduction in VP size, which could have allowed for the relative expansion of the DP.

We show here a crucial migratory divergence in amniotes species. However, this is not driven by the differential expression of Dbx1, as we hypothesized. We found only a handful of transcription factor genes that are differentially expressed in the early pallium of amniotes. Our comparative study of potential regulatory regions further identified several mammal-specific regions associated with *Dbx1* and *Etv1*, which could be responsible, at least in part, for their novel expression in the mammalian VP. However, several other previously characterized enhancers that drive expression in the mouse VP (including those in Dbx1 and VISTA hs636 and hs876 elements) are present in all vertebrates. Enhancer reporter assays of the mammalian and chicken sequences in both systems may shed light onto whether the differences in tangential migration of VP neuroblasts are due to nucleotide substitutions in one lineage (*cis* changes) or to distinct regulatory states in the mouse and chick VP (*trans* changes) (Davidson, 2006).

Tangential Migration as a Source of Evolutionary Divergence

Tangentially migrating cells are generated in distant GZs with different transcriptional control. Therefore, the morphological, hodological, and neurochemical features of the cells arriving through tangential migration differ from those of the locally born cells. Tangentially migrating glutamatergic cells orchestrate the early circuit assembly at their fate region, as GABAergic interneurons do in the neocortex. Therefore, tangential migration undoubtedly increases the cell diversity, circuit complexity, and computational capabilities of any given brain area. If there is an evolutionary divergence on neuronal tangential migration, then it could be translated into a selective advantage.

In the case of transient cortical populations, the divergence created by tangential migration is not restricted to the complex elaboration of circuitry. These cells also play critical roles during cortical development (Puelles, 2011). The neurogenesis-related functions of Cajal-Retzius cells (Ogawa et al., 1995; Supèr et al., 2000) and the control of early circuitry by subplate cells (Hoerder-Suabedissen and Molnár, 2015) are essential platforms for the subsequent developmental program of the mammalian DP. Without these transient cell groups, the mammalian neocortex would be structured very differently. Therefore, we can speculate that the novel arrival of these cells triggered a divergent plan of DP development. This divergence may have sculpted the mammalian DP by promoting its layering, increased production of neurons, and columnar circuit assembly. The development of all known mammalian neocortical circuitry is centered on subplate and Cajal-Retzius neurons. Such relevant divergences lead us to suggest that novel external pallial contributions to the ancient mammalian DP may have been a key factor in the origin of the current mammalian neocortex.

EXPERIMENTAL PROCEDURES

Animals

All animal experiments were approved by a local ethical review committee and conducted in accordance with personal and project licenses under the UK Animals (Scientific Procedures) Act (1986) and in compliance with the current normative standards of the European Union (Directive 2010/63/EU) and the Spanish Government (Royal Decrees 1201/2005 and 53/2013, Law 32/107). Details regarding experimental chick and mice can be found in the Supplemental Experimental Procedures.

In Ovo Electroporation and Tracing

Electroporation of chick embryos was performed as described previously (García-Moreno et al., 2014). For targeted electroporations of focal telencephalic areas, the positive pole was placed next to the telencephalic areas to be electroporated, whereas the negative electrode was positioned at the opposite brain site. Here we exploit the *piggybac* (PB) transposase system (Ding et al., 2005), which delivers transgenes into the genome of the transfected cells. PB turns on the expression of the reporter gene permanently both in the electroporated stem cells and their whole progeny (García-Moreno et al., 2014). In this work, we electroporated chick embryos *in ovo* at the onset of telencephalic neurogenesis on E4, Hamburger and Hamilton (HH) stage 23–24 (Hamburger and Hamilton, 1951). Details regarding *in ovo* electroporation can be found in the Supplemental Experimental Procedures.

In other experiments, FDA (molecular weight [MW], 3,000) was injected into the embryonic neuroepithelium (E4). This way we obtained more precise, although not permanent, labeling of progenitors (García-Moreno et al., 2008; Métin et al., 2007).

In Utero Electroporation

Transfection by electroporation of embryonic neural progenitors was performed as described previously (García-Moreno et al., 2010).

Plasmids

Most of the plasmid constructs employed in this study were also employed in a previous study (García-Moreno and Molnár, 2015), which describes these in detail. Details regarding the plasmids employed can be found in the Supplemental Experimental Procedures.

Tissue Processing and Immunohistochemistry

Mice and chick embryo fixation and perfusion, tissue processing, and immunohistochemical analysis were performed as described previously (García-Moreno and Molnár, 2015); details can be found in the Supplemental Experimental Procedures.

SUPPLEMENTAL INFORMATION

Supplemental Information includes Supplemental Experimental Procedures and seven figures and can be found with this article online at https://doi.org/10.1016/j.celrep.2017.12.032.

ACKNOWLEDGMENTS

We thank Prof. Luis Puelles and Prof. José Luis Ferrán (University of Murcia, Spain) for providing images of gene expression patterns and invaluable discussions regarding the project; Dr. Navneet Vasistha (University of Edinburgh), Dr. Luiz Guidi (University of Oxford), Dr. Isabel Martínez-Garay (Cardiff

University), and Prof. John Parnavelas (University College London) for helpful manuscript revision; Prof. Nomura (Kyoto Prefectural University, Japan) for constructive comments regarding the design of the project and interpretation of results; Prof. Nakagawa (University of Minnesota, USA) for kindly providing the Dbx1 antibody; and Panos Firbas (CABD-CSIC, Spain) for providing ATAC-seq mappings and peak callings. F.G.-M. was supported by Human Frontiers Science Program Long-Term Postdoctoral Fellowship Program LT000618/2011-L and currently holds an IKERBASQUE research fellowship. Z.M. holds research grants from the Biotechnology and Biological Sciences Research Council, the Wellcome Trust, and the Medical Research Council UK. M.I. is supported by grants from the European Research Council (ERC-StG-LS2-637591) and the Spanish Ministry of Economy and Competitiveness (BFU2014-55076-P to M.I. and "Centro de Excelencia Severo Ochoa 2013-2017" and SEV-2012-0208 to the Centre for Genomic Regulation [CRG]). We acknowledge the support of the CERCA Programme/Generalitat de Catalunya. J.M.E. holds MINECO SAF-2015-70866-R (with FEDER funds) and RyC-2012-11137 grants.

AUTHOR CONTRIBUTIONS

Conceptualization, F.G.-M. and Z.M.; Methodology and Investigation, F.G.-M., E.A., M.J., and M.I.; Resources and Funding Acquisition, J.B., J.M.E., and Z.M.; Writing – Original Draft and Visualization, F.G.-M.; Writing – Review & Editing, F.G.-M., E.A., M.J., J.B., J.M.E., M.I., and Z.M.; Supervision, F.G.-M. and Z.M.

DECLARATION OF INTERESTS

The authors declare no competing interests.

Received: December 23, 2016
Revised: July 29, 2017
Accepted: December 8, 2017
Published: January 2, 2018

REFERENCES

Abellán, A., and Medina, L. (2009). Subdivisions and derivatives of the chicken subpallium based on expression of LIM and other regulatory genes and markers of neuron subpopulations during development. J. Comp. Neurol. *515*, 465–501.

Abellán, A., Desfilis, E., and Medina, L. (2014). Combinatorial expression of Lef1, Lhx2, Lhx5, Lhx9, Lmo3, Lmo4, and Prox1 helps to identify comparable subdivisions in the developing hippocampal formation of mouse and chicken. Front. Neuroanat *8*, 1–22.

Aboitiz, F., Montiel, J., and García, R.R. (2005). Ancestry of the mammalian preplate and its derivatives: evolutionary relicts or embryonic adaptations? Rev. Neurosci. *16*, 359–376.

Anderson, S.A., Eisenstat, D.D., Shi, L., and Rubenstein, J.L. (1997). Interneuron migration from basal forebrain to neocortex: dependence on Dlx genes. Science *278*, 474–476.

Barber, M., and Pierani, A. (2016). Tangential migration of glutamatergic neurons and cortical patterning during development: Lessons from Cajal-Retzius cells. Dev. Neurobiol. *76*, 847–881.

Batista-Brito, R., and Fishell, G. (2009). The Developmental Integration of Cortical Interneurons into a Functional Network. In Current Topics in Developmental Biology, Current Topics in Developmental Biology, Chapter 3, O. Hobert, ed. (Elsevier), pp. 81–118.

Beffert, U., Weeber, E.J., Morfini, G., Ko, J., Brady, S.T., Tsai, L.-H., Sweatt, J.D., and Herz, J. (2004). Reelin and cyclin-dependent kinase 5-dependent signals cooperate in regulating neuronal migration and synaptic transmission. J. Neurosci. *24*, 1897–1906.

Bernier, B., Bar, I., D'Arcangelo, G., Curran, T., and Goffinet, A.M. (2000). Reelin mRNA expression during embryonic brain development in the chick. J. Comp. Neurol. *422*, 448–463.

Bielle, F., Griveau, A., Narboux-Nême, N., Vigneau, S., Sigrist, M., Arber, S., Wassef, M., and Pierani, A. (2005). Multiple origins of Cajal-Retzius cells at the borders of the developing pallium. Nat. Neurosci. *8*, 1002–1012.

Carrera, I., Ferreiro-Galve, S., Sueiro, C., Anadón, R., and Rodríguez-Moldes, I. (2008). Tangentially migrating GABAergic cells of subpallial origin invade massively the pallium in developing sharks. Brain Res. Bull. *75*, 405–409.

Cobos, I., Puelles, L., and Martínez, S. (2001a). The avian telencephalic subpallium originates inhibitory neurons that invade tangentially the pallium (dorsal ventricular ridge and cortical areas). Dev. Biol. *239*, 30–45.

Cobos, I., Shimamura, K., Rubenstein, J.L.R., Martínez, S., and Puelles, L. (2001b). Fate map of the avian anterior forebrain at the four-somite stage, based on the analysis of quail-chick chimeras. Dev. Biol. *239*, 46–67.

Davidson, E.H. (2006). The Regulatory Genome: Gene Regulatory Networks in Development and Evolution (Academic Press).

de Carlos, J.A.J., López-Mascaraque, L., and Valverde, F. (1996). Dynamics of cell migration from the lateral ganglionic eminence in the rat. J. Neurosci. *16*, 6146–6156.

Ding, S., Wu, X., Li, G., Han, M., Zhuang, Y., and Xu, T. (2005). Efficient transposition of the piggyBac (PB) transposon in mammalian cells and mice. Cell *122*, 473–483.

García-Moreno, F., and Molnár, Z. (2015). Subset of early radial glial progenitors that contribute to the development of callosal neurons is absent from avian brain. Proc. Natl. Acad. Sci. USA *112*, E5058–E5067.

García-Moreno, F., López-Mascaraque, L., and De Carlos, J.A. (2007). Origins and migratory routes of murine Cajal-Retzius cells. J. Comp. Neurol. *500*, 419–432.

García-Moreno, F., López-Mascaraque, L., and de Carlos, J.A. (2008). Early telencephalic migration topographically converging in the olfactory cortex. Cereb. Cortex *18*, 1239–1252.

García-Moreno, F., Pedraza, M., Di Giovannantonio, L.G., Di Salvio, M., López-Mascaraque, L., Simeone, A., and De Carlos, J.A. (2010). A neuronal migratory pathway crossing from diencephalon to telencephalon populates amygdala nuclei. Nat. Neurosci. *13*, 680–689.

García-Moreno, F., Vasistha, N.A., Begbie, J., and Molnár, Z. (2014). CLoNe is a new method to target single progenitors and study their progeny in mouse and chick. Development *141*, 1589–1598.

Geschwind, D.H., and Rakic, P. (2013). Cortical evolution: judge the brain by its cover. Neuron *80*, 633–647.

Gould, S.J. (1977). Ontogeny and Phylogeny (Harvard University Press).

Grove, E.A., Tole, S., Limon, J., Yip, L., and Ragsdale, C.W. (1998). The hem of the embryonic cerebral cortex is defined by the expression of multiple Wnt genes and is compromised in Gli3-deficient mice. Development *125*, 2315–2325.

Hamburger, V., and Hamilton, H.L. (1951). A series of normal stages in the development of the chick embryo. J. Morphol. *88*, 49–92.

Hoerder-Suabedissen, A., and Molnár, Z. (2015). Development, evolution and pathology of neocortical subplate neurons. Nat. Rev. Neurosci *16*, 133–146.

Imayoshi, I., Shimogori, T., Ohtsuka, T., and Kageyama, R. (2008). Hes genes and neurogenin regulate non-neural versus neural fate specification in the dorsal telencephalic midline. Development *135*, 2531–2541.

Jarvis, E.D., Yu, J., Rivas, M.V., Horita, H., Feenders, G., Whitney, O., Jarvis, S.C., Jarvis, E.R., Kubikova, L., Puck, A.E.P., et al. (2013). Global view of the functional molecular organization of the avian cerebrum: mirror images and functional columns. J. Comp. Neurol. *521*, 3614–3665.

Lu, S., Shashikant, C.S., and Ruddle, F.H. (1996). Separate cis-acting elements determine the expression of mouse Dbx gene in multiple spatial domains of the central nervous system. Mech. Dev. *58*, 193–202.

Marín, O. (2013). Cellular and molecular mechanisms controlling the migration of neocortical interneurons. Eur. J. Neurosci. *38*, 2019–2029.

Métin, C., Alvarez, C., Moudoux, D., Vitalis, T., Pieau, C., and Molnár, Z. (2007). Conserved pattern of tangential neuronal migration during forebrain development. Development *134*, 2815–2827.

Moreno, N., González, A., and Rétaux, S. (2008). Evidences for tangential migrations in Xenopus telencephalon: developmental patterns and cell tracking experiments. Dev. Neurobiol. *68*, 504–520.

Noctor, S.C., Martínez-Cerdeño, V., Ivic, L., and Kriegstein, A.R. (2004). Cortical neurons arise in symmetric and asymmetric division zones and migrate through specific phases. Nat. Neurosci. *7*, 136–144.

Nomura, T., Takahashi, M., Hara, Y., and Osumi, N. (2008). Patterns of neurogenesis and amplitude of Reelin expression are essential for making a mammalian-type cortex. PLoS ONE *3*, e1454.

Ogawa, M., Miyata, T., Nakajima, K., Yagyu, K., Seike, M., Ikenaka, K., Yamamoto, H., and Mikoshiba, K. (1995). The reeler gene-associated antigen on Cajal-Retzius neurons is a crucial molecule for laminar organization of cortical neurons. Neuron *14*, 899–912.

Pattabiraman, K., Golonzhka, O., Lindtner, S., Nord, A.S., Taher, L., Hoch, R., Silberberg, S.N., Zhang, D., Chen, B., Zeng, H., et al. (2014). Transcriptional regulation of enhancers active in protodomains of the developing cerebral cortex. Neuron *82*, 989–1003.

Pedraza, M., Hoerder-Suabedissen, A., Albert-Maestro, M.A., Molnár, Z., and De Carlos, J.A. (2014). Extracortical origin of some murine subplate cell populations. Proc. Natl. Acad. Sci. USA *111*, 8613–8618.

Puelles, L. (2011). Pallio-pallial tangential migrations and growth signaling: new scenario for cortical evolution? Brain Behav. Evol. *78*, 108–127.

Puelles, L., Kuwana, E., Puelles, E., Bulfone, A., Shimamura, K., Keleher, J., Smiga, S., and Rubenstein, J.L. (2000). Pallial and subpallial derivatives in the embryonic chick and mouse telencephalon, traced by the expression of the genes Dlx-2, Emx-1, Nkx-2.1, Pax-6, and Tbr-1. J. Comp. Neurol. *424*, 409–438.

Puelles, L., Medina, L., Borello, U., Legaz, I., Teissier, A., Pierani, A., and Rubenstein, J.L.R. (2016). Radial derivatives of the mouse ventral pallium traced with Dbx1-LacZ reporters. J. Chem. Neuroanat. *75* (*Pt A*), 2–19.

Rakic, P. (1971). Guidance of neurons migrating to the fetal monkey neocortex. Brain Res. *33*, 471–476.

Reiner, A., Yamamoto, K., and Karten, H.J. (2005). Organization and evolution of the avian forebrain. Anat. Rec. A Discov. Mol. Cell. Evol. Biol. *287*, 1080–1102.

Supèr, H., Del Río, J.A., Martínez, A., Pérez-Sust, P., and Soriano, E. (2000). Disruption of neuronal migration and radial glia in the developing cerebral cortex following ablation of Cajal-Retzius cells. Cereb. Cortex *10*, 602–613.

Suzuki, I.K., and Hirata, T. (2014). A common developmental plan for neocortical gene-expressing neurons in the pallium of the domestic chicken Gallus gallus domesticus and the Chinese softshell turtle Pelodiscus sinensis. Front. Neuroanat *8*, 1–17.

Suzuki, I.K., Kawasaki, T., Gojobori, T., and Hirata, T. (2012). The temporal sequence of the mammalian neocortical neurogenetic program drives mediolateral pattern in the chick pallium. Dev. Cell *22*, 863–870.

Teissier, A., Griveau, A., Vigier, L., Piolot, T., Borello, U., and Pierani, A. (2010). A novel transient glutamatergic population migrating from the pallial-subpallial boundary contributes to neocortical development. J. Neurosci. *30*, 10563–10574.

Tremblay, R., Lee, S., and Rudy, B. (2016). GABAergic Interneurons in the Neocortex: From Cellular Properties to Circuits. Neuron *91*, 260–292.

Tuorto, F., Alifragis, P., Failla, V., Parnavelas, J.G.J., and Gulisano, M. (2003). Tangential migration of cells from the basal to the dorsal telencephalic regions in the chick. Eur. J. Neurosci. *18*, 3388–3393.

Wang, W.-Z., Oeschger, F.M., Montiel, J.F., García-Moreno, F., Hoerder-Suabedissen, A., Krubitzer, L., Ek, C.J., Saunders, N.R., Reim, K., Villalón, A., and Molnár, Z. (2011). Comparative aspects of subplate zone studied with gene expression in sauropsids and mammals. Cereb. Cortex *21*, 2187–2203.

Cell Reports, Volume *22*

Supplemental Information

Absence of Tangentially Migrating Glutamatergic Neurons in the Developing Avian Brain

Fernando García-Moreno, Edward Anderton, Marta Jankowska, Jo Begbie, Juan Manuel Encinas, Manuel Irimia, and Zoltán Molnár

Supplementary Experimental Procedures

Animals

All animal experiments were approved by a local ethical review committee and conducted in accordance with personal and project licenses under the UK Animals (Scientific Procedures) Act (1986), and were conducted in compliance with the current normative standards of the European Union (Directive 2010/63/EU) and the Spanish Government (Royal Decree 1201/2005 and 53/2013; Law 32/107). Fertilized hen eggs, obtained from Winter Egg Farm (UK), were incubated at 38 °C in humidified atmosphere until required stages (Haumburger and Hamilton, 1951). The day when eggs were incubated was considered E0. Adult C57BL/6 mice were obtained from a local breeding colony at the University of Oxford (based on the Harlan (UK) strain). These were maintained on a 12/12-hour light/dark cycle (7 AM, lights on) and provided with ad libitum access to food and water. The day when vaginal plug was detected was referred to as E0. Embryos from both genders were analyzed at different developmental stages, as stated in the figures.

In ovo electroporation and tracing

The main purpose of our research is to describe comparable glutamatergic tangential migrations during chick telencephalic development. For this purpose, our electroporation method for labeling neural progenitors is likely the most powerful methodology. With this method, cells are enabled to migrate in vivo, inside the intact whole brain. It shows a direct relationship between the cells' origin in the VZ and their fate within the tissue days later. Our electroporation technique is able to show (1) whether the migration of telencephalic neurons actually happens, (2) the migratory route of these neurons, (3) the distribution of them after migration and (4) their identity. However, electroporation also shows some limitations. The number and location of progenitors transfected ranges from one animal to another in a way impossible to keep under control.

Electroporation of chick embryos was performed as previously described (Garcia-Moreno et al., 2014). For targeted electroporations of focal telencephalic areas the positive pole was placed next to the telencephalic areas to be electroporated, whereas the positive electrode was positioned at the opposite brain site.

Embryonic electroporation of reporter genes has been widely used to describe the lineage of neural stem cells. Here we exploit the piggybac (PB) transposase system (Ding et al., 2005) that delivers transgenes into the genome of the transfected cells. PB turns on the expression of the reporter gene permanently both in the electroporated stem cells and their whole progeny (Garcia-Moreno et al., 2014). In this work we electroporated chick embryos in ovo at the onset of telencephalic neurogenesis at E4, Hamburger and Hamilton (HH) stage 23-24 (Haumburger and Hamilton, 1951). We later investigated the labeled lineage at different time points, short-term tracing between E6-E8 (HH29-34) to observe the cells during the migratory process, and between E10-E14 (HH36-40) to describe the definitive long-term neuronal progeny at the end of telencephalic neurogenesis. The transposable system employed here offers two notable improvements for the study of cellular migration. First, as it labels stem progenitors permanently, these are always detectable. Therefore we can define the telencephalic identity of the specific ventricular sector where the electroporation was performed. And secondly, since the whole progeny inherits the genomic label from the progenitor, cell populations generated late after electroporation are also detectable, as opposed to traditional non-transposable electroporation. Moreover, this methodology enables native three-dimensional cellular trajectories in the whole intact live brain.

In other experiments, fluorescein dextran amine (3000MW) was injected within the embryonic neuroepithelium (E4). This way we obtained a more precise although not permanent labeling of progenitors (Garcia-Moreno et al., 2008; Metin et al., 2007).

In utero electroporation

Transfection by electroporation of embryonic neural progenitors was performed as described previously (Garcia-Moreno et al., 2010).

Plasmids

Most of the plasmid constructs employed in this study were employed in a previous study (Garcia-Moreno and Molnár, 2015), which describes these in detail. The concentration of different plasmids was kept constant among the different experiments in chick and mouse embryos. Labeling constructs (pPB-CAG-EYFP; pPB-CAG-MbEGFP) were transfected at a final concentration ranging between 200-500 ng/µl; the transposase enzyme expressing-construct (mPB) was consistently transfected at 300 ng/µl.

Tissue processing

Embryonic murine brains and chick embryonic brains up to E9 were fixed by immersion in 4% paraformaldehyde (PFA, diluted in phosphate buffered saline 0.1M– PBS, pH 7.3), whereas E10-E14 chick embryos were transcardially perfused with PBS followed by PFA. Brains were transferred to PBS 24h after fixation. All brains were sectioned in either the coronal or horizontal planes at 50-70 µm thickness in a vibrating microtome (Leica VT1000S).

Immunohistochemistry

Single and dual immunohistochemical reactions were performed as described previously (Garcia-Moreno et al., 2012) using the following primary antibodies: Rabbit antibody to EGFP (Molecular Probes, A11122, 1:1000), mouse antibody to EGFP (Abcam, ab1218, 1:1000), chick antibody to EGFP (Aves GFP 1020, 1:10000), rabbit anti calbindinD-28 (Swant, CB38, 1:10000), rabbit anti calretinin (Swant, 7697, 1:2000), rabbit antibody to Dbx1 (1:200; kind gift by Prof. Nakagawa, Univ. Minessota, US), rabbit antibody to PH3 (Chemicon, 06-570, 1:1,000), rabbit antibody to Tbr1 (Chemicon, AB9616, 1:1,000), rat antibody to Ctip2 (Abcam, ab18465, 1:500), rabbit antibody to dsRed2 (Takara, 632475, 1:1000), mouse antibody to Islet1 (DSHB, 39.4D5, 1:500) AND mouse antibody to Satb2 (Abcam, ab51502, 1:500). Dbx1 immunostaining required antigen retrieval with citrate acid.

For secondary antibodies (all 1:1000), we used Alexa 568 goat antibody to rabbit IgG (Molecular Probes, A11011), Alexa 647 goat antibody to rabbit IgG (Molecular Probes, A21245), Alexa 488 goat antibody to rabbit IgG (Molecular Probes, A11034), Alexa 488 goat antibody to mouse IgG (Molecular Probes, A11001), Alexa 568 antibody to mouse IgG (Molecular Probes, A11004), Alexa 568 goat antibody to rat IgG (Molecular Probes, A11077), and Alexa 488 goat antibody to chicken (Invitrogen, A11039,).

In situ hybridization

M13forward (5´GCCAGGGTTTTCCCAGTCAC3´) and M13reverse (5´GGAAACAGCTATGACCATG3´) primers were used to obtain the fragments from our cloned genes that were used to get riboprobes. Sense and antisense digoxygenin-11-UTP-labeled (Roche, Lewes, United Kingdom) riboprobes were synthesized according to the detailed procedures

previously described (Ferran et al., 2015). All the steps and procedures related with the extraction of brain samples, tissue processing and in situ hybridization in cryostat and floating sections were done as previously described (Ferran et al., 2015).

In silico analysis of potential regulatory elements

ATAC-seq data was downloaded from the ENCODE portal. Data for mouse forebrain at E11.5, E12.5 and E14.5 (ENCSR273UFV, ENCSR559FAJ and ENCSR810HQR, respectively; two replicates each) was used for the analysis. Raw ATAC-seq reads were mapped to the mouse genome (mm10 assembly) using bowtie2 (Langmead and Salzberg 2012) with the following options: --very-sensitive -X 2000 -I 0. Read pairs with a fragment length < 120bp were considered "nucleosome free" and subsequently used for peak calling. For this, MACS was used for low-thresholded peak calling using the following parameters: callpeak --nomodel --keep-dup 1 --llocal 10000 --extsize 74 --shift -37 -p 0.07 (middle track in UCSC browser screenshots). Next, the IDR framework (idr 0.1; https://github.com/nboley/idr) was used to determine high confidence peaks based on replicate information (bottom track; IDR). Density of ATAC-seq reads was displayed from bigwig files (top track).

To evaluate evolutionary conservation at a qualitative level, we used the PhyloP and conservation tracks from the UCSC browser. For the latter, we selected the following species: rat, rabbit, human, rhesus, cow, dog, elephant (Eutherian mammals); opossum, platypus (non-Eutherian mammals); chicken, zebra finch, painted turtle, xenopus tropicalis, coelacanth and zebrafish (non-mammalian verebrates). A putative enhancer (ATAC-seq IDR peaks in the three stages) was considered "mammalian-specific" if present in most Eutherian mammals (inferred to be present in the last common ancestor of Eutherians) but absent in all non-mammalian vertebrates.

Imaging and analysis

Images were captured using a Zeiss LSM 710 confocal microscope (Carl Zeiss Microimaging). Similar image parameters (laser power, gain, pinhole and wavelengths) were maintained for images from each brain and adjusted for new specimens. The employed fluorophores were DAPI, EGFP, Alexa 488, mCherry, Alexa 568 and Alexa 647. In selected cases, Z-stacks were taken individually for each channel and then collapsed to get maximum intensity projections. For panoramic views of big brain sections, tile-scan images were composed. Images were adjusted and analyzed using ImageJ (Image Analysis in Java, NIH) and Adobe Photoshop CS6 (Adobe Systems Inc.).

Images were taken from a minimum of 3 animals successfully electroporated in each pallial region (specific numbers for each experimental paradigms are included into the relevant figure legends). In addition to these numbers, dozens of other animals with broader electroporations were also analyzed and taken into consideration for the interpretations of results. This study is based on over 300 hundred chicken embryos electroporated and analyzed.

The lack of migratory cells arriving to DP from the different pallial sources did not need to be quantified, as there were no cells migrating to DP. Cells were counted on single confocal planes. Nuclear-tagged GFP made the counting more reliable. A minimum of three representative sections was taken into account to estimate the average number of cells per animal. Control and ect-rDBX1 animals were compared by t-Student test (significance $p<0.05$).

Abbreviations

The atlas "The chick brain in stereotaxic coordinates" by Puelles et al. (2007) primarily considers the Avian Brain Nomenclature Forum (Reiner et al., 2004), but it also applies a major developmental point of view over controversial regions. Having a developmental focus in our study, we have followed this atlas for our anatomical nomenclature in chick telencephalon.

Ac - anterior commissure

Ag - Amygdala

AuNP - Auditory nidopallium

BSS - Basal somatosensory nucleus of the nidopallium

cDVR - Caudal portion of the dorsal ventricular ridge

Cl - Claustrum

Cng - Cingulate cortex

cNP - Caudal region of the nidopallium

CP - Cortical plate

C-R - Cajal-Retzius cells

DP - Dorsal pallium

DVR - Dorsal ventricular ridge

GP - Globus pallidus

Hc - Hippocampus

HD -Densocellular hyperpallium

Hem -Cortical hem

Hyp - Hyperpallium

Ins - Insular cortex

IZ - Intermediate zone

L5 - Cortical layer 5

L6 - Cortical layer 6

lfb - Lateral forebrain bundle

LSt - Lateral striatum

LV - Lateral ventricle

MP - Medial pallium

MsP - Mesopallium

MSt - Medial striatum

MZ - Marginal zone

MtZ - Mantle zone

NP - Nidopallium

OB - Olfactory bulb

P - Pallium

PHA - Parahippocampal area

PO - Preoptic area

Pir - Piriform cortex

RMTW - Rostral medial telencephalic wall

Se - Septum

sNP - Superficial nidopallium

SP - Subpallium

St - Striatum

SVZ - Subventricular zone

TE - Thalamic eminence

VMS - Ventropallial migratory stream

VisCo - Visual nidopallial nucleus

vo - Ventral olfactory tract

VP - Ventral pallium

VZ - Ventricular zone

Supplementary Reference list

Ding, S., Wu, X., Li, G., Han, M., Zhuang, Y., Xu, T., 2005. Efficient Transposition of the piggyBac (PB) Transposon in Mammalian Cells and Mice. Cell 122, 473–483. doi:10.1016/j.cell.2005.07.013

Ferran, J.L., Ayad, A., Merchán, P., Morales-Delgado, N., Sánchez-Arrones, L., Alonso, A., SandovalSandoval, J.E., Bardet, S.M., Corral-San-Miguel, R., Sánchez-Guardado, L.Ó., Hidalgo-Sánchez, M., Martínez-de-la-Torre, M., Puelles, L., 2015. Exploring Brain Genoarchitecture by Single and Double Chromogenic In Situ Hybridization (ISH) and Immunohistochemistry (IHC) on Cryostat, Paraffin, or Floating Sections, in: Neuromethods, Neuromethods. Springer New York, New York, NY, pp. 83–107. doi:10.1007/978-1-4939-2303-8_5

Garcia-Moreno, F., López-Mascaraque, L., De Carlos, J.A., 2008. Early telencephalic migration topographically converging in the olfactory cortex. Cereb Cortex 18, 1239–1252. doi:10.1093/cercor/bhm154

Garcia-Moreno, F., Pedraza, M., Di Giovannantonio, L.G., Di Salvio, M., López-Mascaraque, L., Simeone, A., De Carlos, J.A., 2010. A neuronal migratory pathway crossing from diencephalon to telencephalon populates amygdala nuclei. Nat Neurosci 13, 680–689. doi:10.1038/nn.2556

Garcia-Moreno, F., Vasistha, N.A., Begbie, J., Molnár, Z., 2014. CLoNe is a new method to target single progenitors and study their progeny in mouse and chick. Development 141, 1589–1598. doi:10.1242/dev.105254

Hamburger, V., and Hamilton, H.L., 1951. A series of normal stages in the development of the chick embryo. J Morphol 88, 49–92.Metin et al., 2007

Puelles, L., Martinez de la Torre, M., Watson, C., Paxinos, G., Martinez, S., 2007. The Chick Brain in Stereotaxic Coordinates: An Atlas Featuring Neuromeric Subdivisions and Mammalian Homologies. Academic Press

Reiner, A., Perkel, D.J., Bruce, L.L., Butler, A.B., Csillag, A., Kuenzel, W., Medina, L., Paxinos, G., Shimizu, T., Striedter, G., Wild, M., Ball, G.F., Durand, S., Güntürkün, O., Lee, D.W., Mello, C.V., Powers, A., White, S.A., Hough, G., Kubikova, L., Smulders, T.V., Wada, K., Dugas-Ford, J., Husband, S., Yamamoto, K., Yu, J., Siang, C., Jarvis, E.D., Gütürkün, O., Avian Brain Nomenclature Forum, 2004. Revised nomenclature for avian telencephalon and some related brainstem nuclei. J Comp Neurol 473, 377–414. doi:10.1002/cne.20118

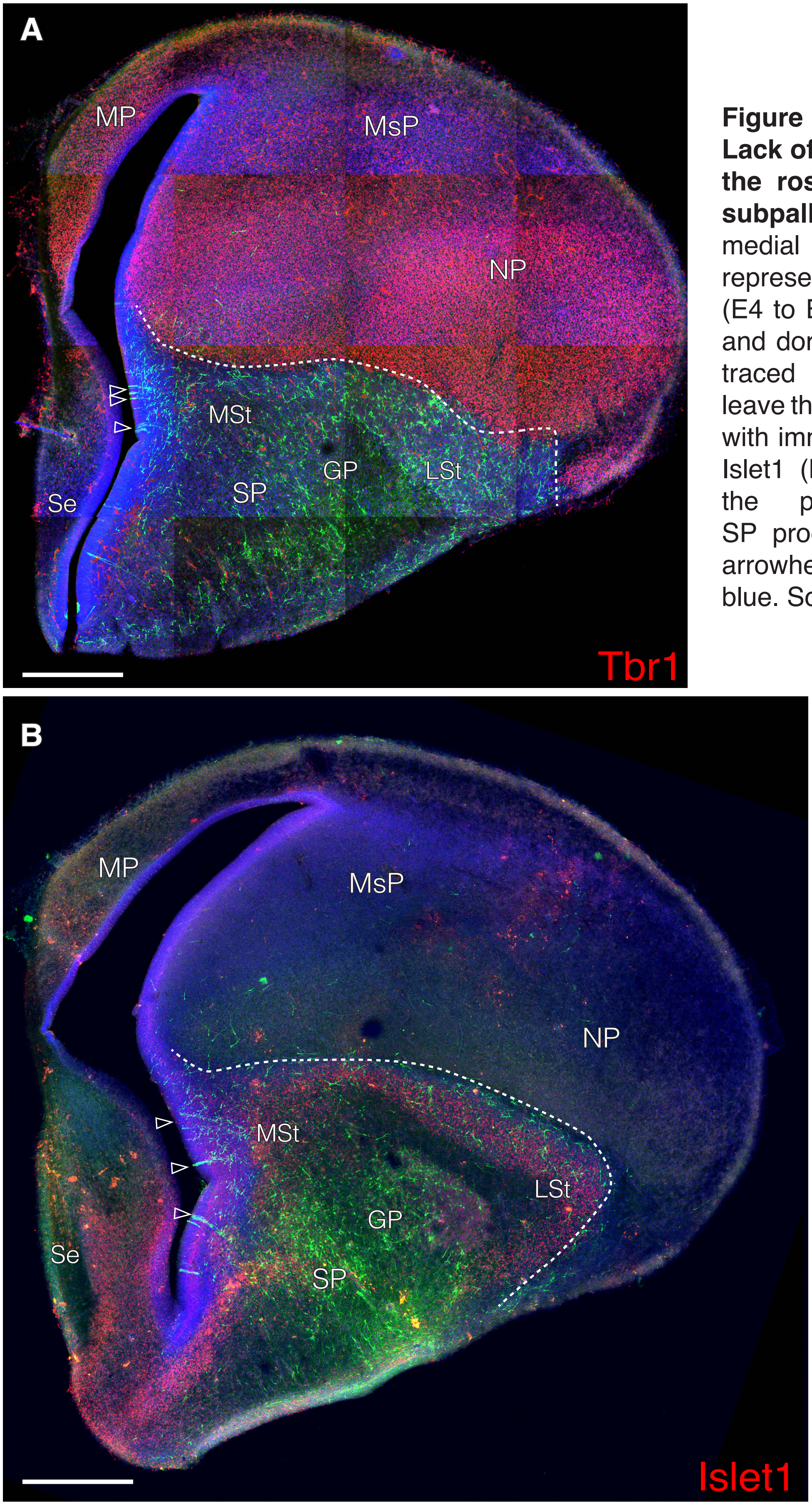

Figure S1. Related to Figure 2 – Lack of tangential migration from the rostral portion of the chick subpallium. Coronal sections, medial at the left. (A,B) Two representative images of long-term (E4 to E11) tracing from the rostral and dorsal subpallium. The lineage traced with GFP (green) did not leave the subpallial limits, as marked with immunostaining for Tbr1 (A) or Islet1 (B). Dashed line represents the pallial-subpallial boundary. SP progenitors are indicated with arrowheads. DAPI counterstain in blue. Scale bars represent 500 µm.

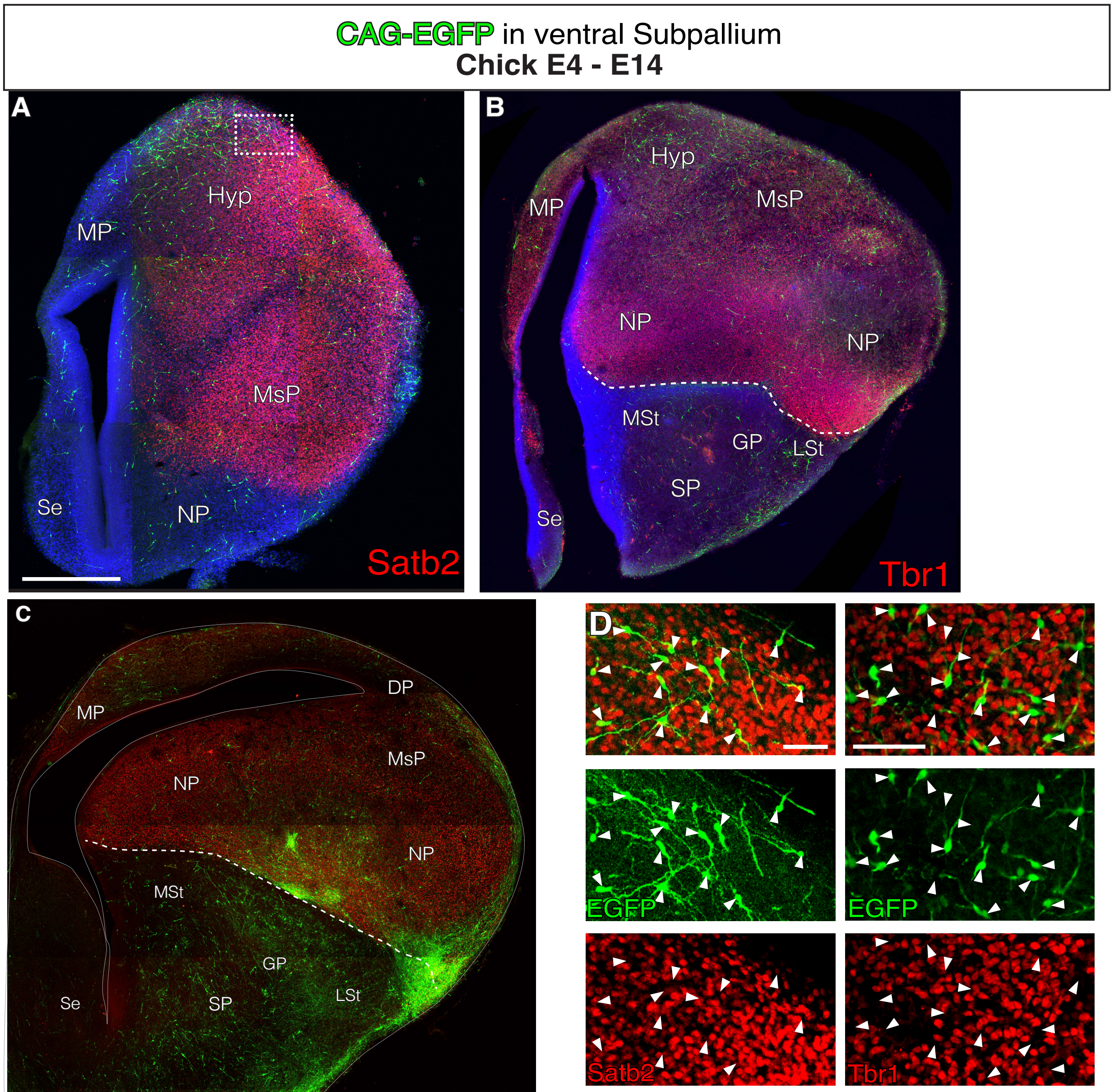

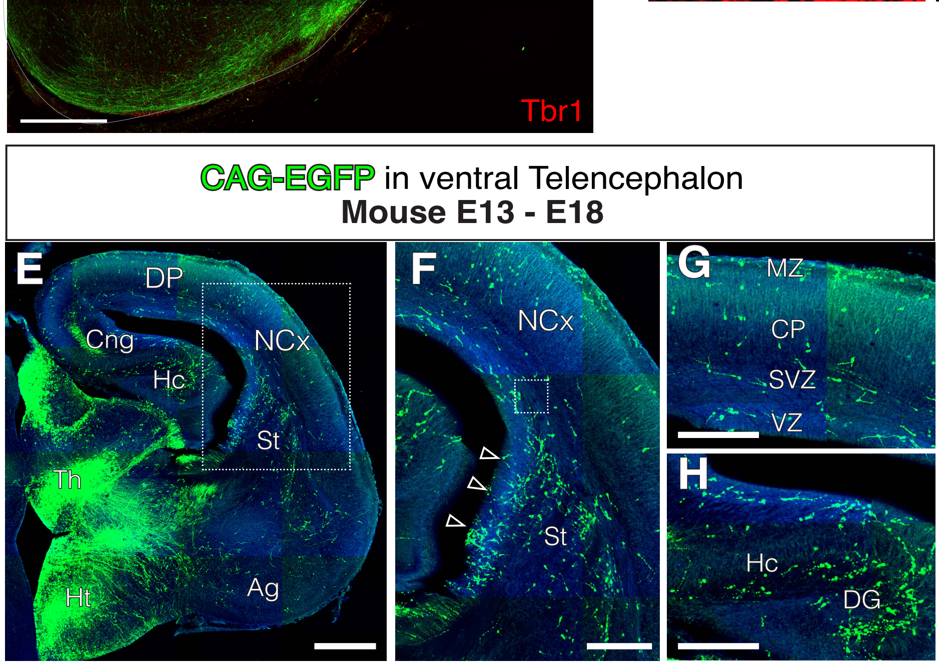

Figure S2. Related to Figure 2 – Tangential migration of interneurons from the cadual portion of the chick subpallium. Coronal sections, medial at the left. (A,B,C) Three representative images of long-term (E4 to E11) tracing from the caudal subpallium. The lineage traced with EGFP (green) migrated to and settled the whole pallium, as marked with immunostaining for Satb2 and Tbr1 (red). Dashed line represents the pallial-subpallial boundary. (D) High magnification showing EGFP derived neurons are neither Satb2 nor Tbr1 immunoreactive (arrowheads). (E-H) Electroporations on mice embryos at E13 on ventral telencephalic regions (including the ventral SP) labeled tangential migratory interneurons. (F) Power view of the rectangle in E. (G-H) Power views from the dorsal neocortex and hippocampus showing the horizontally-oriented tangential interneurons. DAPI counterstain in blue. Scale bars represent 500 µm in A-C,E; 250 µm in F-H; 50 µm in D.

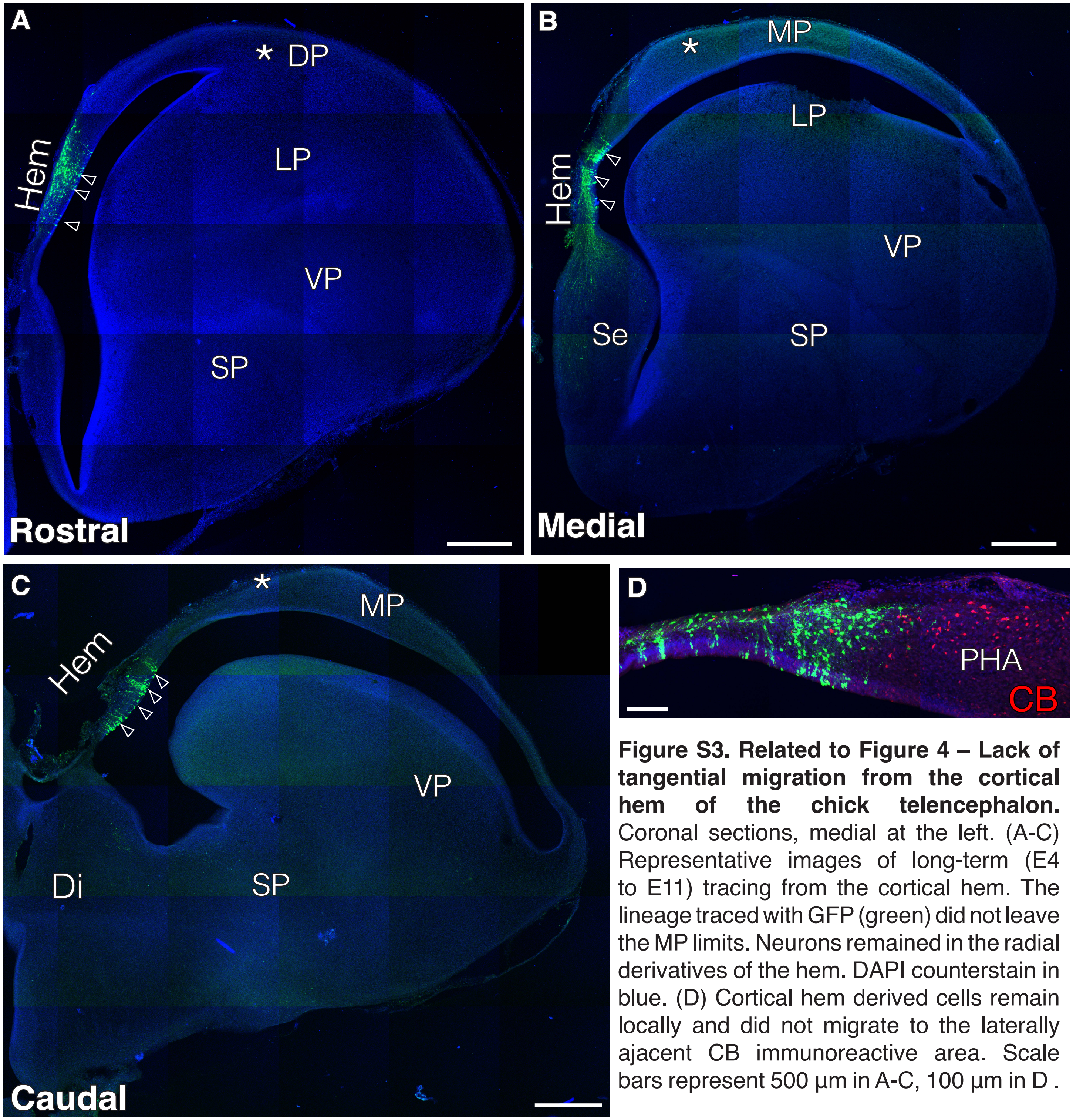

Figure S3. Related to Figure 4 – Lack of tangential migration from the cortical hem of the chick telencephalon. Coronal sections, medial at the left. (A-C) Representative images of long-term (E4 to E11) tracing from the cortical hem. The lineage traced with GFP (green) did not leave the MP limits. Neurons remained in the radial derivatives of the hem. DAPI counterstain in blue. (D) Cortical hem derived cells remain locally and did not migrate to the laterally ajacent CB immunoreactive area. Scale bars represent 500 µm in A-C, 100 µm in D .

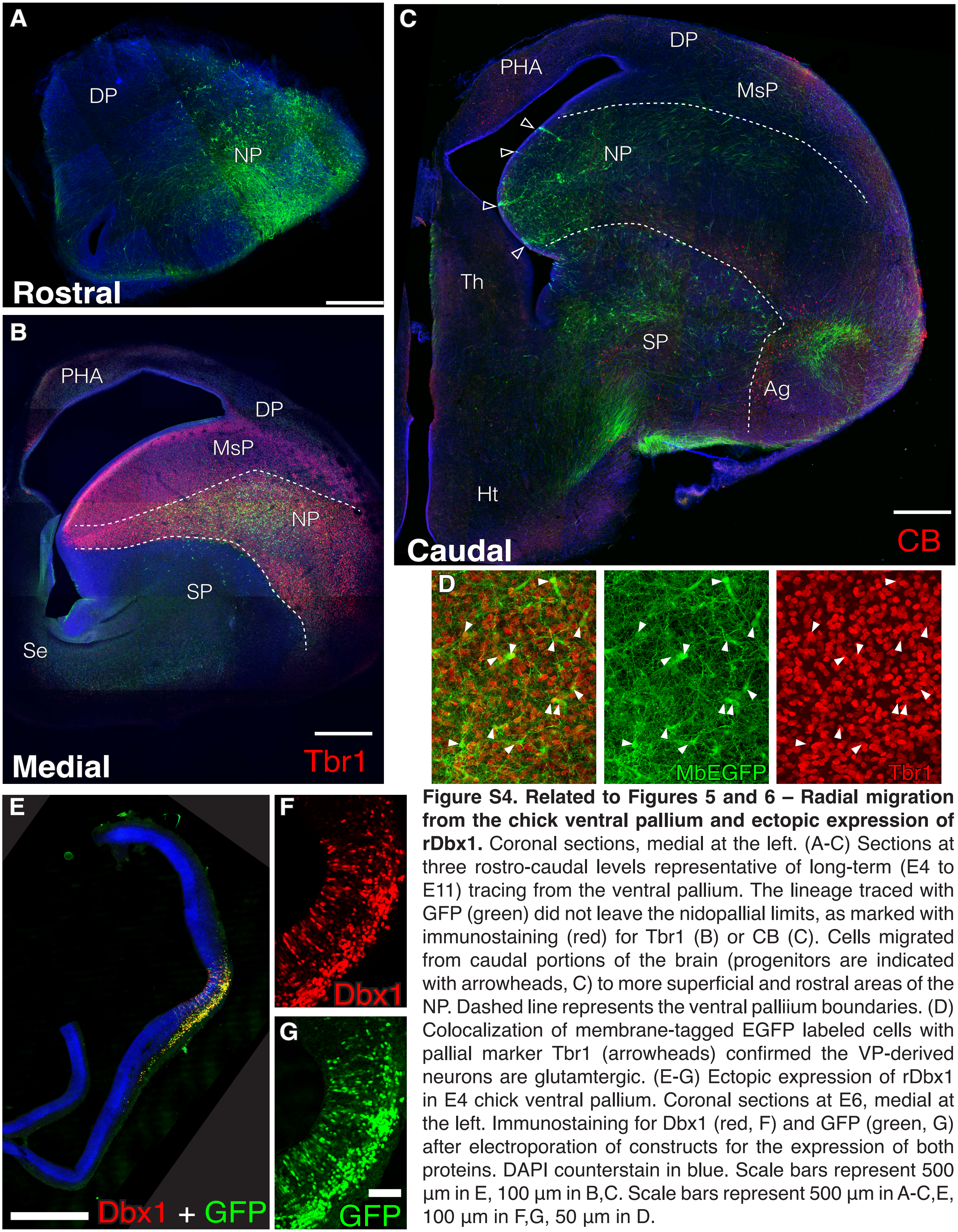

Figure S4. Related to Figures 5 and 6 – Radial migration from the chick ventral pallium and ectopic expression of rDbx1. Coronal sections, medial at the left. (A-C) Sections at three rostro-caudal levels representative of long-term (E4 to E11) tracing from the ventral pallium. The lineage traced with GFP (green) did not leave the nidopallial limits, as marked with immunostaining (red) for Tbr1 (B) or CB (C). Cells migrated from caudal portions of the brain (progenitors are indicated with arrowheads, C) to more superficial and rostral areas of the NP. Dashed line represents the ventral palliium boundaries. (D) Colocalization of membrane-tagged EGFP labeled cells with pallial marker Tbr1 (arrowheads) confirmed the VP-derived neurons are glutamtergic. (E-G) Ectopic expression of rDbx1 in E4 chick ventral pallium. Coronal sections at E6, medial at the left. Immunostaining for Dbx1 (red, F) and GFP (green, G) after electroporation of constructs for the expression of both proteins. DAPI counterstain in blue. Scale bars represent 500 µm in E, 100 µm in B,C. Scale bars represent 500 µm in A-C,E, 100 µm in F,G, 50 µm in D.

Figure S5. Related to Figure 7 – Regulatory landscape of Dbx1. Screenshot from the UCSC browser showing the potential regulatory landscape for the *Dbx1* locus in mouse (mm10). Tracks, from top to bottom: UCSC gene annotations, PhyloP score (placental mammal score; positive-blue indicates high conservation, whereas negative-red report accelerated sequence evolutionary rates), conservation (black/grey blocks show likely homologous regions), transposable element annotation by RepeatMasker, common SNPs, and, for each ATAC-seq sample (E11.5, E12.5 and E14.5), mapped read density, top 120,000 peaks genome-wide, high confidence IDR peaks. Red/blue boxes highlight regions with mammalian-specific/vertebrate-conserved peaks, which are zoomed out below. Orange box highlights a special case of an ATAC-seq peak overlapping largely a mammalian-specific sequence next to a non-coding region conserved across all studied vertebrates except frog.

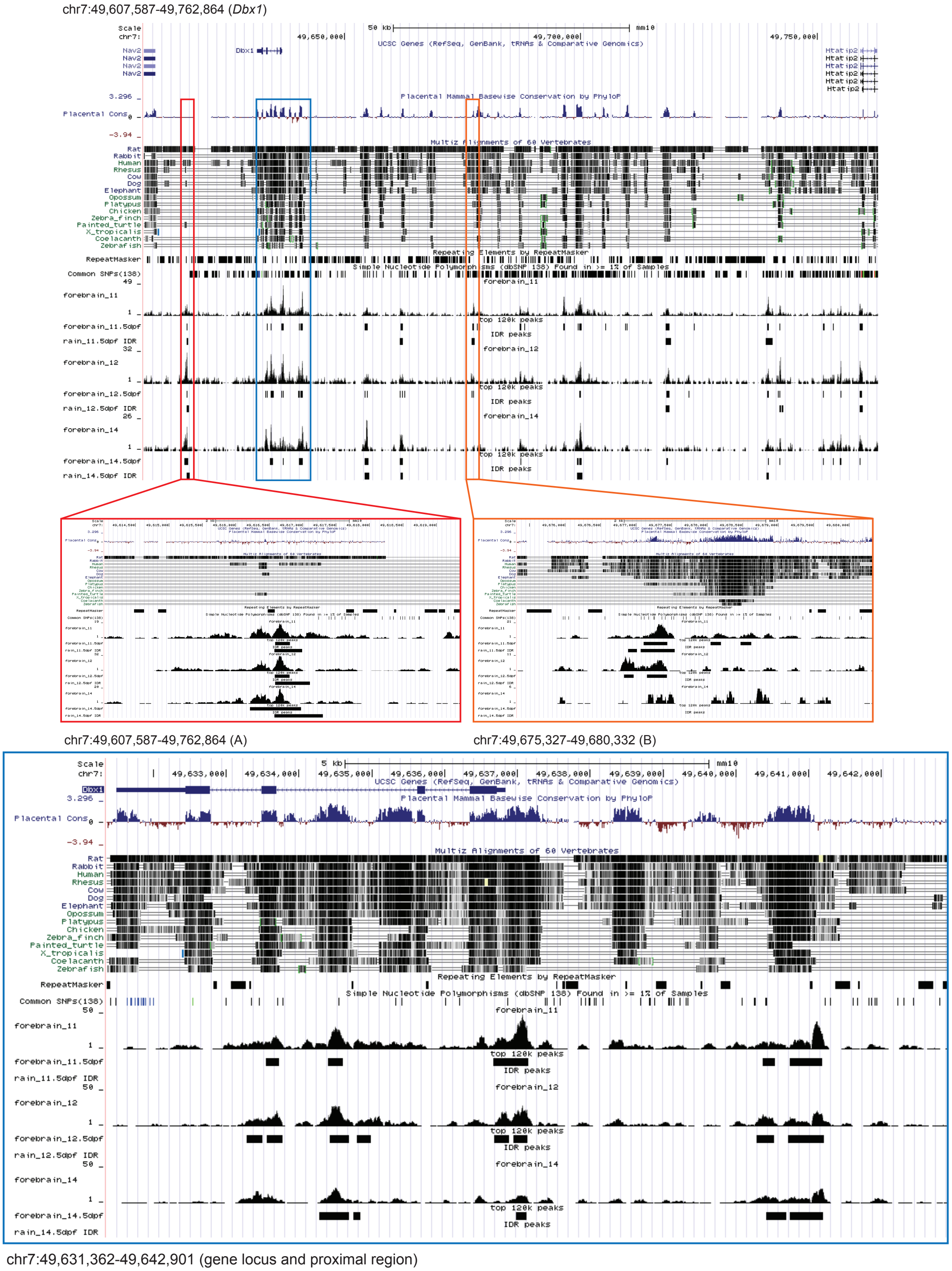

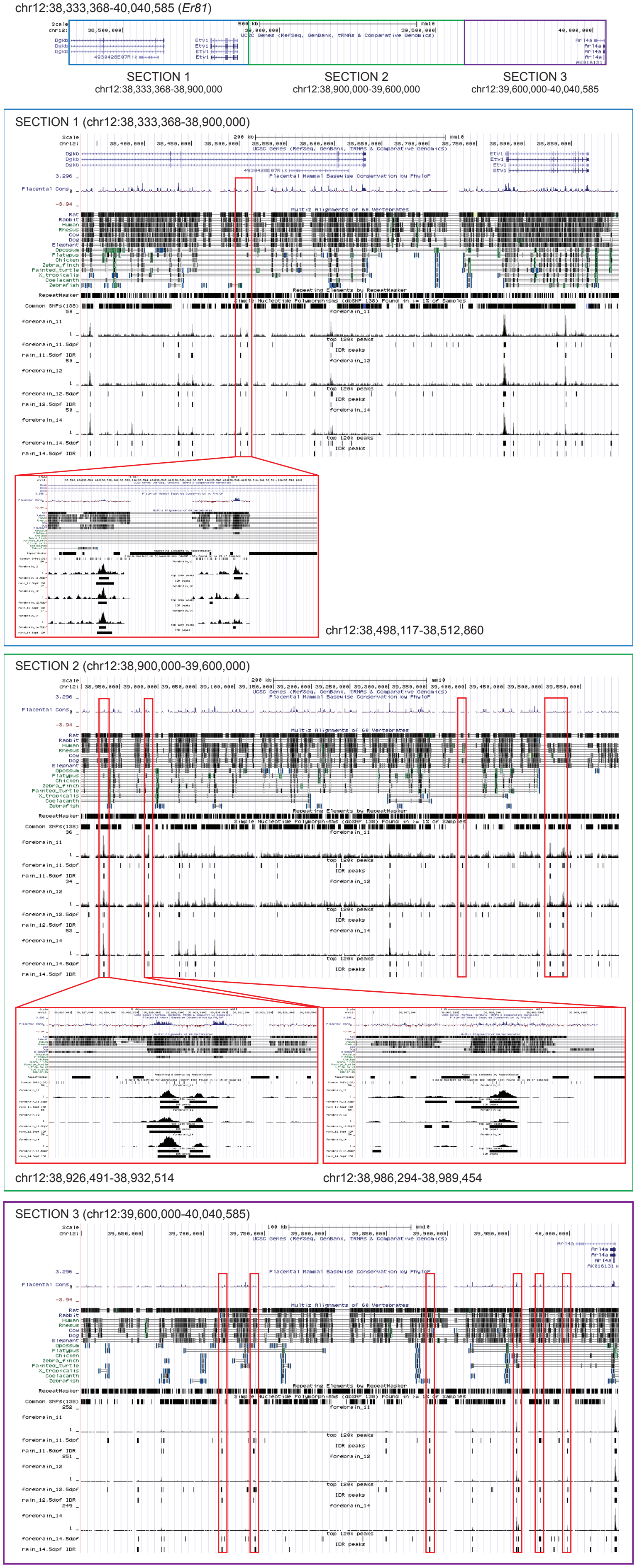

Figure S6. Related to Figure 7 – Regulatory landscape of Er81 (Etv1). The large (~1.7 Mbp) potential regulatory landscape of mouse *Etv1* was divided into three sections (A-C), for which screenshots from the UCSC browser (mm10) are shown below. Tracks, from top to bottom: UCSC gene annotations, PhyloP score (placental mammal score; positive-blue indicates high conservation, whereas negative-red report accelerated sequence evolutionary rates), conservation (black/grey blocks show likely homologous regions), transposable element annotation by RepeatMasker, common SNPs, and, for each ATAC-seq sample (E11.5, E12.5 and E14.5), mapped read density, top 120,000 peaks genome-wide, high confidence IDR peaks. Red boxes highlight regions with mammalian-specific peaks, some of which are enlarged below each section.

chr3:66,873,516-67,365,272 (*Shox2* - *Rsrc1*) chr2:31,617,538-31,670,120 (*Prdm12*)

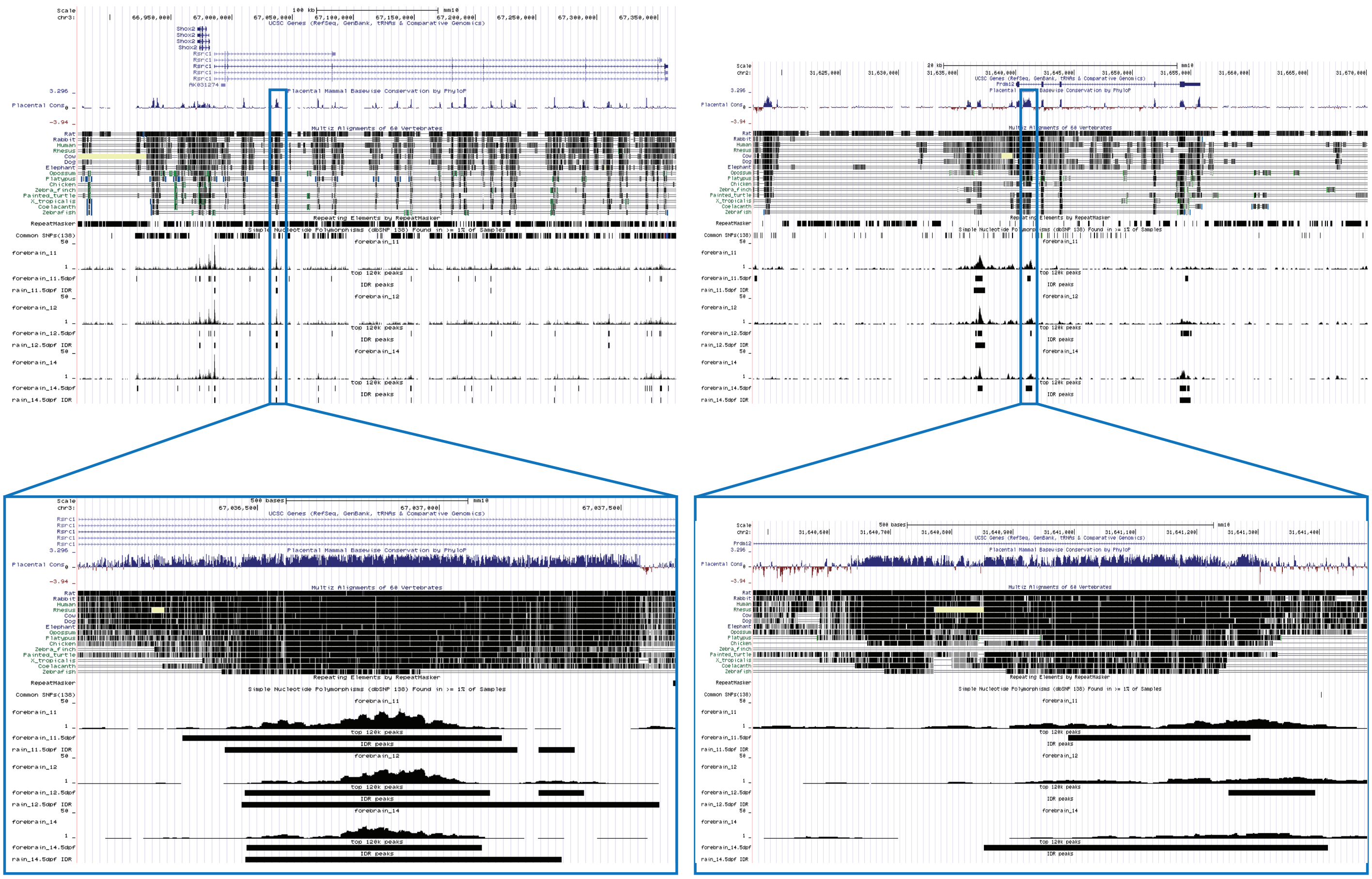

chr3:67,036,007-67,037,651 (hs636) chr2:31,640,477-31,641,478 (hs876)

Figure S7. Related to Figure 7 – Genomic features and conservation analysis of two VISTA enhancers (hs636 and hs876). Screenshot from the UCSC browser showing the genomic context (top) of two VISTA enhancers (hs636 and hs876; bottom). Tracks, from top to bottom: UCSC gene annotations, PhyloP score (placental mammal score; positive-blue indicates high conservation, whereas negative-red report accelerated sequence evolutionary rates), conservation (black/grey blocks show likely homologous regions), transposable element annotation by RepeatMasker, common SNPs, and, for each ATAC-seq sample (E11.5, E12.5 and E14.5), mapped read density, top 120,000 peaks genome-wide, high confidence IDR peaks.
